# Genetic and evolutionary analysis of emerging H3N2 canine influenza virus

**DOI:** 10.1038/s41426-018-0079-0

**Published:** 2018-04-25

**Authors:** Gairu Li, Ruyi Wang, Cheng Zhang, Shilei Wang, Wanting He, Junyan Zhang, Jie Liu, Yuchen Cai, Jiyong Zhou, Shuo Su

**Affiliations:** MOE Joint International Research Laboratory of Animal Health and Food Safety, Jiangsu Engineering Laboratory of Animal Immunology, Institute of Immunology and College of Veterinary Medicine, Nanjing Agricultural University, Nanjing, China

## Abstract

The H3N2 canine influenza virus (CIV) originated from an avian species. Since its emergence, it has circulated in multiple states and has caused pandemics among dog populations; however, no comprehensive studies have explored the causes driving these ongoing cases. The study of the codon usage patterns of viruses can reveal the genetic changes required for the viruses to adapt to new hosts and the external environment. Here we performed a thorough genetic, evolutionary, and codon usage analysis. We identified three evolutionary H3N2 CIV clades from a timescaled phylogenetic tree, namely, Origin, China, and Korea/USA, by principal component analysis (PCA). Additionally, we found a low codon usage bias and that mutation pressure, natural selection, and dinucleotide abundance shape the codon usage bias of H3N2 CIVs, with natural selection being more crucial than the others. Moreover, the human codon adaptation index was similar to that of dogs (the natural host) and cats. In addition, the H3N2 CIV similarity index values were higher than those of the avian influenza virus (AIV), suggesting viral adaptation to the host. Therefore, H3N2 CIVs may pose a potential risk to public health in the future, and further epidemiologic, evolutionary, and pathogenetic studies are required.

## Introduction

Dogs should be carefully considered for their role as influenza hosts because of their close contact with humans and their influenza receptor distribution^1, 2^. H3N8 and H3N2 are the current circulating subtypes of canine influenza virus (CIV) in dog populations. H3N8 CIV, which caused a pandemic in dogs, was first isolated in Florida in 2004^3^, and there were no documented reports of CIV infections prior to 2004^4^. In 2008, Korea first reported H3N2 CIVs, followed by China and Thailand^1, 5, 6^. In April 2015, the H3N2 CIV circulating in Asia was isolated from an infected golden retriever in Cook County, Illinois. The virus has since spread to several states, causing respiratory disease in thousands of dogs across the United States^4^. Of note, a 2012 study showed that a novel H3N1 virus infecting dogs arose by reassortment of a human-origin H1N1 influenza virus and an avian-origin H3N2 CIV. In addition, a 2015 study showed that a novel CIV reassortant, H3N2, containing the polymerase acidic (*PA*) genomic segment from the H9N2 pandemic avian influenza was isolated from a dog in South Korea. These developments suggest that dogs may play critical roles as mixing vessels and reservoirs of avian-origin H3N2 CIVs^7^. Therefore, canines carrying H3N2 CIV may transmit the virus to other species with whom they have frequent close contact, including humans^8^. In addition, companion animals may pose a potential public health risk. Although no H3N2 CIV human infections have been reported, the increasing number of new H3N2 CIV cases in the United States and East Asia, as well as the quick evolution of the virus, calls for detailed genetic analysis of emerging H3N2 CIVs to understand and estimate the risk of CIV adapting to humans.

Phylogenetic and codon usage bias analyses are widely used to investigate viral evolution. For example, Franzo et al.^9^ reported that the feline panleukopenia virus jump to canines leading to canine parvovirus type 2 experienced codon usage adaptation. Butt et al.^10^ found that the codon usage bias analysis was consistent with the phylogenetic analysis of Zika virus (ZIKV). Moreover, Anhlan et al.^11^ reported that codon usage along with phylogenetic analysis helped identify the origin of the 1918 pandemic H1N1 influenza A virus. Choosing synonymous codons is a non-random process. During protein translation, some codons are used more frequently than others, a phenomenon known as “codon usage bias”^12^. Studying codon usage bias can help explain adaptive changes during viral evolution^9^. The codon usage pattern plays a significant role in viral evolution due to species-specific preferences for certain codons^13–15^. A codon usage preference offers an evolutionary force driving the overall viral fitness during replication^16^.

Several studies have been published on the codon usage bias of different viruses. Notably, the codon usage bias is low in most RNA viruses^17, 18^. For instance, the average effective number of codons (ENC) of ZIKV is 53.146 (range 52.559–53.585)^17^. The codon usage bias of Zaire Ebola virus strains is 57.23^19^. The ENC value for Japanese encephalitis virus is 55.30^20^. For H1N1pdm09 influenza A virus (IAV) strains, the average ENC is 52.51, indicating that it is only slightly biased^21^, similar to H5N1 with an ENC value of 50.91 (range of 43.11–55.21)^22^ and the equine influenza virus (EIV), with a mean ENC value of 52.09 (range 47.7–53.72)^23^. However, for the H9N2 avian influenza virus, the codon usage bias is very low, with an ENC ranging from 48.83 to 51.31^24^.

In this study, we comprehensively analyzed the genetic evolution and synonymous codon usage pattern of different H3N2 CIV lineages circulating worldwide between 2006 and 2016. We also investigated the codon usage bias and phylogenetic relationships of H3N2 CIVs based on sequence data from past and current outbreaks in Asia and the United States. Our findings show novel perspectives on the molecular evolution of H3N2 CIVs.

## Results

### Evolutionary analysis of H3N2 CIV complete genomes

**Fig. 1:**
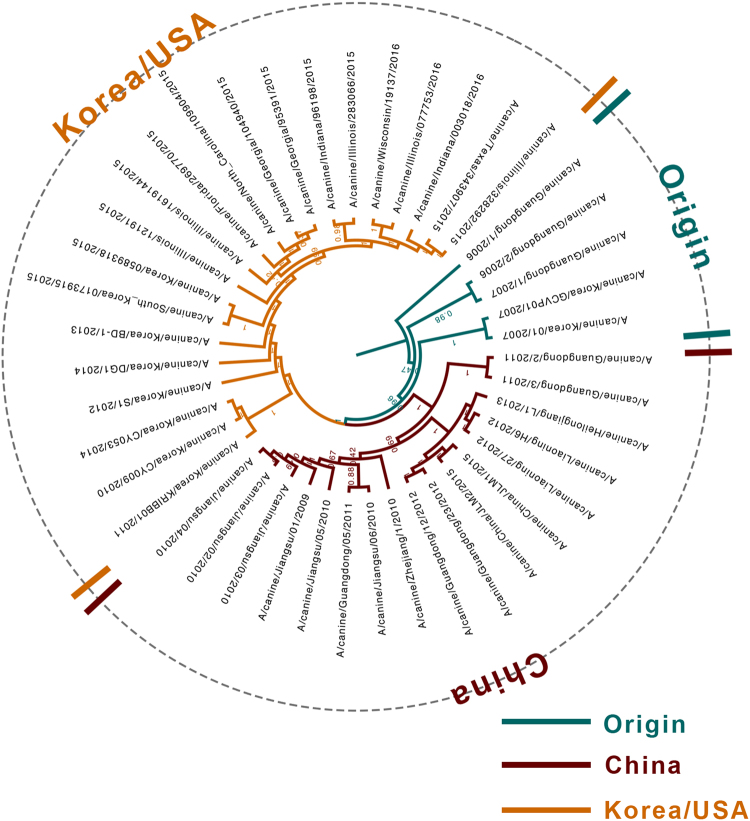
Phylogenetic tree of the complete coding sequences of H3N2 CIVs based on BEAST 1.8.0. Origin, China, and Korea/USA clades are represented in green, red, and orange, respectively

Among the 48 selected complete genomes, 5 were previously reported as reassortment events^4, 25, 26^, and low-quality genomes (Table S1) were excluded from the analysis. Therefore, 43 CIV genomes were used to infer the phylogenetic tree (Fig. 1) and codon usage. Phylogenetic analysis revealed that since the outbreak of H3N2 CIV in 2006 in Guangdong, China, un-reassortant H3N2 CIV mainly circulated in three countries, namely, China, Korea and the United States. Based on the tree structure, three clades, namely, Origin, China, and Korea/USA were identified, with the Origin clade including the earlier strains prevailing in China and Korea. The recent isolated strains from China and Korea/USA were clustered with the China and Korea/USA clades, accordingly.

### A and U nucleotides are more abundant in the H3N2 CIV genomes

Nucleotide compositions are commonly used to demonstrate bias in genes/genomes. Here we found that the most frequent mononucleotide was A, with a mean ± standard deviation (SD) value of 33.59% ± 0.25. The next most common mononucleotides was G, U, and C with mean ± SD values of 23.26% ± 0.242, 22.41% ± 0.056, and 20.75% ± 0.065, respectively. The composition of AU was higher than that of GC, with 56.0% ± 0.283 compared with 44.01% ± 0.283. Additionally, the frequency of codons at the third position suggested that A_3s_ (42.3% ± 0.007) and U_3s_ (31.0% ± 0.002) were more abundant synonymous codons than G_3s_ (26.3% ± 0.007) and C_3s_ (27.5% ± 0.002). The average GC contents at the first, second, and third positions (GC_12s_ and GC_3s_) were 45.29% and 40.97%, respectively. Furthermore, the nucleotide composition calculated by specific phylogenetic clades, which was also in accordance with the total composition, showed that A and U were rich among the three clades. The detailed compositions of the H3N2 CIV genomes are shown in Table S2. The unequal nucleotide composition observed in the H3N2 CIV clades suggested codon usage bias.

### Codon usage bias of H3N2 CIV coding sequences and differences in specific clades

**Fig. 2:**
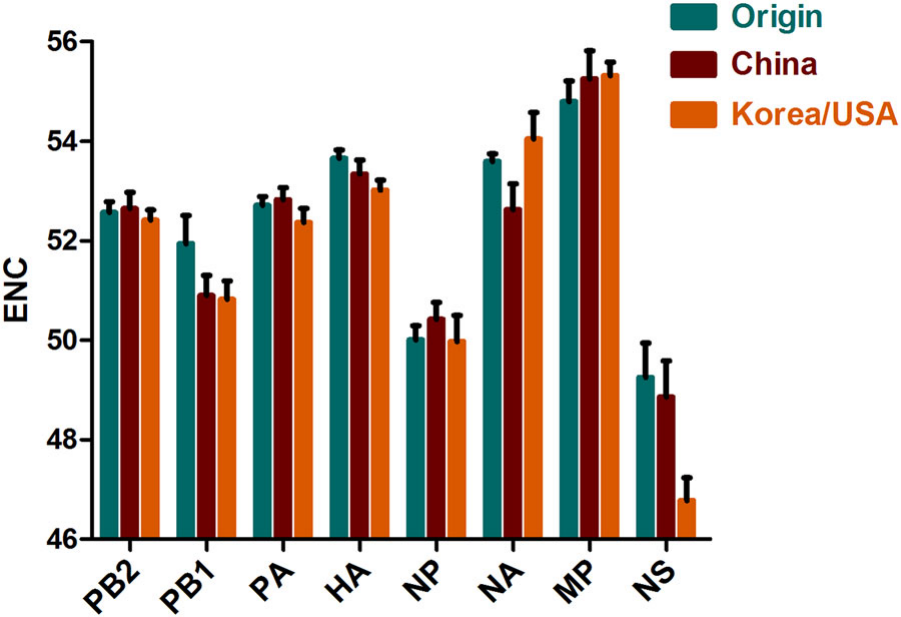
ENC values in H3N2 CIV segments. The H3N2 CIV strains are clustered into three genotypic groups, namely, Origin, China, and Korea/USA, represented in green, red, and orange, respectively

To infer the magnitude of the H3N2 CIV codon usage choice, the ENC values were calculated. ENC values ranged from 52.740 to 53.420, with a mean value ± SD of 53.053 ± 0.192, indicating a lower codon usage choice in all strains. In addition, the mean ENC values were 53.326, 53.151, and 52.903 for the Origin, China, and Korea/USA clades, respectively. Analyzing individual genomic segments showed that* MP* had the highest ENC value (55.24 ± 0.44), while *NS* had the lowest (47.89 ± 1.25) (Fig. 2). Interestingly, the *NS* gene had the highest ENC value in the Korea/USA clade. Overall, the H3N2 CIV ENC value was >35, indicating a significantly low codon usage bias. Moreover, a low degree of codon usage bias was identified among the clades and individual genes.

### Relative synonymous codon usage analysis of H3N2 CIV genomes

Relative synonymous codon usage (RSCU) analysis is commonly applied to explore the synonymous codon usage pattern. Here we found that among the most frequently used synonymous codons, 14 were A/U-ended codons (9 terminated in A), and the numbers of U-, C-, and G-ended codons were 5, 2, and 1, respectively, with no bias in Tyr (Table 1). Importantly, 5 of the 18 optional synonymous codons were overrepresented with RSCU values higher than 1.6. Furthermore, all were A-terminated codons (CCA, ACA, GCA, AGA, and GGA). No optional synonymous codons were underrepresented (RSCU < 0.6). We next analyzed the synonymous codon tendencies among the clades and found that A/U-ended codons were more common than G/C-ended codons. This was in accordance with the occurrence of all strains, regardless of clade, except for the optional codon encoding for Leu, which was CUU in all China and Korea/USA clade strains and CUG in the Origin clade. Regarding Tyr, no bias was found for UAU and UAC in all strains, whereas the preferred codon was UAC in the Origin and China clades and UAU in the Korea/USA clade. We then explored the relationship between H3N2 CIV and its natural host, dogs, as well as its potential hosts including cats, chickens, and humans. We found that the 18 most abundant host codons were not completely harmonious with the H3N2 CIV codons.

**Table 1:** RSCU of H3N2 CIVs, genotypes, and potential hosts

		H3N2 CIVs				Potential host			
AA	Codon	All	Original	China	Korea	*Homo sapiens*	*Canis familiaris*	*Felis catus*	*Gallus gallus*
Phe	UUU(F)	0.84	0.81	0.84	0.84	0.93	0.82	0.77	0.91
	UUC(F)	**1.16**	**1.19**	**1.16**	**1.16**	**1.07**	**1.18**	**1.13**	**1.09**
Leu	UUA(L)	0.65	0.61	0.63	0.68	0.46	0.35	0.35	0.45
	UUG(L)	1.07	1.14	1.1	1.04	0.77	0.71	0.76	0.81
	CUU(L)	**1.21**	1.19	**1.22**	**1.19**	0.79	0.70	0.67	0.80
	CUC(L)	0.9	0.91	0.89	0.91	1.17	1.30	1.29	1.08
	CUA(L)	1	0.91	0.96	1.06	0.43	0.39	0.36	0.38
	CUG(L)	1.16	**1.24**	1.2	1.12	**2.37**	**2.56**	**2.57**	**2.48**
Ile	AUU(I)	**1.15**	**1.12**	**1.14**	**1.16**	1.08	0.96	0.95	1.06
	AUC(I)	0.8	0.82	0.81	0.79	**1.41**	**1.60**	**1.58**	**1.39**
	AUA(I)	1.05	1.06	1.05	1.05	0.51	0.45	0.47	0.55
Val	GUU(V)	0.99	0.97	0.98	0.99	0.73	0.58	0.62	0.84
	GUC(V)	0.76	0.78	0.76	0.76	0.95	1.07	1.13	0.87
	GUA(V)	0.9	0.86	0.93	0.88	0.47	0.41	0.38	0.50
	GUG(V)	**1.35**	**1.38**	**1.33**	**1.37**	**1.85**	**1.94**	**1.87**	**1.80**
Ser	UCU(S)	0.98	0.99	0.97	0.98	1.13	1.08	1.12	1.09
	UCC(S)	0.93	0.92	0.92	0.94	1.31	1.45	**1.48**	1.21
	UCA(S)	**1.39**	**1.35**	**1.39**	**1.39**	0.90	0.77	0.74	0.89
	UCG(S)	0.37	0.37	0.38	0.36	0.33	0.36	0.38	0.40
	AGU(S)	1.08	1.09	1.07	1.1	0.90	0.85	0.80	0.86
	AGC(S)	1.25	1.28	1.28	1.23	**1.44**	**1.49**	1.47	**1.55**
Pro	CCU(P)	1.16	1.16	1.17	1.15	1.15	1.08	1.03	1.10
	CCC(P)	0.74	0.73	0.74	0.74	**1.29**	**1.42**	**1.51**	**1.22**
	CCA(P)	**1.62**	**1.57**	**1.58**	**1.67**	1.11	1.01	0.97	1.13
	CCG(P)	0.48	0.54	0.51	0.44	0.45	0.48	0.50	0.56
Thr	ACU(T)	1.01	1.02	1.02	1.01	0.99	0.89	0.84	0.99
	ACC(T)	0.98	0.99	0.97	0.99	**1.42**	**1.55**	**1.59**	**1.23**
	ACA(T)	**1.82**	**1.8**	**1.82**	**1.82**	1.14	1.03	0.94	1.20
	ACG(T)	0.19	0.19	0.2	0.18	0.46	0.52	0.63	0.57
Ala	GCU(A)	1.05	1.05	1.05	1.05	1.06	1.00	0.96	1.16
	GCC(A)	0.78	0.79	0.79	0.77	**1.60**	**1.75**	**1.79**	**1.27**
	GCA(A)	**1.89**	**1.87**	**1.87**	**1.91**	0.91	0.79	0.76	1.06
	GCG(A)	0.28	0.29	0.29	0.26	0.42	0.46	0.50	0.51
Tyr	UAU(Y)	**1**	0.97	0.99	**1.01**	0.89	0.79	0.78	0.80
	UAC(Y)	**1**	**1.03**	**1.01**	0.99	**1.11**	**1.21**	**1.22**	**1.20**
His	CAU(H)	**1.22**	**1.27**	**1.26**	**1.19**	0.84	0.78	0.74	0.80
	CAC(H)	0.78	0.73	0.74	0.81	**1.16**	**1.22**	**1.26**	**1.20**
Gln	CAA(Q)	**1.11**	**1.04**	**1.11**	**1.12**	0.53	0.50	0.56	0.54
	CAG(Q)	0.89	0.96	0.89	0.88	**1.47**	**1.50**	**1.44**	**1.46**
Asn	AAU(N)	**1.11**	**1.1**	**1.13**	**1.11**	1.08	0.96	0.82	0.86
	AAC(N)	0.89	0.9	0.87	0.89	**1.41**	**1.60**	**1.18**	**1.14**
Lys	AAA(K)	**1.25**	**1.21**	**1.25**	**1.25**	0.87	0.79	0.86	0.89
	AAG(K)	0.75	0.79	0.75	0.75	**1.13**	**1.21**	**1.14**	**1.11**
Asp	GAU(D)	**1.1**	**1.12**	**1.11**	**1.1**	0.93	0.86	0.84	**1.01**
	GAC(D)	0.9	0.88	0.89	0.9	**1.07**	**1.14**	**1.16**	0.99
Glu	GAA(E)	**1.14**	**1.09**	**1.14**	**1.15**	0.84	0.79	0.86	0.86
	GAG(E)	0.86	0.91	0.86	0.85	**1.16**	**1.21**	**1.14**	**1.14**
Cys	UGU(C)	0.92	0.9	0.93	0.92	0.91	0.85	0.87	0.80
	UGC(C)	**1.08**	**1.1**	**1.07**	**1.08**	**1.09**	**1.15**	**1.13**	**1.20**
Arg	CGU(R)	0.23	0.23	0.22	0.25	0.48	0.44	0.41	0.59
	CGC(R)	0.33	0.34	0.35	0.32	1.10	1.21	1.09	1.14
	CGA(R)	0.59	0.56	0.58	0.6	0.65	0.64	0.55	0.58
	CGG(R)	0.59	0.62	0.6	0.58	1.21	1.25	1.19	1.07
	AGA(R)	**2.66**	**2.61**	**2.66**	**2.68**	**1.29**	1.19	1.33	**1.34**
	AGG(R)	1.6	1.64	1.6	1.58	1.27	**1.27**	**1.41**	1.29
Gly	GGU(G)	0.56	0.58	0.55	0.56	0.65	0.65	0.58	0.70
	GGC(G)	0.59	0.61	0.61	0.58	**1.35**	**1.39**	**1.42**	**1.22**
	GGA(G)	**1.82**	**1.77**	**1.8**	**1.84**	1.00	0.97	1.01	1.09
	GGG(G)	1.03	1.04	1.04	1.02	1.00	1.00	0.99	0.99

Preferred H3N2 CIV codons, genotypes, and potential hosts are displayed in bold

Additionally, we compared the RSCU value of the *HA* gene of H3N2 CIVs to those of avian and human influenza viruses and found that among the 18 abundant synonymous codons, most synonymous codons were the same across all influenza virus species (Table S3). We also discovered that UUG, encoding Leu, was abundant relative to codon usage. Interestingly, UUG was the synonymous codon encoding amino acid 222 for Leu in the *HA* gene, which is important in receptor binding.

### The synonymous codon usage pattern tendency varied and was specific to individual clades

**Fig. 3:**
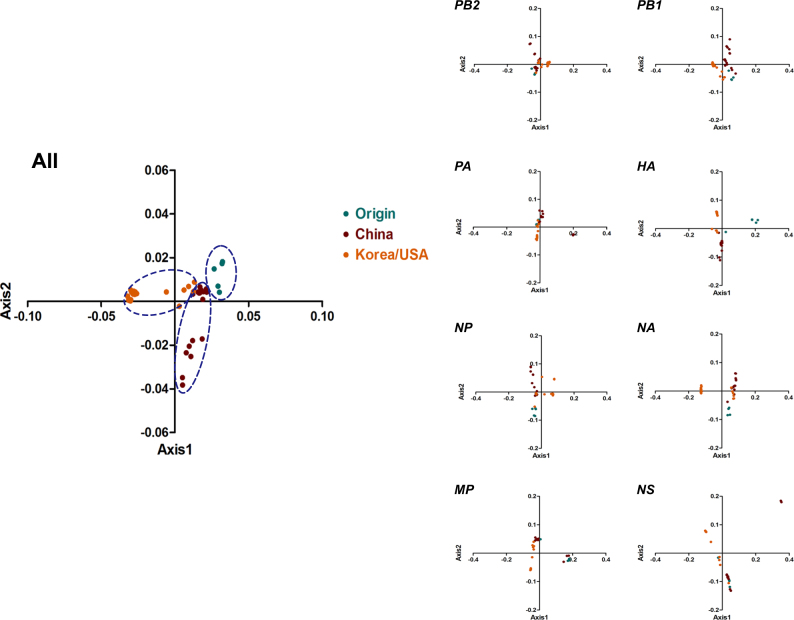
PCA based on the RSCU values of the 59 synonymous codons. Genotype-specific PCA was structured for whole genomes and H3N2 (eight segments). Origin, China, and Korea/USA clades are represented in green, red, and orange, respectively

To study the synonymous codon usage variation in the complete coding sequences of H3N2 CIVs, we performed principal component analysis (PCA). The first four principal axes (axes 1–4) accounted for 80.9%, specifically 54.21%, 16.05%, 6.67%, and 3.97%, respectively (Fig S1). Axes 1 and 2 were significant in determining the synonymous codon usage pattern (Fig. 3). All strains were mainly clustered into three groups, consistent with the phylogenetic tree topology. The Origin clade was mainly distributed in group 1, the China clade mainly belonged to group 2, and the Korea/USA clade belonged to group 3. In addition, we performed PCA of individual genes based on clades. We found that the different clade strains partially overlapped, except for the *HA* and *NA* genes, especially in the Origin clade. Above all, the clades were related during evolution, specifically the recent China and Korea/USA outbreak strains.

### The potential roles of mutation pressure and natural selection in shaping the H3N2 CIV codon usage bias

**Fig. 4:**
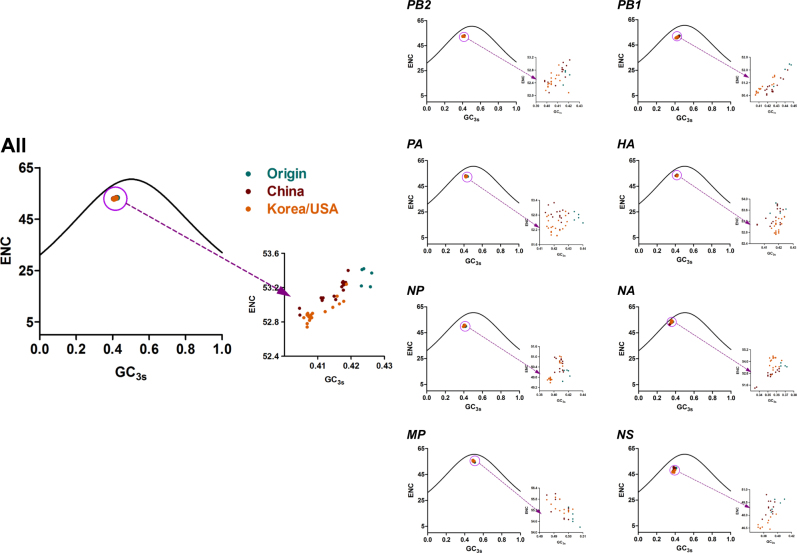
Relationship between ENC and GC_3s_ of whole genomes and different H3N2 segments. Solid curves represent the stranded ENC. Origin, China, and Korea/USA clades are represented as green, red, and orange diamonds, respectively

Previous studies underlined that mutation pressure and natural selection were key factors driving the codon usage bias of individual genes or genomes; thus, to prove the hypothesis for the complete H3N2 CIV coding sequences, ENC plots were constructed according to the defined clades and individual genes (Fig. 4). In the complete coding sequence plot, all strains sat lower than the expected curve. In addition, the China clade overlapped with the Korea/USA clade, while the Origin clade was distributed in a single group. Furthermore, ENC plots were drawn based on individual genes. The plot showed that all clades mixed together for the eight individual genes, except the Origin clade that separated from other clades for the *PA* gene. Interestingly, several Korea/USA clade strains in the *NA* gene sat near the expected curve, suggesting that mutation pressure played a major role in this strain. Overall, ENC plots elucidated that both mutation pressure and natural selection are essential in driving the codon usage bias of the complete genomes of H3N2 CIVs.

To confirm the above results, we performed regression analysis among the nucleotide compositions, sense codon content, Axis1, Axis2, Aroma, and Gravy (Table 2). Nucleotides, codons, and ENC were significantly correlated (*p* < 0.01). Axis2 correlated with nucleotide and codon composition (*p* < 0.05). Axis1 correlated with several indices, including A, C_3_, U_3_, and GC_3_. Additionally, the relationship between Gravy and A, G, A_3_, G_3_, GC, GC_3_, and ENC was remarkable. Aroma only correlated with mononucleotide U at 0.01 < *p* < 0.05. This emphasizes that mutation pressure and natural selection shape the codon usage propensity.

**Table 2:** Correlation analysis among nucleotide composition, codon composition, ENC, Aroma, Gravy, Axis1, and Axis2

	A	C	G	U	U_3s_	C_3s_	A_3s_	G_3s_	GC	GC_3s_	ENC	Gravy	Aroma	Axis1	Axis2
A															
C	−0.598**														
G	−0.996**	0.568**													
U	0.536**	−0.934**	−0.537**												
U_3s_	0.628**	−0.841**	−0.629**	0.870**											
C_3s_	−0.690**	0.905**	0.667**	−0.843**	−0.907**										
A_3s_	0.992**	−0.570**	−0.986**	0.499**	0.576**	−0.673**									
G_3s_	−0.986**	0.506**	0.987**	−0.454**	−0.557**	0.627**	−0.993**								
GC	−0.986**	0.713**	0.982**	−0.670**	−0.725**	0.774**	−0.972**	0.957**							
GC_3s_	−0.989**	0.650**	0.984**	−0.594**	−0.690**	0.763**	-0.988**	0.981**	0.988**						
ENC	−0.903**	0.490**	0.902**	−0.438**	−0.489**	0.627**	−0.917**	0.911**	0.881**	0.903**					
Gravy	−0.529**	−0.134	0.548**	0.157	−0.089	0.042	−0.502**	0.550**	0.435**	0.458**	0.570**				
Aroma	−0.060	−0.178	0.025	0.349*	0.165	−0.071	−0.083	0.092	−0.017	0.051	−0.033	−0.059			
Axis1	−0.892**	0.277	0.902	−0.245	−0.358*	0.432**	−0.906	0.933	0.832	0.871**	0.898**	0.745**	0.021		
Axis2	−0.415**	0.827**	0.382*	−0.755**	−0.649**	0.710**	−0.405**	0.334*	0.516**	0.467**	0.229	−0.452**	0.106	0.000	

*0.05 < *p* < 0.01; **p < 0.01, the individual number represents the correlation coefficient of the index

### Natural selection is the major driver of H3N2 CIV codon usage

Given the conclusion that mutation pressure and natural selection were important drivers of H3N2 CIV codon usage bias in the complete coding sequences, we sought to determine the mutation pressure-natural selection equilibrium using parity rule 2 (PR2) analysis (Fig S2). We found that AU-optional degenerate codons were preferred over GC codons in the frequently used fourfold degenerate codon families (Ala, Arg, Gly, Leu, Pro, Ser, Thr, and Val). Most importantly, the magnitudes of mutation pressure and natural selection varied.

**Fig. 5:**
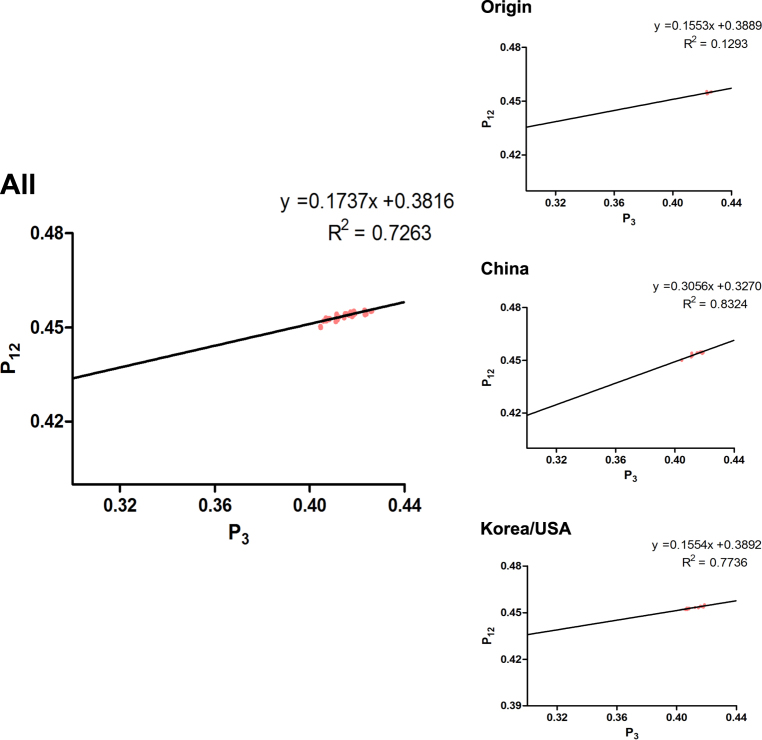
Neutrality plot analysis (GC_3s_ against GC_12s_) of the complete coding sequences of H3N2 CIVs and different clades .

Next, we performed neutrality analyses to explain the decisive role in choosing codon usage in H3N2 CIV complete coding sequences (Fig. 5). *P*_12_ (GC_12s_) and *P*_3_ (GC_3s_) were significantly correlated (*r* = 0.852, *p* = 0.0001) when analyzing all strains, with a correlation coefficient of 0.1737 ± 0.3816. Thus, the mutation pressure (relative neutrality) was 17.37%, whereas natural selection (relative constraint on *P*_3_) was 82.63%. Based on individual clade analysis, the linear regression slopes were 15.53% for the Origin clade (*r* = 0.36), 30.56% for the China clade (*r* = 0.553), and 15.54% for the Korea/USA clade (*r* = 0.394). This indicates that the role of natural selection is more crucial than mutation pressure in shaping the H3N2 CIV codon usage bias. We also analyzed the specific coding sequences of the eight independent segments by evolutionary clades. We found that the coefficients between *P*_12_ and *P*_3_ were positively correlated, except that for the *NA* gene, which was −0.06997. Moreover, mutation pressure predominated over natural selection in the China clade for the* MP* and *NS* coding sequences (the mutation pressure percentage was 50.66% compared with 72.55% for natural selection).

In summary, although we found different constraints of natural selection in distant clades and individual genes, natural selection was more significant in driving the codon usage bias of the complete coding sequences of H3N2 CIVs.

### Host-specific codon usage pattern of H3N2 CIVs

To investigate the adaptation of individual H3N2 CIV genes to their natural hosts and potential hosts, we performed codon adaptation index (CAI) analysis (Table S4). CAI represents the relationship between gene expression levels and codon usage patterns. The CAI values of the eight genes related to the potential hosts were calculated for all selected strains. We found that the CAI values of the different hosts varied. Specifically, chickens displayed the highest in all eight individual genes, with mean values of 0.7695 for the *HA* gene, 0.7657 for *MP*, 0.7434 for *NA*, 0.7456 for *NP*, 0.745 for *NS*, 0.7728 for *PA*, 0.7883 for *PB1*, and 0.756 for *PB2*. In addition, at the evolutionary clade level, the highest CAI values were also observed in chickens, followed by humans, felines, and canines, regardless of clade.

We also performed similarity index (SiD) analysis (Fig S3). The overall levels and specific clades were studied based on potential hosts. Compared with the other three potential hosts, canines were crucial to H3N2 CIV evolution. Moreover, the Korea/USA clade was largely influenced, followed by the China and Origin clades.

### H3N2 CIV displayed a deoptimized codon usage pattern from its potential hosts

To identify the codon usage deoptimization of H3N2 CIV coding sequences with their potential hosts, the relative codon deoptimization index (RCDI) values were calculated for the individual segments as well as the evolutionary clades. The RCDI values were >1 relative to the four potential hosts, not considering the independent segments and specific clades, suggesting a deoptimization codon usage pattern relative to the potential hosts (Table S5). Higher RCDI values were observed in the feline and canine species. In detail, except for the *MP* gene relative to the feline species, which was the highest among the four hosts, the highest deoptimization codon usage was observed in the canine species for the other seven genes. Additionally, the *NS* gene was considered the most deoptimized from its potential hosts.

### Evolution and natural selection of *HA* and *NA* genes on H3N2 CIV cross-species transmission

**Fig. 6:**
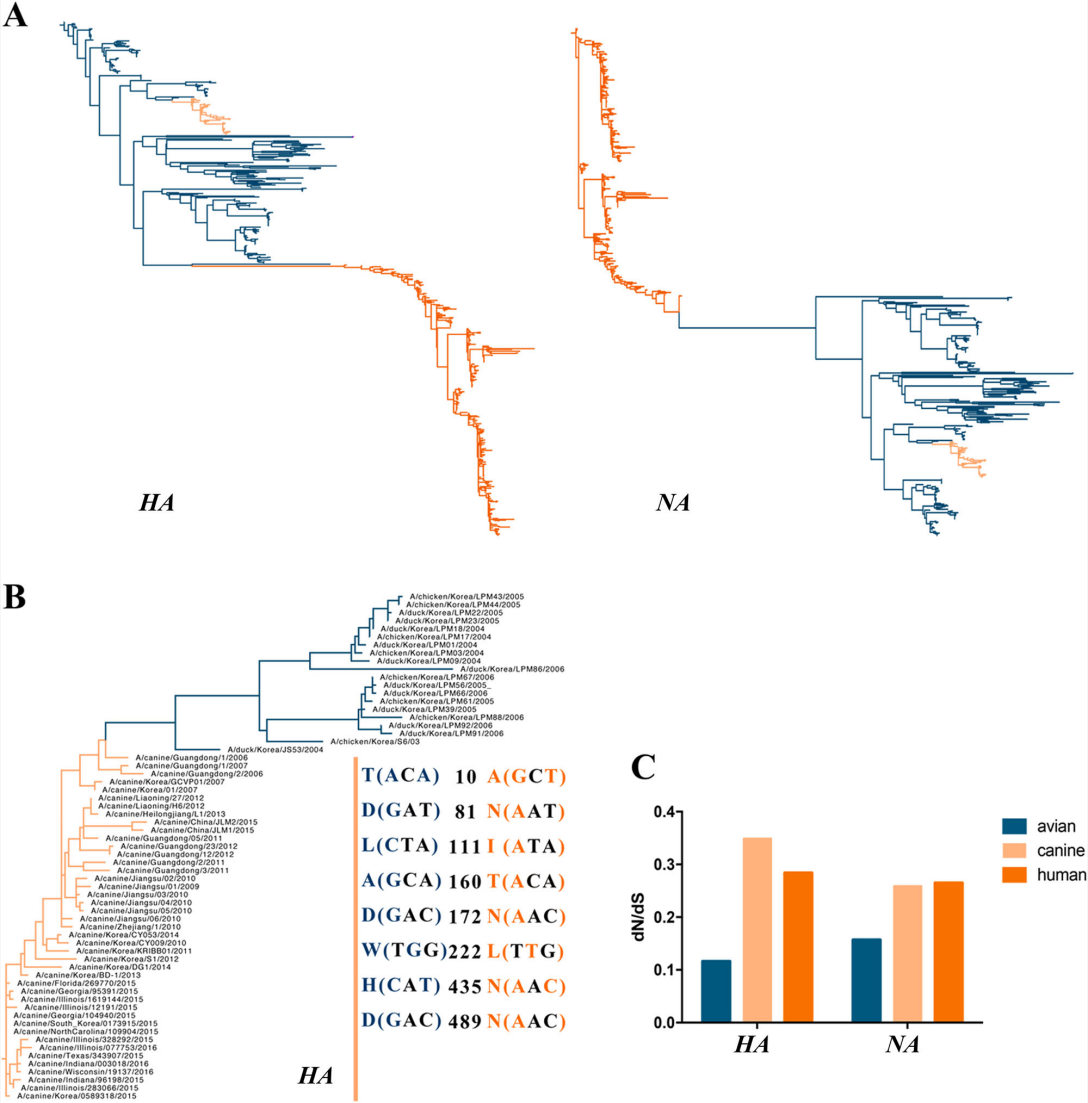
ML phylogenetic trees of the *HA* and *NA* **(A) **genes reconstructed with 597 sequences and phylogenetic tree of the *HA* **(B) **gene constructed with 43 H3N2 CIVs and 20 AIVs and the d*N*/d*S* **(C)** value of the influenza viruses (including avian, canine, and human) corresponding to the *HA* and *NA* genes (H3N2 AIVs are blue, CIVs are pink, and the human influenza virus is orange)

The *HA* and *NA* phylogenetic trees showed that H3N2 CIVs are closely related to the avian influenza virus H3N2 (Fig. 6A), indicating an avian origin, consistent with a previous study^26^. Additionally, 20 sequences that were closely related to H3N2 CIV were chosen to detect the relationship between H3N2 CIV and AIV (Fig. 6B). Previous studies^26, 27^showed that eight amino-acid sites in the *HA* gene, namely, T(ACA)10A(GCT), D(GAT)81N(AAT), L(CTA)111I(ATA), A(GCA)160T(ACA), D(GAC)172N(AAC), W(TGG)222L(TTG), H(CAT)435N(AAC), and D(GAC)489N(AAC), differed in the avian precursor virus compared to H3N2 CIV. Moreover, six of the mutated amino acids were due to A mutations, which were abundant among H3N2 CIVs. Of note, our analysis revealed consistently more non-synonymous substitutions per site compared with reservoir H3N2 AIVs (Fig. 6C). In H3N2 CIVs, the mean d*N*/d*S* values were 0.349 in *HA* and 0.259 in *NA*, whereas the equivalent values in the avian viral forms were 0.116 for *HA* and 0.157 for *NA*. This might be due to host-specific selection after transfer.

### Dinucleotide abundance influences the codon usage bias of H3N2 CIV coding sequences

We calculated the occurrence of the 16 dinucleotide frequencies to understand their influence in codon usage choice (Fig S5). We found that all dinucleotides were non-random, with no dinucleotide being overrepresented, while dinucleotides CpG and UpA were underrepresented (<0.7). In the RSCU analysis, both CpG (UCG, CCG, ACG, GCG, CAG, CGU, CGC, and CGG) and UpA (UUA, CUA, AUA, GUA, UCA, UAU, UAC, and AUU) were not optional in the relative amino acids. Furthermore, no overrepresented codons were found, except for the codons encoding Tyr (UAU and UAC). This demonstrated that dinucleotide occurrence affects the codon usage bias of the complete coding sequences of H3N2 CIVs.

## Discussion

The H3N2 CIV genome undergoes evolutionary changes. Two vital patterns contribute to influenza A virus evolution: shift and drift^26^. The occurrence of mutations over time leading to evolutionary drift can be explained by codon usage bias. Here 43 strains from different geographical areas were thoroughly analyzed to reveal the synonymous codon usage patterns and the evolutionary history of H3N2 CIVs. The analysis was conducted for individual segments and specific clades. Previously identified reassortment sequences^4, 25, 26,]^ or low-quality sequences were excluded since they can mislead tree topology. According to the timescaled phylogenetic tree, three clades were identified, namely, the Origin clade, the China clade, and the Korea/USA clade. The phylogenetic tree topology was also consistent with our another unpublished study about H3N2 CIV evolution and a previous study^4^. Hence, it is interesting to note that the PCA distribution of complete coding sequences was consistent with the phylogenetic structure, supporting the idea that PCA can reflect the evolutionary clade classification from the perspective of codon usage analysis^28^.

Previous studies typically concentrated on calculating the nucleotide/codon frequency to determine codon usage bias^23, 29^. The H3N2 CIV complete coding sequences were rich in A/U in triplet codons and mononucleotides. This phenomenon demonstrated that codon usage bias plays a role in H3N2 CIV evolution. We then measured the degree of codon usage bias. We found a low ENC value for the H3N2 CIV genome, indicating a low codon usage bias. Other studies have also reported low codon usage biases for other influenza A viruses, such as H3N8 EIV (mean value of 52.09)^23^, the H5N1 influenza virus (mean value of 50.91)^22^, and H1N1pdm IAV (mean value of 52.5)^11^. In addition, other RNA viruses also show low codon biases, including ZIKV (mean value of 53.93)^10^, Marburg virus (mean value of 54.2), and Ebola virus (mean value of 57.23)^19^. An explanation for low codon usage bias in RNA viruses is that it reduces the resistance of host cells to viral genome replication^10^. Therefore, we hypothesized that the low codon usage bias of H3N2 CIV could have contributed to the CIV epidemic. Mutation pressure and natural selection are commonly thought to be major drivers of codon usage bias. ENC plots depicted that all strains were under the expected curve regardless of individual genes or clade evolution, demonstrating that in addition to mutation pressure and natural selection, other forces contribute to codon usage. In addition, correlation analysis of codon usage indices suggests the existence of mutation pressure and natural selection. Therefore, we investigated how mutation pressure and natural selection govern codon usage. Using the PR2 method, we found that A/U-terminated codons predominated over G/C-terminated codons in the fourfold degenerated codon families, which was consistent with the finding that A/U-ended codons were abundant in the complete coding sequences of H3N2 CIVs. The same phenomenon was observed when the analysis was performed using individual genes, although the A/U termination differed among the genes, indicating that the eight genes diverged during evolution. Analysis of EIV H3N8 showed that natural selection was a stronger force than mutation pressure^23^. Using neutrality analysis, we found that the natural selection percentage constrained the codon usage by 82.63% compared to 17.37% for mutation pressure. Additionally, *P*_12_ and *P*_3_ were significantly correlated. Importantly, detailed descriptions of the eight individual genes also confirmed that natural selection dominated over mutation pressure.

The phenomenon that A/U-preferred codons governed the selection pressure of the complete coding sequences of H3N2 CIVs was confirmed not only via the codon composition analysis but also by PR2 analysis. Additionally, optional codon usage was previously reported to largely rely on the relative host(s), which was reflected by RSCU analysis between viruses and their corresponding host(s)^10^. In the present study, RSCU analysis of H3N2 CIVs with their natural host (canines) and potential hosts (humans, felines, and chickens) revealed discordant codon usage of H3N2 CIVs. This was consistent with reports on ZIKV^10^ but distinct from the completely unanimous patterns observed for the poliovirus^30^ and hepatitis A virus^31^. The complex codon usage pattern of H3N2 CIVs relative to its hosts can be explained because consistent synonymous codons allow sufficient protein translation, while inconsistent codon usage allows proper protein folding^32^. CAI analysis revealed that the viral expression level adapts to the corresponding host(s). A higher CAI indicates strong adaptation^33^, which strongly demonstrates natural selection. The CAI values were highest in chickens, followed by humans, canines, and felines. These results might be because H3N2 CIVs originated from avian species and then were transmitted to canines and felines, with the potential risk of human infection. For example, other studies have previously reported that house cats are reservoirs of influenza and can spread it to humans;^8, 34^ however, this requires further investigation. Additionally, using SiD and RCDI analysis, we discovered that canines and felines significantly shaped the H3N2 CIV codon usage compared with chickens and humans, which may be because canines and felines have been crucial in the H3N2 CIV epidemics.

Based on the combined results of the CAI, SiD, and RCID analyses, we discovered a low codon usage bias in the *HA* gene, possibly due to the role of *HA* gene in triggering immune response^35^. Therefore, we hypothesized that H3N2 CIV originated from the avian influenza A virus, followed by transmission to canines and felines with successful preliminary adaptation in dogs. Interestingly, a previous study showed that the USA H3N2 CIVs and the recent strains isolated in South Korea exhibited changes in the receptor binding and antigenic sites of the *HA* gene (Gly to Ser change in position 146 in the Korea/USA clade)^4^. Consistent with this, the Korea/USA sequences displayed the highest relationship between H3N2 CIV and its hosts. Moreover, CAI analysis suggested that the eight segments are also adapted to the human species similar to canines and felines. Thus, the H3N2 CIVs circulating in the canine population may acquire amino-acid changes, particularly in the receptor-binding sites (changes from a-2,3-linked sialic receptors to a-2,6-linked sialic receptors)^34^, which could aid adaptation to humans. Canine-specific mutations that differentiate H3N2 CIV from avian reservoir viruses have been reported in previous studies^26, 27^. Of note, we discovered that most mutant amino sites among H3N2 CIV compared with the avian precursor virus involved A changes. In addition, amino acid 222 was located at the H3N2 CIV receptor-binding site. RSCU analysis of different influenza viruses (avian, canine, and human) revealed that UUG (encoding amino acid 222) was abundant, demonstrating that codon usage can reveal viral evolution. Emerging viruses were considered to undergo host-specific selection after transferring to new hosts, thus improving the viral replication and transmissibility. Apart from mutation pressure and natural selection, other factors, such as dinucleotide abundance, are considered to influence codon usage bias. We found no overrepresented dinucleotides in the H3N2 CIV genome, but CpGs and UpAs were underrepresented. Unmethylated CpGs, recognized by the intracellular pattern recognition receptor-toll-like receptor 9, are believed to trigger the immune response^36^. Therefore, low CpG use can be a means of evading immune responses. Since CpG underrepresentation is attributed to the U/A-biased codons in single-stranded negative-sense RNA viruses, the dinucleotide usage influences the codon usage bias. Since UpAs were underrepresented, dinucleotide abundance was minor in driving the H3N2 CIV codon usage bias.

In conclusion, this study shows that from the first identified H3N2 CIV strains in 2006 to the recent epidemic, natural selection, mutation pressure, and dinucleotide abundance shape the codon usage of H3N2 CIVs. Specifically, natural selection was more important than the other factors. Interestingly, previous studies found that during pet-to-human transmission, H7N2 AIV receptor-binding sites were changed^34^. The codon usage analysis performed here, both on the hosts and on the H3N2 CIVs, allowed us to hypothesize that during long-term circulation, H3N2 CIVs may cause potential future risks to companion hosts and veterinary personnel. Therefore, more epidemiologic surveys, systemic evolutionary analyses, and monitoring are needed.

## Materials and Methods

### Sequence information

Forty-eight complete genomes, including eight segmented complete H3N2 CIV coding sequences, deposited in the Influenza Virus Resource at the National Center for Biotechnological Information (http://www.ncbi.nlm.nih.gov/genomes/FLU/FLU.html) were analyzed in this study. Additionally, 259 complete avian influenza virus *HA* and *NA* genes and 295 complete human influenza virus *HA* and *NA* genes were also analyzed. Detailed information, including accession number, strain name, collection date, and country of isolation, is listed in supplemental material Table S1. Additionally, the complete coding sequences were concatenated in the following order: *PB2* + *PB1* + *PA* + *HA* + *NP* + *NA* + *MP* + *NS*.

### Reassortment event removal and phylogenetic tree reconstruction

Reassortment events confuse the codon usage pattern^37, 38^ and phylogenetic tree topology;^39^ therefore, reassortant strains were removed based on previous studies and evidenced from the phylogenetic trees. Alignments were performed using Muscle. Timescaled phylogenetic trees were reconstructed using BEAST (version 1.8.0)^40^. We used Bayesian Markov Chain Monte Carlo (MCMC) method to infer phylogenetic trees. The optimal nucleotide substitution model was estimated using MEGA 7.0. The HKY85 plus gamma distribution substitution model was used in BEAST. Additionally, Bayesian skyline coalescent prior was assumed with a relaxed lognormal clock. The MCMC chains were run twice independently for 10^8^ generations and sampled every 10 000 steps. Runs were further combined using LogCombiner software^41^. Using the TreeAnnotator program in the BEAST package, a target tree was summarized by selecting the tree with the largest posterior probability with a 10% burn-in. Additionally, 597 complete H3N2 *HA* and *NA* sequences, including 43 CIV sequences, 259 complete avian influenza virus sequences, and 295 complete human influenza virus sequences, were used to reconstruct the maximum likelihood phylogenetic tree using RAxML^42^.

### Codon usage parameters

#### Nucleotide and codon usage composition

To explore codon usage bias, the nucleotide frequencies of A, U, C, and G and the total content of AU and GC were calculated using BioEdit. Additionally, the GC contents at the first, second, and third synonymous codon positions (GC_1s_, GC_2s_, and GC_3s_) were calculated by Emboss: cusp^43^. The codon compositions at the third position (A_3_%, U_3_%, C_3_%, and G_3_%) were computed using the CodonW 1.4.2 package. Five non-bias codons were excluded, namely, AUG and UGG, since they are the only codons encoding Met and Trp, respectively, and UAA, UAG, and UGA, which encode termination codons.

#### Effective number of codons

The ENC value, which indicates the degree of codon usage bias, ranges from 20 to 61. The smaller the ENC value is the stronger is the bias, and a value of 35 is the cutoff point (<35 indicates significant bias and vice versa^44^). The ENC value was calculated as follows:

$${\mathrm{ENC}}\, = \,2\, + \frac{9}{{{F}_2}}\, + \,\frac{1}{{{F}_3}}\, + \,\frac{5}{{{F}_4}}\, + \,\frac{3}{{{F}_6}}$$

where *F*_*i*_ (*i* = 2, 3, 4, 6) represents the mean value of *F*_*i*_ for *i*-fold degenerate codon families. The *F*_*i*_ value was calculated using the following formula:

$$F_i\, = \,\frac{{\mathop {\sum }\nolimits_{j = 1}^i (\frac{{n_j}}{n})^2 - 1}}{{n - 1}}$$

where *n* is the total number of codon occurrences for a given amino acid and *n*_*j*_ is the total number of *j*th codon frequencies for that amino acid.

#### Relative synonymous codon usage

The synonymous codon usage bias was also evaluated using RSCU values, which can accurately express the synonymous codon frequencies, avoiding the confounding factors of amino-acid composition and coding sequence length. The RSCU was calculated as follows:

$${\mathrm{RSCU}}\, = \,\frac{{X_{ij}}}{{\mathop {\sum }\nolimits_j^{n_i} X_{ij}}}n_i$$

where *X*_*ij*_ is the number of occurrences of the *i*th codons for the *j*th amino acid and *n*_*i*_ is the number of synonymous codons that encode the *i*th amino acid. An RSCU value > 1.0 represents positive codon usage bias, while a value <1.0 indicates negative bias. Additionally, synonymous codons with RSCU values > 1.6 and <0.6 suggest “overrepresented” and “underrepresented” codons, respectively^45^. RSCU values were calculated using MEGA 7.0.

### Effect of mutation pressure on codon usage bias

#### ENC-plot analysis

To explore the factors influencing codon usage bias, ENC-plot analysis was performed by plotting ENC values against GC_3s_. Plotting ENC-GC_3s_ near the standard curve of the expected ENC values suggests a specific role of mutation pressure driving codon usage bias; otherwise, other factors are contributing. The expected ENC was calculated as follows:

$${\mathrm{ENC}}_{{\mathrm{expected}}} = 2 + s + \frac{{29}}{{s^2 + (1 - s^2)}}$$

where “*s*” is the frequency of G + C at the third codon position of synonymous codons.

#### Neutrality analysis

The relationship between GC_12s_ ($P_{12}$) and GC_3s_ ($P_3$), referred to as the neutrality analysis, was used to identify the roles of mutation and natural selection on codon usage bias. GC_12s_ was plotted against GC_3s_, and linear regression was calculated by GraphPad Prism 5.0. An equation slope near 1 indicates that GC_12s_ and GC_3s_ are significantly correlated, and mutation pressure is the dominant factor^46^. Conversely, if the value tends to the abscissa or the ordinate, natural selection is the dominant factor.

### Natural selection on codon usage bias

#### Aroma and Gravy indices

Aroma and Gravy were used to understand the factors influencing codon usage. Aroma indicates the aromatic amino-acid frequency, while Gravy indicates the hydropathic amino-acid frequency. The values range from −2 to 2^23^ and were calculated using the CodonW package.

#### CAI analysis

The H3N2 CIV codon usage patterns were compared to those of different host species (*Homo sapiens*, *Canis familiaris, Felis catus*, and *Gallus gallus*). The host data were extracted from the Codon Usage Database (http://www.kazusa.or.jp/codon/). The CAI value by reference codon usage database of the different potential hosts ranged from 0 to 1. The calculation method was performed as previously described^47^ (http://genomes.urv.cat/CAIcal/RCDI/), and the five non-bias codons were excluded. High values indicate that the codon usage bias of the strain is significant and more adaptable to that host^33^.

#### RCDI analysis

The RCDI value of eight segmented complete coding sequences of H3N2 CIV strains were calculated by the RCDI/eRCDI SERVER (http://genomes.urv.cat/CAIcal/RCDI/)^47^. The five non-bias codons were excluded. The reference codon usage table of potential hosts was obtained from the codon usage database (http://www.kazusa.or.jp/codon/). All above data were used to determine the codon deoptimization trends. An RCDI value of >1 means that the H3N2 CIV codon usage pattern is adapted to the host. Unlike CAI, if the value is higher, it indicates that the codon usage pattern deviates from the host^48^.

#### SiD analysis

To determine the H3N2 CIV codon usage bias compared with the codon usage for different host species, SiD analysis was performed. The value was calculated as follows:

$${R}\left( {{\mathrm{A}},{\mathrm{B}}} \right) = \frac{{\mathop {\sum }\nolimits_{{i} = 1}^{59} {a}_{i} \ast {b}_{i}}}{{\sqrt {\mathop {\sum }\nolimits_{{i} = 1}^{59} {a}_{i}^2 \ast \mathop {\sum }\nolimits_{{i} = 1}^{59} {b}_{i}^2} }}$$

$${D}\left( {{\mathrm{A}},{\mathrm{B}}} \right)\, = \,\frac{{1 - {R}({\mathrm{A}},{\mathrm{B}})}}{2}$$

where *a*_*i*_ is the RSCU value of 59 synonymous codons from the H3N2 CIV complete coding sequences and *b*_*i*_ is the RSCU value of the identical codons in the potential host. *R* (A, B) is defined as the cosine of the angle between A and B and represents the degree of similarity between the H3N2 CIVs and the host codon usage patterns. *D* (A, B) represents the potential effect of the overall codon usage of the host. Higher SiD values are thought to suggest stronger host adaptation.

### Principal component analysis

PCA, a multivariate statistical method, was used to reflect the major tendency of the synonymous codon usage pattern in the H3N2 CIV coding sequences. The RSCU values were transformed into small numbers of uncorrelated variables (59-dimensional vector), which were principal components represented by the axis, and the percentage of each axis was calculated by CodonW. To detect H3N2 CIV coding sequence clustering, the first two axes were plotted (Axis1 against Axis2 of each strain).

### PR2 analysis

PR2 analysis, corresponding to the fourfold degeneration codons, is frequently used to understand the roles of mutation pressure and natural selection in driving complete gene codon usage^49^. It entails plotting the relationships of the codons at the third position, A_3_/(A_3_ + U_3_), in the abscissa against G_3_/(G_3_ + C_3_) in the ordinate. If A = U and G = C, the values sit in the center of the plot (both coordinates are 0.5), indicating equal roles of mutation pressure and selection rates (substitution rates);^50^ otherwise, bias exists.

### Correlation analysis

Pearson correlations among the A%, U%, G%, C%, A_3s_, U_3s_, G_3s_, C_3s_, GC_3s_, ENC, Aroma, Gravy, Axis1, and Axis2 were calculated using GraphPad Prism 5. The correlation coefficients and *p* values were used to test significance. *p* values < 0.01 and 0.05 < *p* *<* 0.01 denote extremely significant correlations and significant correlations, respectively. Mean and SDs were calculated using Microsoft Excel, and the differences in ENC were calculated using analysis of variance.

### d*N*/d*S* analysis

d*N*/d*S* values of all reported avian influenza viral strains and the selected CIV and human influenza virus strains were estimated using DATAMONKEY (http://www.datamonkey.org/). Alignments were performed using Muscle after removing termination codons.

### Dinucleotide frequency analysis

The frequencies of 16 dinucleotides per strain were calculated using DAMBE software. The ratio was calculated as follows:

$${P}_{{\mathrm{xy}}}\, = \,\frac{{{r}_{{\mathrm{xy}}}}}{{{r}_{\mathrm{y}}{r}_{\mathrm{x}}}}$$

where *r*_x_ and *r*_y_ represent the frequency of nucleotides X and Y, respectively, while *r*_xy_ represents the observed dinucleotide XY value, and *r*_y_*r*_x_ represents the expected value of the dinucleotide XY. It is normally considered that the XY dinucleotide is overrepresented when *P*_xy_ is >1.23 and underrepresented when it is <0.78^51^.

## Electronic supplementary material

Table s

FIGURE S1

FIGURE S2

FIGURE S3

FIGURE S4

FIGURE S5
