# Supplementary material for: Genetic and evolutionary analysis of emerging H3N2 canine influenza virus
Source: Emerg Microbes Infect. 2018 Apr 25;7:73. doi: 10.1038/s41426-018-0079-0 (PMC5915587; doi:10.1038/s41426-018-0079-0)
Supplement: Supplementary file 1 — Table s [file 41426_2018_79_MOESM1_ESM.docx]

Table S1. The detailed information of the H3N2 CIVs used in this study.

| Strain name | HA | M | NA | NP | NS | PA | PB1 | PB2 | Host | Country | Date |
| --- | --- | --- | --- | --- | --- | --- | --- | --- | --- | --- | --- |
| A/canine/China/JLM1/2015 | MG386676 | MG386677 | MG386678 | MG386679 | MG386680 | MG386681 | MG386682 | MG386683 | canine | China | 2015 |
| A/canine/China/JLM2/2015 | MG386684 | MG386685 | MG386686 | MG386687 | MG386688 | MG386689 | MG386690 | MG386691 | canine | China | 2015 |
| A/canine/Florida/269770/2015 | MF173382 | MF173281 | MF173401 | MF173216 | MF173142 | MF173220 | MF173194 | MF173191 | canine | Florida | 2015 |
| A/canine/Georgia/104940/2015 | KX571026 | KX571051 | KX570966 | KX571049 | KX570977 | KX570982 | KX570997 | KX571013 | canine | Georgia | 2015 |
| A/canine/Georgia/95391/2015 | KX571027 | KX570980 | KX570998 | KX571004 | KX570991 | KX571037 | KX570969 | KX570974 | canine | Georgia | 2015 |
| A/canine/Guangdong/05/2011 | JX414244 | JX414245 | JX414246 | JX414247 | JX414248 | JX414249 | JX414250 | JX414251 | canine | Guangdong | 2011 |
| A/canine/Guangdong/1/2006 | GU433345 | GU433346 | GU433347 | GU433348 | GU433352 | GU433349 | GU433350 | GU433351 | canine | Guangdong | 2006 |
| A/canine/Guangdong/1/2007 | GU433369 | GU433370 | GU433371 | GU433372 | GU433373 | GU433374 | GU433375 | GU433376 | canine | Guangdong | 2007 |
| A/canine/Guangdong/12/2012 | KF826947 | KF826950 | KF826949 | KF826948 | KF826951 | KF826946 | KF826945 | KF826944 | canine | Guangdong | 2012 |
| A/canine/Guangdong/2/2006 | GU433353 | GU433355 | GU433356 | GU433357 | GU433354 | GU433358 | GU433359 | GU433360 | canine | Guangdong | 2006 |
| A/canine/Guangdong/2/2011 | JX195350 | JX195351 | JX195352 | JX195348 | JX195353 | JX195349 | JX195354 | JX195355 | canine | Guangdong | 2011 |
| A/canine/Guangdong/23/2012 | KF826955 | KF826958 | KF826957 | KF826956 | KF826959 | KF826954 | KF826953 | KF826952 | canine | Guangdong | 2012 |
| A/canine/Guangdong/3/2011 | JX195358 | JX195359 | JX195360 | JX195356 | JX195361 | JX195357 | JX195362 | JX195363 | canine | Guangdong | 2011 |
| A/canine/Heilongjiang/L1/2013 | KF042276 | KF042279 | KF042278 | KF042277 | KF042280 | KF042275 | KF042274 | KF042273 | canine | Heilongjiang | 2013 |
| A/canine/Illinois/077753/2016 | MF173231 | MF173151 | MF173298 | MF173345 | MF173184 | MF173226 | MF173218 | MF173392 | canine | Illinois | 2016 |
| A/canine/Illinois/12191/2015 | KT002536 | KT002539 | KT002538 | KT002537 | KT002540 | KT002535 | KT002534 | KT002533 | canine | Illinois | 2015 |
| A/canine/Illinois/1619144/2015 | KX570967 | KX571055 | KX570973 | KX571038 | KX571044 | KX570990 | KX571041 | KX570978 | canine | Illinois | 2015 |
| A/canine/Illinois/283066/2015 | MF173315 | MF173330 | MF173329 | MF173404 | MF173274 | MF173398 | MF173301 | MF173153 | canine | Illinois | 2015 |
| A/canine/Illinois/328292/2015 | MF173305 | MF173270 | MF173314 | MF173310 | MF173225 | MF173183 | MF173173 | MF173374 | canine | Illinois | 2015 |
| A/canine/Indiana/003018/2016 | MF173371 | MF173227 | MF173113 | MF173158 | MF173288 | MF173340 | MF173166 | MF173221 | canine | Indiana | 2016 |
| A/canine/Indiana/96198/2015 | KX570970 | KX570989 | KX570981 | KX570968 | KX571023 | KX571002 | KX571006 | KX571001 | canine | Indiana | 2015 |
| A/canine/Jiangsu/01/2009 | JN247579 | JN247582 | JN247581 | JN247580 | JN247583 | JN247578 | JN247577 | JN247576 | canine | Jiangsu | 2009 |
| A/canine/Jiangsu/02/2010 | JN247587 | JN247590 | JN247589 | JN247588 | JN247591 | JN247586 | JN247585 | JN247584 | canine | Jiangsu | 2010 |
| A/canine/Jiangsu/03/2010 | JN247595 | JN247598 | JN247597 | JN247596 | JN247599 | JN247594 | JN247593 | JN247592 | canine | Jiangsu | 2010 |
| A/canine/Jiangsu/04/2010 | JN247603 | JN247606 | JN247605 | JN247604 | JN247607 | JN247602 | JN247601 | JN247600 | canine | Jiangsu | 2010 |
| A/canine/Jiangsu/05/2010 | JN247611 | JN247614 | JN247613 | JN247612 | JN247615 | JN247610 | JN247609 | JN247608 | canine | Jiangsu | 2010 |
| A/canine/Jiangsu/06/2010 | JN247619 | JN247622 | JN247621 | JN247620 | JN247623 | JN247618 | JN247617 | JN247616 | canine | Jiangsu | 2010 |
| A/canine/Korea/01/2007 | JX163256 | JX163259 | JX163258 | JX163257 | JX163260 | JX163255 | JX163254 | JX163253 | canine | Korea | 2007 |
| A/canine/Korea/0589318/2015 | KX571053 | KX571045 | KX571019 | KX570994 | KX570993 | KX571012 | KX571025 | KX571005 | canine | Korea | 2015 |
| A/canine/Korea/BD-1/2013 | KR154321 | KR154324 | KR154323 | KR154322 | KR154325 | KR154320 | KR154319 | KR154318 | canine | Korea | 2013 |
| A/canine/Korea/CY009/2010 | KC755907 | KC755921 | KC755915 | KC755911 | KC755925 | KC755903 | KC755901 | KC755899 | canine | Korea | 2010 |
| A/canine/Korea/CY053/2014 | KX509798 | KX509808 | KX509796 | KX509804 | KX509810 | KX509802 | KX509806 | KX509800 | canine | Korea | 2014 |
| A/canine/Korea/DG1/2014 | KR154329 | KR154332 | KR154331 | KR154330 | KR154333 | KR154328 | KR154327 | KR154326 | canine | Korea | 2014 |
| A/canine/Korea/GCVP01/2007 | EU127500 | FJ560885 | EU127501 | FJ560887 | FJ560886 | FJ560888 | FJ560889 | FJ560890 | canine | Korea | 2007 |
| A/canine/Korea/KRIBB01/2011 | JX679525 | JX679528 | JX679527 | JX679526 | JX679529 | JX679524 | JX679523 | JX679522 | canine | Korea | 2011 |
| A/canine/Korea/S1/2012 | KP137810 | KP137813 | KP137812 | KP137811 | KP137814 | KP137809 | KP137808 | KP137807 | canine | Korea | 2012 |
| A/canine/Liaoning/27/2012 | KF042260 | KF042263 | KF042262 | KF042261 | KF042264 | KF042259 | KF042258 | KF042257 | canine | Liaoning | 2012 |
| A/canine/Liaoning/H6/2012 | KF042268 | KF042271 | KF042270 | KF042269 | KF042272 | KF042267 | KF042266 | KF042265 | canine | Liaoning | 2012 |
| A/canine/North Carolina/109904/2015 | KX571008 | KX571020 | KX571007 | KX570964 | KX570971 | KX570965 | KX571003 | KX570975 | canine | North Carolina | 2015 |
| A/canine/South Korea/0173915/2015 | MF173138 | MF173346 | MF173171 | MF173293 | MF173349 | MF173277 | MF173109 | MF173248 | canine | South Korea | 2015 |
| A/canine/Texas/343907/2015 | KX571042 | KX571054 | KX571043 | KX571021 | KX571033 | KX571018 | KX571032 | KX571029 | canine | Texas | 2015 |
| A/canine/Wisconsin/19137/2016 | KX570961 | KX570995 | KX570983 | KX570962 | KX570979 | KX571031 | KX571010 | KX571047 | canine | Wisconsin | 2016 |
| A/canine/Zhejiang/1/2010 | JF714153 | JF714154 | JF714155 | JF714156 | JF714152 | JF714151 | JF714150 | JF714149 | canine | Zhejiang | 2010 |

Table S2. Nucleotide composition, codon composition, ENC, Aroma, Gravy, Axis1 and Axis2 of H3N2 complete coding sequences.

| Strain name | GC_1s_ | GC_2s_ | GC_12s_ | GC_3s_ | A% | C% | G% | U% | AU% | GC% | U_3s_ | C_3s_ | A_3s_ | G_3s_ | ENC | Gravy | Aromo | Axis1 | Axis2 |
| --- | --- | --- | --- | --- | --- | --- | --- | --- | --- | --- | --- | --- | --- | --- | --- | --- | --- | --- | --- |
| A/canine/Zhejiang/1/2010 | 0.499 | 0.410 | 0.455 | 0.418 | 33.380 | 20.780 | 23.450 | 22.380 | 55.760 | 44.240 | 0.310 | 0.275 | 0.417 | 0.269 | 53.170 | -0.478 | 0.069 | 0.019 | 0.004 |
| A/canine/Wisconsin/19137/2016 | 0.495 | 0.409 | 0.452 | 0.406 | 33.890 | 20.730 | 22.930 | 22.450 | 56.340 | 43.660 | 0.311 | 0.274 | 0.432 | 0.254 | 52.850 | -0.482 | 0.069 | -0.030 | 0.001 |
| A/canine/Texas/343907/2015 | 0.495 | 0.409 | 0.452 | 0.407 | 33.870 | 20.740 | 22.990 | 22.410 | 56.280 | 43.720 | 0.310 | 0.275 | 0.431 | 0.255 | 52.880 | -0.484 | 0.069 | -0.031 | 0.001 |
| A/canine/South Korea/0173915/2015 | 0.496 | 0.409 | 0.453 | 0.407 | 33.800 | 20.710 | 23.030 | 22.460 | 56.250 | 43.750 | 0.312 | 0.274 | 0.429 | 0.256 | 52.870 | -0.479 | 0.069 | -0.027 | 0.004 |
| A/canine/North Carolina/109904/2015 | 0.496 | 0.410 | 0.453 | 0.407 | 33.850 | 20.730 | 23.010 | 22.410 | 56.260 | 43.740 | 0.310 | 0.274 | 0.431 | 0.256 | 52.740 | -0.482 | 0.069 | -0.030 | 0.005 |
| A/canine/Liaoning/H6/2012 | 0.499 | 0.410 | 0.454 | 0.411 | 33.580 | 20.680 | 23.310 | 22.430 | 56.010 | 43.990 | 0.310 | 0.273 | 0.425 | 0.262 | 53.060 | -0.466 | 0.068 | 0.008 | -0.023 |
| A/canine/Liaoning/27/2012 | 0.498 | 0.409 | 0.454 | 0.411 | 33.580 | 20.670 | 23.290 | 22.460 | 56.040 | 43.960 | 0.310 | 0.274 | 0.425 | 0.262 | 53.050 | -0.466 | 0.068 | 0.011 | -0.025 |
| A/canine/Korea/S1/2012 | 0.497 | 0.410 | 0.454 | 0.418 | 33.450 | 20.800 | 23.360 | 22.390 | 55.840 | 44.160 | 0.308 | 0.277 | 0.419 | 0.267 | 53.040 | -0.474 | 0.069 | 0.013 | 0.005 |
| A/canine/Korea/KRIBB01/2011 | 0.499 | 0.410 | 0.454 | 0.416 | 33.460 | 20.760 | 23.380 | 22.400 | 55.860 | 44.140 | 0.309 | 0.276 | 0.420 | 0.266 | 53.100 | -0.473 | 0.069 | 0.006 | 0.005 |
| A/canine/Korea/GCVP01/2007 | 0.499 | 0.411 | 0.455 | 0.426 | 33.080 | 20.800 | 23.750 | 22.360 | 55.450 | 44.550 | 0.308 | 0.277 | 0.408 | 0.278 | 53.370 | -0.475 | 0.069 | 0.032 | 0.018 |
| A/canine/Korea/DG1/2014 | 0.497 | 0.410 | 0.453 | 0.415 | 33.620 | 20.820 | 23.200 | 22.360 | 55.980 | 44.020 | 0.307 | 0.277 | 0.424 | 0.262 | 52.970 | -0.476 | 0.069 | 0.003 | -0.002 |
| A/canine/Korea/CY053/2014 | 0.498 | 0.410 | 0.454 | 0.417 | 33.450 | 20.750 | 23.390 | 22.410 | 55.860 | 44.140 | 0.310 | 0.275 | 0.419 | 0.268 | 53.010 | -0.480 | 0.069 | 0.009 | 0.007 |
| A/canine/Korea/CY009/2010 | 0.499 | 0.411 | 0.455 | 0.419 | 33.350 | 20.810 | 23.490 | 22.360 | 55.710 | 44.290 | 0.309 | 0.275 | 0.417 | 0.270 | 53.240 | -0.476 | 0.069 | 0.013 | 0.009 |
| A/canine/Korea/BD-1/2013 | 0.498 | 0.409 | 0.453 | 0.412 | 33.620 | 20.740 | 23.230 | 22.410 | 56.040 | 43.960 | 0.309 | 0.275 | 0.425 | 0.262 | 52.920 | -0.474 | 0.069 | -0.006 | 0.004 |
| A/canine/Korea/0589318/2015 | 0.496 | 0.409 | 0.453 | 0.407 | 33.790 | 20.710 | 23.040 | 22.460 | 56.250 | 43.750 | 0.312 | 0.274 | 0.429 | 0.256 | 52.860 | -0.480 | 0.069 | -0.026 | 0.004 |
| A/canine/Korea/01/2007 | 0.499 | 0.411 | 0.455 | 0.426 | 33.100 | 20.790 | 23.740 | 22.360 | 55.470 | 44.530 | 0.308 | 0.277 | 0.409 | 0.277 | 53.210 | -0.476 | 0.069 | 0.032 | 0.017 |
| A/canine/Jiangsu/06/2010 | 0.498 | 0.411 | 0.454 | 0.415 | 33.500 | 20.760 | 23.360 | 22.380 | 55.880 | 44.120 | 0.310 | 0.274 | 0.420 | 0.265 | 53.100 | -0.475 | 0.068 | 0.012 | 0.003 |
| A/canine/Jiangsu/05/2010 | 0.500 | 0.410 | 0.455 | 0.417 | 33.400 | 20.800 | 23.430 | 22.360 | 55.770 | 44.230 | 0.310 | 0.276 | 0.418 | 0.267 | 53.210 | -0.474 | 0.069 | 0.017 | 0.004 |
| A/canine/Jiangsu/04/2010 | 0.500 | 0.410 | 0.455 | 0.418 | 33.420 | 20.820 | 23.410 | 22.350 | 55.770 | 44.230 | 0.309 | 0.276 | 0.418 | 0.267 | 53.230 | -0.474 | 0.068 | 0.016 | 0.004 |
| A/canine/Jiangsu/03/2010 | 0.500 | 0.410 | 0.455 | 0.418 | 33.380 | 20.860 | 23.410 | 22.350 | 55.730 | 44.270 | 0.309 | 0.277 | 0.417 | 0.268 | 53.240 | -0.475 | 0.068 | 0.017 | 0.007 |
| A/canine/Jiangsu/02/2010 | 0.499 | 0.410 | 0.455 | 0.418 | 33.400 | 20.820 | 23.430 | 22.350 | 55.750 | 44.250 | 0.309 | 0.277 | 0.417 | 0.267 | 53.250 | -0.474 | 0.069 | 0.018 | 0.005 |
| A/canine/Jiangsu/01/2009 | 0.500 | 0.410 | 0.455 | 0.417 | 33.400 | 20.810 | 23.420 | 22.360 | 55.770 | 44.230 | 0.310 | 0.276 | 0.417 | 0.267 | 53.260 | -0.475 | 0.068 | 0.016 | 0.004 |
| A/canine/Indiana/96198/2015 | 0.496 | 0.410 | 0.453 | 0.408 | 33.800 | 20.760 | 23.040 | 22.400 | 56.200 | 43.800 | 0.310 | 0.275 | 0.429 | 0.256 | 52.900 | -0.482 | 0.069 | -0.027 | 0.004 |
| A/canine/Indiana/003018/2016 | 0.495 | 0.410 | 0.453 | 0.407 | 33.860 | 20.740 | 22.990 | 22.410 | 56.280 | 43.720 | 0.310 | 0.275 | 0.431 | 0.254 | 52.880 | -0.482 | 0.069 | -0.031 | 0.001 |
| A/canine/Illinois/1619144/2015 | 0.496 | 0.410 | 0.453 | 0.408 | 33.830 | 20.740 | 23.030 | 22.400 | 56.230 | 43.770 | 0.311 | 0.274 | 0.429 | 0.256 | 52.860 | -0.484 | 0.068 | -0.027 | 0.003 |
| A/canine/Illinois/328292/2015 | 0.495 | 0.409 | 0.452 | 0.407 | 33.880 | 20.720 | 22.980 | 22.410 | 56.300 | 43.700 | 0.310 | 0.275 | 0.431 | 0.255 | 52.900 | -0.485 | 0.069 | -0.032 | 0.002 |
| A/canine/Illinois/283066/2015 | 0.496 | 0.409 | 0.453 | 0.408 | 33.820 | 20.760 | 23.010 | 22.410 | 56.230 | 43.770 | 0.309 | 0.275 | 0.430 | 0.256 | 52.850 | -0.480 | 0.069 | -0.028 | 0.003 |
| A/canine/Illinois/077753/2016 | 0.495 | 0.409 | 0.452 | 0.407 | 33.900 | 20.740 | 22.940 | 22.410 | 56.310 | 43.690 | 0.310 | 0.275 | 0.431 | 0.254 | 52.890 | -0.484 | 0.069 | -0.030 | 0.000 |
| A/canine/Illinois/12191/2015 | 0.496 | 0.409 | 0.453 | 0.408 | 33.810 | 20.770 | 23.020 | 22.400 | 56.210 | 43.790 | 0.310 | 0.275 | 0.430 | 0.256 | 52.880 | -0.482 | 0.069 | -0.027 | 0.003 |
| A/canine/Heilongjiang/L1/2013 | 0.499 | 0.410 | 0.454 | 0.415 | 33.460 | 20.740 | 23.400 | 22.410 | 55.870 | 44.130 | 0.309 | 0.274 | 0.420 | 0.267 | 53.060 | -0.470 | 0.068 | 0.018 | -0.017 |
| A/canine/Guangdong/23/2012 | 0.497 | 0.407 | 0.452 | 0.411 | 33.730 | 20.660 | 23.170 | 22.440 | 56.180 | 43.820 | 0.310 | 0.274 | 0.426 | 0.262 | 53.080 | -0.473 | 0.068 | 0.010 | -0.021 |
| A/canine/Guangdong/12/2012 | 0.498 | 0.407 | 0.452 | 0.412 | 33.700 | 20.680 | 23.200 | 22.420 | 56.120 | 43.880 | 0.311 | 0.274 | 0.425 | 0.263 | 53.080 | -0.475 | 0.068 | 0.012 | -0.018 |
| A/canine/Guangdong/05/2011 | 0.499 | 0.410 | 0.454 | 0.418 | 33.460 | 20.800 | 23.410 | 22.330 | 55.790 | 44.210 | 0.309 | 0.276 | 0.419 | 0.267 | 53.270 | -0.479 | 0.068 | 0.019 | 0.001 |
| A/canine/Guangdong/3/2011 | 0.498 | 0.410 | 0.454 | 0.418 | 33.380 | 20.770 | 23.440 | 22.410 | 55.790 | 44.210 | 0.310 | 0.275 | 0.416 | 0.270 | 53.260 | -0.477 | 0.069 | 0.021 | 0.006 |
| A/canine/Guangdong/2/2011 | 0.499 | 0.410 | 0.454 | 0.419 | 33.350 | 20.790 | 23.470 | 22.390 | 55.740 | 44.260 | 0.309 | 0.276 | 0.416 | 0.270 | 53.400 | -0.472 | 0.069 | 0.022 | 0.004 |
| A/canine/Guangdong/2/2006 | 0.498 | 0.410 | 0.454 | 0.423 | 33.220 | 20.770 | 23.620 | 22.400 | 55.620 | 44.380 | 0.308 | 0.277 | 0.412 | 0.273 | 53.410 | -0.470 | 0.069 | 0.030 | 0.004 |
| A/canine/Guangdong/1/2007 | 0.498 | 0.411 | 0.454 | 0.424 | 33.190 | 20.810 | 23.600 | 22.400 | 55.590 | 44.410 | 0.309 | 0.278 | 0.411 | 0.274 | 53.420 | -0.474 | 0.069 | 0.029 | 0.007 |
| A/canine/Guangdong/1/2006 | 0.500 | 0.412 | 0.456 | 0.423 | 33.160 | 20.810 | 23.660 | 22.370 | 55.530 | 44.470 | 0.309 | 0.276 | 0.411 | 0.275 | 53.220 | -0.475 | 0.068 | 0.027 | 0.015 |
| A/canine/Georgia/104940/2015 | 0.496 | 0.410 | 0.453 | 0.408 | 33.820 | 20.740 | 23.030 | 22.410 | 56.230 | 43.770 | 0.310 | 0.274 | 0.430 | 0.256 | 52.840 | -0.482 | 0.069 | -0.028 | 0.005 |
| A/canine/Georgia/95391/2015 | 0.496 | 0.410 | 0.453 | 0.408 | 33.850 | 20.740 | 23.020 | 22.390 | 56.240 | 43.760 | 0.310 | 0.274 | 0.430 | 0.256 | 52.820 | -0.485 | 0.069 | -0.028 | 0.004 |
| A/canine/Florida/269770/2015 | 0.496 | 0.410 | 0.453 | 0.407 | 33.820 | 20.740 | 23.020 | 22.420 | 56.240 | 43.760 | 0.310 | 0.274 | 0.431 | 0.255 | 52.780 | -0.479 | 0.069 | -0.029 | 0.004 |
| A/canine/China/JLM2/2015 | 0.494 | 0.406 | 0.450 | 0.405 | 33.880 | 20.540 | 22.960 | 22.610 | 56.490 | 43.510 | 0.315 | 0.270 | 0.430 | 0.258 | 52.960 | -0.471 | 0.069 | 0.005 | -0.038 |
| A/canine/China/JLM1/2015 | 0.494 | 0.406 | 0.450 | 0.405 | 33.890 | 20.530 | 22.950 | 22.620 | 56.520 | 43.480 | 0.315 | 0.270 | 0.430 | 0.258 | 52.880 | -0.473 | 0.069 | 0.005 | -0.035 |
| Average | 0.497 | 0.410 | 0.453 | 0.413 | 33.586 | 20.750 | 23.256 | 22.407 | 55.995 | 44.005 | 0.310 | 0.275 | 0.423 | 0.263 | 53.053 | -0.477 | 0.069 | 0.000 | 0.000 |
| SD | 0.002 | 0.001 | 0.001 | 0.006 | 0.250 | 0.065 | 0.242 | 0.056 | 0.283 | 0.283 | 0.002 | 0.002 | 0.007 | 0.007 | 0.192 | 0.005 | 0.000 | 0.023 | 0.012 |

AIVs and human influenza virus

| HA | NA | host | country | year |
| --- | --- | --- | --- | --- |
| CY129062 | CY129064 | avain:American black duck | New Brunswick | 2007 |
| CY129213 | CY129215 | avain:American black duck | New Brunswick | 2007 |
| CY144571 | CY144573 | avain:American black duck | America:North Carolina | 2004 |
| KJ568065 | KJ568067 | avain:American green-winged teal | America:Ohio | 2013 |
| CY079428 | CY079430 | avain:American green-winged teal | America:Wisconsin | 2008 |
| CY079340 | CY079342 | avain:American green-winged teal | America:Wisconsin | 2008 |
| EU301217 | EU301276 | avain:aquatic bird | Korea | 2005 |
| CY167062 | CY167064 | avain:black scoter | America:Wisconsin | 2011 |
| CY187111 | CY187113 | avain:blue winged-teal | America:Wisconsin | 2012 |
| CY187176 | CY187178 | avain:blue winged-teal | America:Wisconsin | 2012 |
| KY644240 | KY644302 | avain:blue-winged teal | Guatemala | 2011 |
| KX960461 | KX960466 | avain:blue-winged teal | Guatemala | 2011 |
| KX960493 | KX960462 | avain:blue-winged teal | Guatemala | 2011 |
| KY644289 | KY644294 | avain:blue-winged teal | Guatemala | 2011 |
| KY644419 | KY644439 | avain:blue-winged teal | Guatemala | 2012 |
| KX960450 | KX960444 | avain:blue-winged teal | Guatemala | 2012 |
| KY644346 | KY644350 | avain:blue-winged teal | Guatemala | 2012 |
| KX960436 | KX960420 | avain:blue-winged teal | Guatemala | 2012 |
| CY133601 | CY133603 | avain:blue-winged teal | America:Iowa | 2010 |
| CY166274 | CY166276 | avain:blue-winged teal | America:Iowa | 2011 |
| KY063884 | KY063886 | avain:blue-winged teal | China:Liaoning | 2014 |
| KY063900 | KY063902 | avain:blue-winged teal | China:Liaoning | 2014 |
| KY063928 | KY063930 | avain:blue-winged teal | China:Liaoning | 2014 |
| KY063936 | KY063938 | avain:blue-winged teal | China:Liaoning | 2014 |
| KY063992 | KY063994 | avain:blue-winged teal | China:Liaoning | 2014 |
| KY284452 | KY284466 | avain:blue-winged teal | America:Missouri | 2015 |
| CY186911 | CY186913 | avain:blue-winged teal | America:Ohio | 2012 |
| KJ567969 | KJ567971 | avain:blue-winged teal | America:Ohio | 2013 |
| CY204040 | CY204042 | avain:blue-winged teal | America:Ohio | 2014 |
| CY017999 | CY018001 | avain:blue-winged teal | America:Ohio | 1999 |
| CY011048 | CY011050 | avain:blue-winged teal | America:Ohio | 2002 |
| CY133101 | CY133103 | avain:blue-winged teal | America:Wisconsin | 2010 |
| CY167102 | CY167104 | avain:blue-winged teal | America:Wisconsin | 2011 |
| CY166314 | CY166316 | avain:blue-winged teal | America:Wisconsin | 2011 |
| CY166501 | CY166503 | avain:blue-winged teal | America:Wisconsin | 2011 |
| CY166509 | CY166511 | avain:blue-winged teal | America:Wisconsin | 2011 |
| KJ567865 | KJ567867 | avain:blue-winged teal | America:Wisconsin | 2013 |
| CY097486 | CY097488 | avain:blue-winged teal | America:Wisconsin | 2009 |
| CY097211 | CY097213 | avain:blue-winged teal | America:Wisconsin | 2009 |
| KY415634 | KY415722 | avain:chicken | Ganzhou | 2016 |
| KY415635 | KY415723 | avain:chicken | Ganzhou | 2016 |
| KT022237 | KT022238 | avain:chicken | Guangxi | 2009 |
| KT022261 | KT022262 | avain:chicken | Guangxi | 2010 |
| KT022277 | KT022278 | avain:chicken | Guangxi | 2012 |
| KT022293 | KT022294 | avain:chicken | Guangxi | 2013 |
| KT022317 | KT022318 | avain:chicken | Guangxi | 2014 |
| EU301222 | EU301285 | avain:chicken | Korea | 2004 |
| EU301224 | EU301287 | avain:chicken | Korea | 2004 |
| EU301231 | EU301294 | avain:chicken | Korea | 2005 |
| EU301232 | EU301295 | avain:chicken | Korea | 2005 |
| EU301234 | EU301297 | avain:chicken | Korea | 2005 |
| EU301236 | EU301299 | avain:chicken | Korea | 2006 |
| EU301238 | EU301301 | avain:chicken | Korea | 2006 |
| AY862607 | AY862639 | avain:chicken | Korea | 2003 |
| EU743195 | EU743197 | avain:chicken | America:NY | 1998 |
| KM222548 | KM222550 | avain:chicken | China:Shanghai | 2013 |
| KT022355 | KT022357 | avain:duck | Anhui | 2014 |
| EPI540287 | EPI540286 | avain:duck | Bangladesh | 2011 |
| EPI540271 | EPI540270 | avain:duck | Bangladesh | 2010 |
| EPI540279 | EPI540278 | avain:duck | Bangladesh | 2011 |
| MG042127 | MG042337 | avain:duck | Bangladesh | 2016 |
| EPI700301 | EPI700302 | avain:duck | Cambodia | 2013 |
| JX175253 | JX175255 | avain:duck | Guangdong | 2011 |
| KT022245 | KT022246 | avain:duck | Guangxi | 2009 |
| KT022253 | KT022254 | avain:duck | Guangxi | 2010 |
| KT022269 | KT022270 | avain:duck | Guangxi | 2012 |
| KT022301 | KT022302 | avain:duck | Guangxi | 2013 |
| KU158891 | KU158905 | avain:duck | Hebei | 2011 |
| KU158892 | KU158906 | avain:duck | Hebei | 2011 |
| KU158893 | KU158907 | avain:duck | Hebei | 2011 |
| FJ432762 | FJ432764 | avain:duck | Italy | 2006 |
| MG021165 | MG021167 | avain:duck | China:Jiangshu | 2016 |
| KC261669 | KC261671 | avain:duck | China:Jiangshu | 2004 |
| JN087096 | JN087098 | avain:duck | Korea | 2004 |
| EU301221 | EU301284 | avain:duck | Korea | 2004 |
| EU301223 | EU301286 | avain:duck | Korea | 2004 |
| EU301225 | EU301288 | avain:duck | Korea | 2004 |
| EU301226 | EU301289 | avain:duck | Korea | 2005 |
| EU301227 | EU301290 | avain:duck | Korea | 2005 |
| EU301230 | EU301293 | avain:duck | Korea | 2005 |
| EU301233 | EU301296 | avain:duck | Korea | 2005 |
| EU301235 | EU301298 | avain:duck | Korea | 2006 |
| EU301237 | EU301300 | avain:duck | Korea | 2006 |
| EU301239 | EU301302 | avain:duck | Korea | 2006 |
| EU301240 | EU301303 | avain:duck | Korea | 2006 |
| AB701298 | AB701299 | avain:duck | Mongolia | 2011 |
| EU742652 | EU742654 | avain:duck | America:NY | 1978 |
| LC053492 | LC053494 | avain:duck | Quang Ninh | 2014 |
| LC053468 | LC053470 | avain:duck | Quang Ninh | 2013 |
| KU158894 | KU158908 | avain:duck | China:Shanghai | 2009 |
| JX308801 | JX286593 | avain:duck | China:Shanghai | 2009 |
| KM222556 | KM222558 | avain:duck | China:Shanghai | 2013 |
| KM222564 | KM222566 | avain:duck | China:Shanghai | 2013 |
| KM222572 | KM222574 | avain:duck | China:Shanghai | 2013 |
| FJ802401 | FJ802403 | avain:duck | Thailand | 2008 |
| LC041332 | LC041333 | avain:duck | Vietnam | 2014 |
| LC028080 | LC028082 | avain:duck | Vietnam | 2011 |
| LC028160 | LC028162 | avain:duck | Vietnam | 2013 |
| AB545597 | AB545598 | avain:duck | Vietnam | 2009 |
| KJ439849 | KJ439871 | avain:duck | China:Zhejiang | 2013 |
| KJ439850 | KJ439872 | avain:duck | China:Zhejiang | 2013 |
| KJ439847 | KJ439869 | avain:duck | China:Zhejiang | 2013 |
| KJ439848 | KJ439870 | avain:duck | China:Zhejiang | 2013 |
| CY079348 | CY079350 | avain:gadwall | America:Wisconsin | 2008 |
| KT022309 | KT022310 | avain:goose | Guangxi | 2013 |
| JX080755 | JX081146 | avain:greater white-fronted goose | America:Alaska | 2006 |
| CY204024 | CY204026 | avain:green-winged teal | America:Ohio | 2014 |
| KY561045 | KY561119 | avain:green-winged teal | America:Ohio | 2015 |
| GU186484 | EU743204 | avain:guinea fowl | America:NJ | 1998 |
| HM193564 | HM193630 | avain:mallard | America:Alaska | 2006 |
| HM193561 | HM193631 | avain:mallard | America:Alaska | 2006 |
| HM193563 | HM193632 | avain:mallard | America:Alaska | 2006 |
| HM193557 | HM193633 | avain:mallard | America:Alaska | 2006 |
| EPI859643 | EPI859645 | avain:mallard | Germany | 2004 |
| EPI185346 | EPI185340 | avain:mallard | Germany | 2004 |
| CY078499 | CY078501 | avain:mallard | Interior Alaska | 2006 |
| CY078507 | CY078509 | avain:mallard | Interior Alaska | 2006 |
| CY098262 | CY098264 | avain:mallard | China:Jiangxi | 2010 |
| CY203557 | CY203559 | avain:mallard | America:Maryland | 2005 |
| CY203565 | CY203567 | avain:mallard | America:Maryland | 2005 |
| CY203573 | CY203575 | avain:mallard | America:Maryland | 2005 |
| CY203581 | CY203583 | avain:mallard | America:Maryland | 2005 |
| CY203589 | CY203591 | avain:mallard | America:Maryland | 2005 |
| CY203597 | CY203599 | avain:mallard | America:Maryland | 2005 |
| CY203164 | CY203166 | avain:mallard | America:Maryland | 2009 |
| CY203172 | CY203174 | avain:mallard | America:Maryland | 2009 |
| CY203261 | CY203263 | avain:mallard | America:Maryland | 2010 |
| CY203285 | CY203287 | avain:mallard | America:Maryland | 2010 |
| CY203325 | CY203327 | avain:mallard | America:Maryland | 2010 |
| CY203341 | CY203343 | avain:mallard | America:Maryland | 2010 |
| CY021453 | CY021455 | avain:mallard | America:Maryland | 2005 |
| CY021277 | CY021279 | avain:mallard | America:Maryland | 2005 |
| CY021261 | CY021263 | avain:mallard | America:Maryland | 2005 |
| CY021269 | CY021271 | avain:mallard | America:Maryland | 2005 |
| CY021461 | CY021463 | avain:mallard | America:Maryland | 2005 |
| CY022741 | CY022743 | avain:mallard | America:Maryland | 2005 |
| CY132125 | CY132127 | avain:mallard | America:Michigan | 2010 |
| CY139977 | CY139979 | avain:mallard | America:Minnesota | 1999 |
| CY140911 | CY140913 | avain:mallard | America:Minnesota | 2009 |
| CY140483 | CY140485 | avain:mallard | America:Minnesota | 2008 |
| CY140583 | CY140585 | avain:mallard | America:Minnesota | 2008 |
| EU743501 | EU743503 | avain:mallard | America:MN | 1999 |
| EU743508 | EU743510 | avain:mallard | America:MN | 1999 |
| EU743495 | EU743496 | avain:mallard | America:MN | 1999 |
| CY043816 | CY043818 | avain:mallard | Netherlands | 2007 |
| CY145751 | CY145753 | avain:mallard | America:New Jersey | 2008 |
| CY145767 | CY145769 | avain:mallard | America:New Jersey | 2008 |
| CY077537 | CY077539 | avain:mallard | New Zealand | 2004 |
| CY132093 | CY132095 | avain:mallard | America:Ohio | 2010 |
| CY132101 | CY132103 | avain:mallard | America:Ohio | 2010 |
| CY132109 | CY132111 | avain:mallard | America:Ohio | 2010 |
| CY132301 | CY132303 | avain:mallard | America:Ohio | 2011 |
| CY204113 | CY204115 | avain:mallard | America:Ohio | 2014 |
| CY204121 | CY204123 | avain:mallard | America:Ohio | 2014 |
| CY204129 | CY204131 | avain:mallard | America:Ohio | 2014 |
| CY204145 | CY204147 | avain:mallard | America:Ohio | 2014 |
| CY204177 | CY204179 | avain:mallard | America:Ohio | 2014 |
| CY204215 | CY204217 | avain:mallard | America:Ohio | 2014 |
| CY204232 | CY204234 | avain:mallard | America:Ohio | 2014 |
| CY204297 | CY204299 | avain:mallard | America:Ohio | 2014 |
| CY204314 | CY204316 | avain:mallard | America:Ohio | 2014 |
| KY583991 | KY584037 | avain:mallard | America:Ohio | 2015 |
| KY583932 | KY583866 | avain:mallard | America:Ohio | 2015 |
| CY017411 | CY017413 | avain:mallard | America:Ohio | 1988 |
| CY020717 | CY020719 | avain:mallard | America:Ohio | 1986 |
| CY047592 | CY047594 | avain:mallard | Quebec | 2006 |
| CY047600 | CY047602 | avain:mallard | Quebec | 2006 |
| CY047632 | CY047634 | avain:mallard | Quebec | 2006 |
| CY047672 | CY047674 | avain:mallard | Quebec | 2006 |
| CY047656 | CY047658 | avain:mallard | Quebec | 2006 |
| CY132933 | CY132935 | avain:mallard | America:Wisconsin | 2010 |
| CY133193 | CY133195 | avain:mallard | America:Wisconsin | 2010 |
| CY166330 | CY166332 | avain:mallard | America:Wisconsin | 2011 |
| CY166338 | CY166340 | avain:mallard | America:Wisconsin | 2011 |
| CY166354 | CY166356 | avain:mallard | America:Wisconsin | 2011 |
| CY166840 | CY166842 | avain:mallard | America:Wisconsin | 2011 |
| CY166848 | CY166850 | avain:mallard | America:Wisconsin | 2011 |
| CY166856 | CY166858 | avain:mallard | America:Wisconsin | 2011 |
| CY166864 | CY166866 | avain:mallard | America:Wisconsin | 2011 |
| CY166880 | CY166882 | avain:mallard | America:Wisconsin | 2011 |
| CY166905 | CY166907 | avain:mallard | America:Wisconsin | 2011 |
| CY166517 | CY166519 | avain:mallard | America:Wisconsin | 2011 |
| CY166597 | CY166599 | avain:mallard | America:Wisconsin | 2011 |
| CY166435 | CY166437 | avain:mallard | America:Wisconsin | 2011 |
| CY166460 | CY166462 | avain:mallard | America:Wisconsin | 2011 |
| CY166419 | CY166421 | avain:mallard | America:Wisconsin | 2011 |
| CY096986 | CY096988 | avain:mallard | America:Wisconsin | 2009 |
| CY097326 | CY097328 | avain:mallard | America:Wisconsin | 2009 |
| CY097342 | CY097344 | avain:mallard | America:Wisconsin | 2009 |
| MF145754 | MF147222 | avain:mallard duck | Netherlands | 2011 |
| MF145991 | MF146628 | avain:mallard duck | Netherlands | 2012 |
| MF146106 | MF146335 | avain:mallard duck | Netherlands | 2010 |
| MF146864 | MF146565 | avain:mallard duck | Netherlands | 2008 |
| CY024810 | CY024812 | avain:mallard.Maryland | America:Maryland | 2005 |
| KP714429 | KP714430 | avain:mule duck | Bulgaria | 2009 |
| LC028104 | LC028106 | avain:muscovy duck | Vietnam | 2012 |
| AB746482 | AB746484 | avain:muscovy duck | Vietnam | 2011 |
| AB781669 | AB781671 | avain:muscovy duck | Vietnam | 2012 |
| LC028112 | LC028114 | avain:muscovy duck | Vietnam | 2012 |
| LC053476 | LC053478 | avain:muscovy duck | Vietnam | 2014 |
| CY045319 | CY045321 | avain:northern pintail | Saskatchewan | 2007 |
| CY041863 | CY041865 | avain:northern pintail | America:South Dakota | 2007 |
| CY097494 | CY097496 | avain:northern pintail | America:Wisconsin | 2009 |
| KT022285 | KT022286 | avain:pigeon | Guangxi | 2012 |
| CY005939 | CY004621 | avain:pintail duck | ALB | 1979 |
| CY005935 | CY004578 | avain:pintail duck | ALB | 1976 |
| HQ825174 | HQ825176 | avain:quail | QC | 2008 |
| CY102100 | CY102102 | avain:red knot | Delaware Bay | 1994 |
| CY145493 | CY145495 | avain:ruddy turnstone | America:Delaware | 2008 |
| CY101258 | CY101260 | avain:ruddy turnstone | Delaware Bay | 1999 |
| CY102404 | CY102406 | avain:ruddy turnstone | Delaware Bay | 1999 |
| CY102003 | CY102005 | avain:ruddy turnstone | Delaware Bay | 1994 |
| CY101890 | CY101892 | avain:ruddy turnstone | Delaware Bay | 1994 |
| CY145152 | CY145154 | avain:ruddy turnstone | America:New Jersey | 2008 |
| CY145623 | CY145625 | avain:ruddy turnstone | America:New Jersey | 2008 |
| CY145185 | CY145187 | avain:ruddy turnstone | America:New Jersey | 2008 |
| CY145193 | CY145195 | avain:ruddy turnstone | America:New Jersey | 2008 |
| CY145217 | CY145219 | avain:ruddy turnstone | America:New Jersey | 2008 |
| CY145257 | CY145259 | avain:ruddy turnstone | America:New Jersey | 2008 |
| CY145671 | CY145673 | avain:ruddy turnstone | America:New Jersey | 2008 |
| CY102364 | CY102366 | avain:shorebird | Delaware Bay | 1999 |
| CY102380 | CY102382 | avain:shorebird | Delaware Bay | 1999 |
| CY102396 | CY102398 | avain:shorebird | Delaware Bay | 1999 |
| CY102439 | CY102441 | avain:shorebird | Delaware Bay | 1999 |
| CY102447 | CY102449 | avain:shorebird | Delaware Bay | 1999 |
| CY102455 | CY102457 | avain:shorebird | Delaware Bay | 1999 |
| CY102476 | CY102478 | avain:shorebird | Delaware Bay | 1999 |
| CY102484 | CY102486 | avain:shorebird | Delaware Bay | 1999 |
| CY102492 | CY102494 | avain:shorebird | Delaware Bay | 1999 |
| CY102500 | CY102502 | avain:shorebird | Delaware Bay | 1999 |
| CY102508 | CY102510 | avain:shorebird | Delaware Bay | 1999 |
| CY102516 | CY102518 | avain:shorebird | Delaware Bay | 1999 |
| CY102574 | CY102576 | avain:shorebird | Delaware Bay | 1999 |
| CY101856 | CY101858 | avain:shorebird | Delaware Bay | 1994 |
| CY103459 | CY103461 | avain:shorebird | Delaware Bay | 2008 |
| CY101872 | CY101874 | avain:shorebird | Delaware Bay | 1994 |
| CY102348 | CY102350 | avain:shorebird | Delaware Bay | 1999 |
| CY102356 | CY102358 | avain:shorebird | Delaware Bay | 1999 |
| HQ825166 | HQ825168 | avain:turkey | BC | 2005 |
| GU052275 | GU052277 | avain:Turkey | England | 1969 |
| EF551045 | EF551047 | avain:turkey | America:Illinois | 2004 |
| EU697204 | EU697206 | avain:turkey | America:Minnesota | 2005 |
| EU743210 | EU743212 | avain:turkey | America:MN | 2005 |
| EU735826 | EU735828 | avain:turkey | NC | 2005 |
| EU697209 | EU697211 | avain:turkey | America:North Carolina | 2005 |
| KX859344 | KX859385 | avain:turkey | America:OH | 2004 |
| KX859403 | KX859337 | avain:turkey | America:OH | 2004 |
| KX859435 | KX859384 | avain:turkey | America:OH | 2004 |
| KX859444 | KX859402 | avain:turkey | America:OH | 2004 |
| KX859365 | KX859412 | avain:turkey | America:OH | 2004 |
| KX859391 | KX859333 | avain:turkey | America:OH | 2004 |
| EU735818 | EU735820 | avain:turkey | America:OH | 2004 |
| DQ335771 | DQ335773 | avain:turkey | America:Ohio | 2004 |
| DQ470002 | DQ470000 | avain:turkey | Ontario | 2005 |
| JN706700 | JN706702 | avain:turkey | Ontario | 2011 |
| JN683637 | JN683639 | avain:turkey | Ontario | 2011 |
| JN683629 | JN683631 | avain:turkey | Ontario | 2011 |
| AB557631 | AB557632 | avain:white-backed munia | Hong Kong | 2009 |
| EPI185349 | EPI185342 | avain:wild duck | Germany | 2006 |
| MG386676 | MG386678 | canine | China | 2015 |
| MG386684 | MG386686 | canine | China | 2015 |
| MF173382 | MF173401 | canine | America:Florida | 2015 |
| KX571026 | KX570966 | canine | America:Georgia | 2015 |
| KX571027 | KX570998 | canine | America:Georgia | 2015 |
| JX414244 | JX414246 | canine | Guangdong | 2011 |
| GU433345 | GU433347 | canine | Guangdong | 2006 |
| GU433369 | GU433371 | canine | Guangdong | 2007 |
| KF826947 | KF826949 | canine | Guangdong | 2012 |
| GU433353 | GU433356 | canine | Guangdong | 2006 |
| JX195350 | JX195352 | canine | Guangdong | 2011 |
| KF826955 | KF826957 | canine | Guangdong | 2012 |
| JX195358 | JX195360 | canine | Guangdong | 2011 |
| KF042276 | KF042278 | canine | Heilongjiang | 2013 |
| MF173231 | MF173298 | canine | America:Illinois | 2016 |
| KT002536 | KT002538 | canine | America:Illinois | 2015 |
| KX570967 | KX570973 | canine | America:Illinois | 2015 |
| MF173315 | MF173329 | canine | America:Illinois | 2015 |
| MF173305 | MF173314 | canine | America:Illinois | 2015 |
| MF173371 | MF173113 | canine | America:Indiana | 2016 |
| KX570970 | KX570981 | canine | America:Indiana | 2015 |
| JN247579 | JN247581 | canine | China:Jiangshu | 2009 |
| JN247587 | JN247589 | canine | China:Jiangshu | 2010 |
| JN247595 | JN247597 | canine | China:Jiangshu | 2010 |
| JN247603 | JN247605 | canine | China:Jiangshu | 2010 |
| JN247611 | JN247613 | canine | China:Jiangshu | 2010 |
| JN247619 | JN247621 | canine | China:Jiangshu | 2010 |
| JX163256 | JX163258 | canine | Korea | 2007 |
| KX571053 | KX571019 | canine | Korea | 2015 |
| KR154321 | KR154323 | canine | Korea | 2013 |
| KC755907 | KC755915 | canine | Korea | 2010 |
| KX509798 | KX509796 | canine | Korea | 2014 |
| KR154329 | KR154331 | canine | Korea | 2014 |
| EU127500 | EU127501 | canine | Korea | 2007 |
| JX679525 | JX679527 | canine | Korea | 2011 |
| KP137810 | KP137812 | canine | Korea | 2012 |
| KF042260 | KF042262 | canine | China:Liaoning | 2012 |
| KF042268 | KF042270 | canine | China:Liaoning | 2012 |
| KX571008 | KX571007 | canine | America:North Carolina | 2015 |
| MF173138 | MF173171 | canine | South Korea | 2015 |
| KX571042 | KX571043 | canine | America:Texas | 2015 |
| KX570961 | KX570983 | canine | America:Wisconsin | 2016 |
| JF714153 | JF714155 | canine | China:Zhejiang | 2010 |
| CY093359 | CY093361 | human | America:Arizona | 2009 |
| CY114293 | CY114295 | human | Auckland | 1997 |
| CY023026 | CY023028 | human | Auckland | 2000 |
| CY080555 | CY080557 | human | Australia | 2009 |
| CY091285 | CY091287 | human | Australia | 2003 |
| CY091333 | CY091335 | human | Australia | 2005 |
| CY104548 | CY104550 | human | BacNinh | 2007 |
| CY114461 | CY114463 | human | Beijing | 1989 |
| KM821281 | CY116574 | human | Bilthoven | 1976 |
| CY044572 | CY044574 | human | Boston | 2008 |
| CY044852 | CY044854 | human | Boston | 2008 |
| KM978061 | CY116578 | human | Brisbane | 2007 |
| KM978059 | KM978072 | human | BRISBANE | 2005 |
| CY114373 | CY114375 | human | America:California | 2004 |
| CY091173 | CY091175 | human | America:California | 2003 |
| CY091509 | CY091511 | human | America:California | 2005 |
| CY091525 | CY091527 | human | America:California | 2005 |
| CY091549 | CY091551 | human | America:California | 2005 |
| CY026787 | CY026789 | human | America:California | 2007 |
| CY068177 | CY068179 | human | America:California | 2009 |
| CY067197 | CY067199 | human | America:California | 2009 |
| CY068281 | CY068283 | human | America:California | 2009 |
| CY068838 | CY068840 | human | America:California | 2009 |
| CY008556 | CY008558 | human | Canterbury | 2005 |
| CY007371 | CY007373 | human | Canterbury | 2004 |
| CY007859 | CY007861 | human | Canterbury | 2002 |
| CY039964 | CY096845 | human | Canterbury | 2004 |
| CY007523 | CY007525 | human | Canterbury | 2004 |
| CY008043 | CY008045 | human | Canterbury | 2005 |
| CY008636 | CY008638 | human | Canterbury | 2005 |
| CY008652 | CY008654 | human | Canterbury | 2005 |
| CY008252 | CY008254 | human | Canterbury | 2004 |
| CY007571 | CY007573 | human | Canterbury | 2004 |
| CY007891 | CY007893 | human | Canterbury | 2002 |
| CY006971 | CY006973 | human | Canterbury | 2003 |
| CY007075 | CY007077 | human | Canterbury | 2003 |
| CY008548 | CY008550 | human | Canterbury | 2003 |
| CY007243 | CY007245 | human | Canterbury | 2003 |
| CY019981 | CY019983 | human | Canterbury | 2000 |
| CY008804 | CY008806 | human | Canterbury | 2000 |
| CY113357 | CY113359 | human | America:Colorado | 1986 |
| CY027547 | CY027549 | human | America:Colorado | 2007 |
| CY025349 | CY025351 | human | America:Colorado | 2007 |
| EU516019 | EU199421 | human | America:Delaware | 2007 |
| EU103700 | EU103877 | human | Denmark | 2005 |
| EU103663 | EU103849 | human | Denmark | 2004 |
| EU103695 | EU103873 | human | Denmark | 2006 |
| AY531056 | AY531028 | human | Denmark | 2003 |
| EU103724 | EU103901 | human | Denmark | 2003 |
| EU103756 | EU103929 | human | Denmark | 2005 |
| EU103674 | EU103854 | human | Denmark | 2005 |
| EU103634 | EU103828 | human | Denmark | 2002 |
| EU103800 | EU103960 | human | Denmark | 2006 |
| AY531044 | AY531011 | human | Denmark | 2003 |
| EU103685 | EU103863 | human | Denmark | 2003 |
| CY011400 | CY011402 | human | Dunedin | 2002 |
| CY011656 | CY011658 | human | Dunedin | 2002 |
| CY013375 | CY013377 | human | Dunedin | 2000 |
| CY012008 | CY012010 | human | Dunedin | 2002 |
| CY088182 | CY088184 | human | England | 2003 |
| CY088206 | CY088208 | human | England | 2003 |
| CY088366 | CY088368 | human | England | 2003 |
| CY088214 | CY088216 | human | England | 2003 |
| CY025341 | CY025343 | human | America:Florida | 2007 |
| CY093255 | CY093257 | human | America:Florida | 2009 |
| CY088483 | CY112935 | human | Fujian | 2002 |
| CY105886 | CY105888 | human | HaNoi | 2005 |
| CY104076 | CY104078 | human | HaNoi | 2003 |
| CY105486 | CY105488 | human | HaNoi | 2007 |
| CY105510 | CY105512 | human | HaNoi | 2007 |
| CY105318 | CY105320 | human | HaNoi | 2004 |
| CY104396 | CY104398 | human | HaNoi | 2007 |
| CY105582 | CY105584 | human | HaNoi | 2007 |
| CY105638 | CY105640 | human | HaNoi | 2007 |
| CY105710 | CY105712 | human | HaNoi | 2007 |
| CY105790 | CY105792 | human | HaNoi | 2007 |
| KF805656 | KF805654 | human | Helsinki | 2013 |
| KF805696 | KF805694 | human | Helsinki | 2013 |
| AF382323 | AF386761 | human | Hong Kong | 1999 |
| CY003712 | CY003714 | human | Hong Kong | 1992 |
| CY112773 | CY112775 | human | Hong Kong | 1995 |
| CY006323 | CY006325 | human | Hong Kong | 1984 |
| CY003544 | CY003546 | human | Hong Kong | 1987 |
| EU856848 | EU857129 | human | Hong Kong | 1999 |
| EU856864 | EU857145 | human | Hong Kong | 2003 |
| EU856868 | EU857149 | human | Hong Kong | 1999 |
| EU856897 | EU857178 | human | Hong Kong | 2000 |
| EU856934 | EU857215 | human | Hong Kong | 2002 |
| EU856935 | EU857216 | human | Hong Kong | 2002 |
| EU856989 | EU857270 | human | Hong Kong | 2002 |
| EU857024 | EU857305 | human | Hong Kong | 2004 |
| EU857033 | EU857314 | human | Hong Kong | 2001 |
| EU857050 | EU857331 | human | Hong Kong | 2002 |
| EU857079 | EU857360 | human | Hong Kong | 2005 |
| EU857093 | EU857374 | human | Hong Kong | 2005 |
| CY039055 | CY039057 | human | Hong Kong | 2005 |
| CY043752 | CY043754 | human | Hong Kong | 2005 |
| CY039159 | CY039161 | human | Hong Kong | 2004 |
| CY039191 | CY039193 | human | Hong Kong | 2005 |
| CY039511 | CY039513 | human | Hong Kong | 2005 |
| CY039223 | CY039225 | human | Hong Kong | 2005 |
| CY039239 | CY039241 | human | Hong Kong | 2005 |
| CY039247 | CY039249 | human | Hong Kong | 2005 |
| CY038735 | CY038737 | human | Hong Kong | 2005 |
| EU516331 | EU516332 | human | HongKong | 2004 |
| CY113709 | CY113711 | human | Houston | 1992 |
| CY105558 | CY105560 | human | HungYen | 2007 |
| CY026027 | CY026029 | human | America:Illinois | 2007 |
| JQ070768 | JQ290171 | human | America:Iowa | 2011 |
| CY026883 | CY026885 | human | America:Kentucky | 2007 |
| CY037879 | CY037881 | human | America:Kentucky | 2008 |
| CY110759 | CY110761 | human | China:Kunming | 2005 |
| CY114277 | CY114279 | human | Lyon | 1996 |
| CY113653 | CY113655 | human | Madrid | 1991 |
| CY117698 | CY117700 | human | Malaysia | 1996 |
| CY118450 | CY118452 | human | Malaysia | 1996 |
| CY118458 | CY118460 | human | Malaysia | 1996 |
| CY118634 | CY118636 | human | Malaysia | 1999 |
| CY118490 | CY118492 | human | Malaysia | 1999 |
| CY118506 | CY118508 | human | Malaysia | 1996 |
| CY118051 | CY118053 | human | Malaysia | 2007 |
| CY118151 | CY118153 | human | Malaysia | 2008 |
| CY117960 | CY117962 | human | Malaysia | 2003 |
| CY117971 | CY117973 | human | Malaysia | 2003 |
| CY118674 | CY118676 | human | Malaysia | 2003 |
| CY118738 | CY118740 | human | Malaysia | 2004 |
| CY118794 | CY118796 | human | Malaysia | 2004 |
| CY118802 | CY118804 | human | Malaysia | 2004 |
| CY118818 | CY118820 | human | Malaysia | 2004 |
| CY091453 | CY091455 | human | Malaysia | 2004 |
| CY038551 | CY038553 | human | Managua | 2007 |
| CY032493 | CY032495 | human | Managua | 2007 |
| CY008716 | CY008718 | human | Memphis | 1986 |
| AY271794 | AY271795 | human | Memphis | 1998 |
| CY008452 | CY008454 | human | Memphis | 1985 |
| CY113509 | CY113511 | human | Memphis | 1990 |
| CY147627 | CY147629 | human | Mexico | 2008 |
| KJ855387 | KJ855389 | human | Mexico | 2003 |
| EU516053 | EU516175 | human | America:Michigan | 2007 |
| DQ089638 | DQ090708 | human | Moscow | 2003 |
| KM061040 | KJ511847 | human | Mumbai | 2009 |
| KM061034 | KJ511841 | human | Mumbai | 2009 |
| CY112789 | CY112791 | human | Nanchang | 1995 |
| CY006331 | CY006333 | human | Nanchang | 1994 |
| CY009124 | CY009126 | human | Nelson Marlborough | 2000 |
| CY114045 | CY114047 | human | Netherlands | 1993 |
| CY112597 | CY112599 | human | Netherlands | 1993 |
| CY114333 | CY114335 | human | Netherlands | 2001 |
| CY077800 | CY112607 | human | Netherlands | 1993 |
| CY113269 | CY113271 | human | Netherlands | 1980 |
| KM821282 | CY114439 | human | Netherlands | 1982 |
| CY113293 | CY113295 | human | Netherlands | 1982 |
| CY114365 | CY114367 | human | Netherlands | 2003 |
| CY114069 | CY114071 | human | Netherlands | 1993 |
| CY112621 | CY112623 | human | Netherlands | 1993 |
| CY114085 | CY114087 | human | Netherlands | 1993 |
| CY112629 | CY116594 | human | Netherlands | 1993 |
| CY114389 | CY114391 | human | Netherlands | 2006 |
| CY112997 | CY112999 | human | Netherlands | 2005 |
| CY113749 | CY113751 | human | Netherlands | 1991 |
| CY016116 | CY016118 | human | New South Wales | 2000 |
| CY172191 | CY172193 | human | America:New York | 2005 |
| CY000417 | CY000419 | human | America:New York | 2002 |
| CY172415 | CY172417 | human | America:New York | 2006 |
| CY172735 | CY172737 | human | America:New York | 2007 |
| CY172815 | CY172817 | human | America:New York | 2007 |
| CY172879 | CY172881 | human | America:New York | 2007 |
| CY000553 | CY000555 | human | America:New York | 2002 |
| CY001944 | CY001946 | human | America:New York | 2002 |
| CY000584 | CY000578 | human | America:New York | 2001 |
| CY000457 | CY000459 | human | America:New York | 1999 |
| CY002312 | CY002314 | human | America:New York | 1999 |
| CY000657 | CY000659 | human | America:New York | 2000 |
| CY000705 | CY000707 | human | America:New York | 2000 |
| CY000729 | CY000731 | human | America:New York | 1999 |
| CY001357 | CY001359 | human | America:New York | 1999 |
| CY001528 | CY001530 | human | America:New York | 1999 |
| CY001005 | CY001007 | human | America:New York | 1999 |
| CY000473 | CY000475 | human | America:New York | 2003 |
| CY002768 | CY002770 | human | America:New York | 2004 |
| CY001405 | CY001407 | human | America:New York | 2003 |
| CY000769 | CY000771 | human | America:New York | 2003 |
| CY001600 | CY001602 | human | America:New York | 1999 |
| CY001608 | CY001610 | human | America:New York | 1999 |
| CY001768 | CY001770 | human | America:New York | 1999 |
| CY001664 | CY001666 | human | America:New York | 1999 |
| FJ686942 | FJ686941 | human | America:New York | 2008 |
| CY001792 | CY001794 | human | America:New York | 1999 |
| CY058748 | CY058750 | human | America:New York | 2009 |
| CY050500 | CY050502 | human | America:New York | 2009 |
| CY001840 | CY001842 | human | America:New York | 1999 |
| CY000025 | CY000027 | human | America:New York | 2003 |
| CY001984 | CY001986 | human | America:New York | 1999 |
| CY006115 | CY006117 | human | America:New York | 2004 |
| CY002424 | CY002426 | human | America:New York | 2004 |
| CY002008 | CY002010 | human | America:New York | 2005 |
| CY006435 | CY006437 | human | America:New York | 2004 |
| CY050828 | CY050830 | human | America:New York | 2009 |
| CY002248 | CY002250 | human | America:New York | 2004 |
| CY002736 | CY002738 | human | America:New York | 2005 |
| CY000145 | CY000147 | human | America:New York | 2003 |
| CY003200 | CY003202 | human | America:New York | 2002 |
| CY058804 | CY058806 | human | America:New York | 2009 |
| CY003248 | CY003250 | human | America:New York | 2000 |
| CY003256 | CY003258 | human | America:New York | 2000 |
| CY003264 | CY003266 | human | America:New York | 2000 |
| CY003456 | CY003458 | human | America:New York | 2000 |
| CY003825 | CY003827 | human | America:New York | 2000 |
| CY003608 | CY003610 | human | America:New York | 1999 |
| CY003616 | CY003618 | human | America:New York | 1999 |
| CY008892 | CY008894 | human | America:New York | 2003 |
| CY008916 | CY008918 | human | America:New York | 2003 |
| CY003680 | CY003682 | human | America:New York | 2003 |
| CY006443 | CY006445 | human | America:New York | 1997 |
| CY006235 | CY006237 | human | America:New York | 1997 |
| CY001064 | CY001066 | human | America:New York | 2003 |
| CY006475 | CY006477 | human | America:New York | 1998 |
| CY006483 | CY006485 | human | America:New York | 1998 |
| CY008948 | CY008950 | human | America:New York | 1998 |
| CY006595 | CY006597 | human | America:New York | 1998 |
| CY121125 | CY116596 | human | America:New York | 2004 |
| CY011256 | CY011258 | human | America:New York | 1996 |
| CY009644 | CY009646 | human | America:New York | 1997 |
| CY009484 | CY009486 | human | America:New York | 1997 |
| CY000957 | CY000959 | human | America:New York | 2003 |
| CY009748 | CY009750 | human | America:New York | 1997 |
| CY010684 | CY010686 | human | America:New York | 1996 |
| CY011456 | CY011458 | human | America:New York | 1996 |
| CY011808 | CY011810 | human | America:New York | 1995 |
| CY001221 | CY001223 | human | America:New York | 2003 |
| CY012448 | CY012450 | human | America:New York | 1994 |
| CY012464 | CY012466 | human | America:New York | 1994 |
| CY012472 | CY012474 | human | America:New York | 1995 |
| CY011320 | CY011322 | human | America:New York | 1995 |
| CY011344 | CY011346 | human | America:New York | 1995 |
| CY011880 | CY011882 | human | America:New York | 1994 |
| CY012992 | CY012994 | human | America:New York | 1994 |
| CY013613 | CY013615 | human | America:New York | 1993 |
| CY013685 | CY013687 | human | America:New York | 1993 |
| CY001045 | CY001047 | human | America:New York | 2003 |
| CY013781 | CY013783 | human | America:New York | 1993 |
| CY001261 | CY001263 | human | America:New York | 2002 |
| CY019165 | CY019143 | human | America:New York | 2004 |
| CY025843 | CY025845 | human | America:New York | 2007 |
| CY037743 | CY037745 | human | America:New York | 2008 |
| CY188601 | CY188603 | human | Nicaragua | 2007 |
| CY027075 | CY027077 | human | America:Oregon | 2007 |
| CY113805 | CY113807 | human | Paris | 1991 |
| CY113861 | CY113863 | human | Paris | 1992 |
| CY035062 | CY035064 | human | America:Pennsylvania | 2008 |
| CY163112 | CY163114 | human | Peru | 2011 |
| CY017499 | CY017501 | human | Queensland | 2001 |
| CY019021 | CY019023 | human | Queensland | 2005 |
| CY017611 | CY017613 | human | Queensland | 2005 |
| KY925368 | KY925575 | human | Sapucaia do Sul | 2011 |
| CY087990 | CY087992 | human | Scotland | 2003 |
| CY112396 | CY121295 | human | China:Sichuan | 1987 |
| KF533054 | KF533060 | human | Singapore | 2007 |
| KM069480 | KM069756 | human | Singapore | 2009 |
| CY112524 | CY112526 | human | South Austalia | 1992 |
| CY017395 | CY017397 | human | South Australia | 2000 |
| CY090901 | CY090903 | human | America:South Carolina | 1999 |
| CY112805 | CY112807 | human | Stockholm | 1995 |
| CY008212 | CY008214 | human | Tairawhiti | 2004 |
| CY104228 | CY104230 | human | TayNguyen | 2005 |
| CY105766 | CY105768 | human | TayNguyen | 2007 |
| CY105446 | CY105448 | human | TayNguyen | 2005 |
| CY026275 | CY026277 | human | America:Texas | 2007 |
| EU199365 | EU199417 | human | America:Vermont | 2007 |
| CY033457 | CY033459 | human | America:Vermont | 2007 |
| CY011768 | CY011770 | human | Waikato | 2003 |
| CY013429 | CY013431 | human | Waikato | 2003 |
| CY012704 | CY012706 | human | Waikato | 2004 |
| CY013919 | CY013921 | human | Waikato | 2004 |
| CY013525 | CY013527 | human | Waikato | 2004 |
| CY013541 | CY013543 | human | Waikato | 2004 |
| CY012056 | CY012058 | human | Waikato | 2002 |
| CY013943 | CY013945 | human | Waikato | 2004 |
| CY013967 | CY013969 | human | Waikato | 2004 |
| CY014063 | CY014065 | human | Waikato | 2005 |
| CY013144 | CY013146 | human | Waikato | 2003 |
| CY013469 | CY013471 | human | Wellington | 2004 |
| CY012696 | CY012698 | human | Wellington | 2004 |
| CY012064 | CY012066 | human | Wellington | 2003 |
| CY011632 | CY011634 | human | Wellington | 2002 |
| CY013160 | CY013162 | human | Wellington | 2004 |
| CY014095 | CY014097 | human | Wellington | 2005 |
| CY011672 | CY011674 | human | Wellington | 2002 |
| CY015820 | CY015822 | human | Western Australia | 2003 |
| CY015860 | CY015862 | human | Western Australia | 2003 |
| CY111522 | CY111524 | human | Western Australia | 2000 |
| CY016012 | CY016014 | human | Western Australia | 2005 |
| CY007331 | CY007333 | human | Whanganui | 2004 |
| CY163744 | CY114383 | human | America:Wisconsin | 2005 |
| EU268227 | EU268229 | human | America:Wyoming | 2003 |

Table S3. The RSCU of different H3N2 CIVs, including AIVs, CIVs and human influenza virus.

|  |  | Canis familiaris | | | | Homo sapiens | Gallus gallus |
| --- | --- | --- | --- | --- | --- | --- | --- |
| AA | Codon | H3N2 | China clade | Korea/USA clade | Origin clade | H3N2 | H3N2 |
| Phe | UUU(F) | 0.63 | 0.66 | 0.61 | 0.59 | 0.79 | 0.79 |
|  | UUC(F) | **1.37** | **1.34** | **1.39** | **1.41** | **1.21** | **1.21** |
| Leu | UUA(L) | 0.84 | 0.81 | 0.86 | 0.87 | 0.38 | 0.4 |
|  | UUG(L) | **1.55** | **1.61** | **1.48** | **1.62** | 1.44 | 1.14 |
|  | CUU(L) | 1.4 | 1.47 | 1.32 | 1.5 | 1.23 | 0.98 |
|  | CUC(L) | 0.08 | 0.01 | 0.15 | 0 | 0.33 | 0.25 |
|  | CUA(L) | 1.09 | 1.06 | 1.15 | 0.96 | 1.01 | 1.37 |
|  | CUG(L) | 1.04 | 1.04 | 1.04 | 1.05 | **1.6** | **1.85** |
| Ile | AUU(I) | **1.39** | **1.37** | **1.43** | **1.3** | 0.97 | **1.15** |
|  | AUC(I) | 0.64 | 0.66 | 0.6 | 0.75 | **1.14** | 0.83 |
|  | AUA(I) | 0.97 | 0.97 | 0.97 | 0.96 | 0.9 | 1.02 |
| Val | GUU(V) | 1 | 0.97 | 1.01 | 0.99 | **1.67** | 1.08 |
|  | GUC(V) | 0.85 | 0.85 | 0.87 | 0.82 | 0.48 | 0.69 |
|  | GUA(V) | **1.21** | **1.29** | **1.13** | **1.3** | 1.13 | **1.13** |
|  | GUG(V) | 0.94 | 0.89 | 0.99 | 0.89 | 0.72 | 1.1 |
| Ser | AGU(S) | 0.37 | 0.34 | 0.4 | 0.34 | 0.84 | 0.76 |
|  | AGC(S) | **1.9** | **1.88** | **1.91** | **1.92** | **1.95** | **1.72** |
|  | UCU(S) | 0.88 | 0.88 | 0.87 | 0.93 | 0.85 | 0.91 |
|  | UCC(S) | 1.15 | 1.18 | 1.14 | 1.13 | 0.64 | 0.74 |
|  | UCA(S) | 1.53 | 1.55 | 1.51 | 1.51 | 1.69 | 1.64 |
|  | UCG(S) | 0.17 | 0.17 | 0.17 | 0.17 | 0.05 | 0.23 |
| Pro | CCU(P) | 1.23 | 1.21 | 1.26 | **1.2** | 0.89 | **1.45** |
|  | CCC(P) | 1.02 | 1.01 | 1.05 | 1 | 1.07 | 0.92 |
|  | CCA(P) | **1.29** | **1.33** | **1.27** | **1.2** | **1.22** | 1.21 |
|  | CCG(P) | 0.46 | 0.46 | 0.41 | 0.6 | 0.82 | 0.42 |
| Thr | ACU(T) | 1.35 | 1.39 | 1.31 | 1.39 | **1.58** | 1.22 |
|  | ACC(T) | 0.9 | 0.85 | 0.96 | 0.86 | 0.33 | 0.9 |
|  | ACA(T) | **1.63** | **1.66** | **1.62** | **1.6** | 1.5 | **1.65** |
|  | ACG(T) | 0.11 | 0.11 | 0.11 | 0.15 | 0.59 | 0.23 |
| Ala | GCU(A) | 1.24 | 1.3 | 1.18 | 1.28 | 1.17 | 0.92 |
|  | GCC(A) | 0.95 | 0.87 | 1.02 | 0.94 | 1 | 1.05 |
|  | GCA(A) | **1.74** | **1.68** | **1.78** | **1.78** | **1.58** | **1.81** |
|  | GCG(A) | 0.08 | 0.15 | 0.03 | 0 | 0.24 | 0.22 |
| Tyr | UAU(Y) | **1.04** | 0.99 | **1.08** | **1** | 0.86 | **1.09** |
|  | UAC(Y) | 0.96 | **1.01** | 0.92 | **1** | **1.14** | 0.91 |
| His | CAU(H) | 0.91 | 0.91 | 0.9 | **1** | **1.06** | **1.32** |
|  | CAC(H) | **1.09** | **1.09** | **1.1** | **1** | 0.94 | 0.68 |
| Gln | CAA(Q) | **1.33** | **1.33** | **1.34** | **1.3** | **1.54** | **1.13** |
|  | CAG(Q) | 0.67 | 0.67 | 0.66 | 0.7 | 0.46 | 0.87 |
| Asn | AAU(N) | **1.16** | **1.18** | **1.13** | **1.2** | **1.11** | **1.13** |
|  | AAC(N) | 0.84 | 0.82 | 0.87 | 0.8 | 0.89 | 0.87 |
| Lys | AAA(K) | **1.25** | **1.23** | **1.29** | **1.19** | **1.63** | **1.3** |
|  | AAG(K) | 0.75 | 0.77 | 0.71 | 0.81 | 0.37 | 0.7 |
| Asp | GAU(D) | 0.68 | 0.67 | 0.67 | 0.72 | 0.91 | 0.98 |
|  | GAC(D) | **1.32** | **1.33** | **1.33** | **1.28** | **1.09** | **1.02** |
| Glu | GAA(E) | **1.3** | **1.3** | **1.3** | **1.32** | **1.13** | **1.18** |
|  | GAG(E) | 0.7 | 0.7 | 0.7 | 0.68 | 0.87 | 0.82 |
| Cys | UGU(C) | 0.78 | 0.82 | 0.77 | 0.69 | 0.84 | 0.76 |
|  | UGC(C) | **1.22** | **1.18** | **1.23** | **1.31** | **1.16** | **1.24** |
| Arg | AGA(R) | **2.52** | **2.47** | **2.57** | **2.49** | **2.82** | **2.72** |
|  | AGG(R) | 2.36 | 2.4 | 2.37 | 2.22 | 2.03 | 1.99 |
|  | CGU(R) | 0 | 0 | 0 | 0 | 0 | 0.04 |
|  | CGC(R) | 0.02 | 0 | 0 | 0.18 | 0.22 | 0.28 |
|  | CGA(R) | 0.66 | 0.68 | 0.64 | 0.67 | 0.44 | 0.53 |
|  | CGG(R) | 0.43 | 0.45 | 0.42 | 0.44 | 0.48 | 0.45 |
| Gly | GGU(G) | 0.69 | 0.7 | 0.65 | 0.8 | 0.83 | 0.82 |
|  | GGC(G) | 0.91 | 0.95 | 0.89 | 0.85 | 0.74 | 0.58 |
|  | GGA(G) | **1.6** | **1.55** | **1.65** | **1.55** | **1.41** | **1.58** |
|  | GGG(G) | 0.8 | 0.81 | 0.8 | 0.8 | 1.03 | 1.02 |
|  |  |  |  |  |  |  |  |

denote: the abundant codons are displayed in bold.

Table S4. CAI of H3N2 segments in relation to potential host species.

|  | HA | | | | MP | | | |
| --- | --- | --- | --- | --- | --- | --- | --- | --- |
| Strain name | *Homo sapiens* | *Canis familiaris* | *Felis catus* | *Gallus gallus* | *Homo sapiens* | *Canis familiaris* | *Felis catus* | *Gallus gallus* |
| A/canine/Zhejiang/1/2010 | 0.749 | 0.683 | 0.680 | 0.772 | 0.742 | 0.689 | 0.685 | 0.767 |
| A/canine/Wisconsin/19137/2016 | 0.749 | 0.682 | 0.680 | 0.770 | 0.741 | 0.687 | 0.684 | 0.765 |
| A/canine/Texas/343907/2015 | 0.750 | 0.684 | 0.681 | 0.772 | 0.741 | 0.687 | 0.684 | 0.765 |
| A/canine/South_Korea/0173915/2015 | 0.749 | 0.682 | 0.679 | 0.770 | 0.738 | 0.684 | 0.680 | 0.763 |
| A/canine/North_Carolina/109904/2015 | 0.749 | 0.682 | 0.679 | 0.770 | 0.740 | 0.687 | 0.683 | 0.764 |
| A/canine/Liaoning/H6/2012 | 0.744 | 0.677 | 0.674 | 0.766 | 0.743 | 0.689 | 0.685 | 0.768 |
| A/canine/Liaoning/27/2012 | 0.744 | 0.677 | 0.674 | 0.766 | 0.743 | 0.689 | 0.685 | 0.768 |
| A/canine/Korea/S1/2012 | 0.748 | 0.683 | 0.681 | 0.770 | 0.740 | 0.686 | 0.682 | 0.766 |
| A/canine/Korea/KRIBB01/2011 | 0.748 | 0.682 | 0.679 | 0.771 | 0.742 | 0.688 | 0.684 | 0.768 |
| A/canine/Korea/GCVP01/2007 | 0.750 | 0.684 | 0.680 | 0.773 | 0.737 | 0.684 | 0.681 | 0.761 |
| A/canine/Korea/DG1/2014 | 0.746 | 0.679 | 0.676 | 0.768 | 0.739 | 0.684 | 0.679 | 0.763 |
| A/canine/Korea/CY053/2014 | 0.750 | 0.684 | 0.681 | 0.772 | 0.744 | 0.690 | 0.685 | 0.770 |
| A/canine/Korea/CY009/2010 | 0.750 | 0.684 | 0.681 | 0.773 | 0.741 | 0.686 | 0.682 | 0.766 |
| A/canine/Korea/BD1/2013 | 0.748 | 0.681 | 0.678 | 0.769 | 0.745 | 0.690 | 0.686 | 0.770 |
| A/canine/Korea/0589318/2015 | 0.748 | 0.682 | 0.679 | 0.769 | 0.738 | 0.684 | 0.680 | 0.763 |
| A/canine/Korea/01/2007 | 0.749 | 0.683 | 0.679 | 0.772 | 0.745 | 0.692 | 0.688 | 0.769 |
| A/canine/Jiangsu/06/2010 | 0.748 | 0.682 | 0.678 | 0.770 | 0.742 | 0.688 | 0.684 | 0.767 |
| A/canine/Jiangsu/05/2010 | 0.746 | 0.680 | 0.676 | 0.769 | 0.743 | 0.690 | 0.686 | 0.767 |
| A/canine/Jiangsu/04/2010 | 0.746 | 0.680 | 0.676 | 0.769 | 0.743 | 0.690 | 0.686 | 0.767 |
| A/canine/Jiangsu/03/2010 | 0.747 | 0.681 | 0.677 | 0.770 | 0.743 | 0.690 | 0.686 | 0.767 |
| A/canine/Jiangsu/02/2010 | 0.746 | 0.680 | 0.677 | 0.770 | 0.743 | 0.690 | 0.686 | 0.767 |
| A/canine/Jiangsu/01/2009 | 0.744 | 0.678 | 0.674 | 0.767 | 0.743 | 0.690 | 0.686 | 0.767 |
| A/canine/Indiana/96198/2015 | 0.750 | 0.683 | 0.681 | 0.771 | 0.741 | 0.687 | 0.684 | 0.765 |
| A/canine/Indiana/003018/2016 | 0.749 | 0.683 | 0.680 | 0.770 | 0.741 | 0.687 | 0.684 | 0.765 |
| A/canine/Illinois/1619144/2015 | 0.750 | 0.683 | 0.680 | 0.771 | 0.741 | 0.687 | 0.684 | 0.765 |
| A/canine/Illinois/328292/2015 | 0.750 | 0.684 | 0.681 | 0.771 | 0.741 | 0.687 | 0.684 | 0.765 |
| A/canine/Illinois/283066/2015 | 0.749 | 0.682 | 0.680 | 0.770 | 0.741 | 0.687 | 0.684 | 0.765 |
| A/canine/Illinois/077753/2016 | 0.751 | 0.685 | 0.682 | 0.773 | 0.739 | 0.686 | 0.683 | 0.763 |
| A/canine/Illinois/12191/2015 | 0.748 | 0.681 | 0.678 | 0.768 | 0.741 | 0.687 | 0.684 | 0.765 |
| A/canine/Heilongjiang/L1/2013 | 0.746 | 0.679 | 0.676 | 0.768 | 0.739 | 0.685 | 0.682 | 0.764 |
| A/canine/Guangdong/23/2012 | 0.745 | 0.679 | 0.676 | 0.767 | 0.741 | 0.686 | 0.682 | 0.764 |
| A/canine/Guangdong/12/2012 | 0.746 | 0.680 | 0.677 | 0.769 | 0.741 | 0.686 | 0.682 | 0.764 |
| A/canine/Guangdong/05/2011 | 0.744 | 0.678 | 0.674 | 0.766 | 0.742 | 0.688 | 0.684 | 0.767 |
| A/canine/Guangdong/3/2011 | 0.747 | 0.680 | 0.677 | 0.771 | 0.742 | 0.689 | 0.685 | 0.767 |
| A/canine/Guangdong/2/2011 | 0.747 | 0.680 | 0.677 | 0.769 | 0.741 | 0.688 | 0.684 | 0.766 |
| A/canine/Guangdong/2/2006 | 0.746 | 0.679 | 0.676 | 0.769 | 0.740 | 0.687 | 0.683 | 0.765 |
| A/canine/Guangdong/1/2007 | 0.746 | 0.680 | 0.677 | 0.770 | 0.740 | 0.687 | 0.683 | 0.764 |
| A/canine/Guangdong/1/2006 | 0.744 | 0.679 | 0.675 | 0.768 | 0.742 | 0.689 | 0.686 | 0.766 |
| A/canine/Georgia/104940/2015 | 0.749 | 0.682 | 0.679 | 0.770 | 0.740 | 0.687 | 0.683 | 0.765 |
| A/canine/Georgia/95391/2015 | 0.750 | 0.683 | 0.681 | 0.771 | 0.740 | 0.687 | 0.683 | 0.765 |
| A/canine/Florida/269770/2015 | 0.748 | 0.682 | 0.679 | 0.770 | 0.741 | 0.687 | 0.684 | 0.765 |
| A/canine/China/JLM2/2015 | 0.743 | 0.675 | 0.671 | 0.765 | 0.737 | 0.682 | 0.678 | 0.763 |
| A/canine/China/JLM1/2015 | 0.741 | 0.674 | 0.671 | 0.765 | 0.743 | 0.687 | 0.683 | 0.768 |
| Avrerage | 0.747 | 0.681 | 0.678 | 0.770 | 0.741 | 0.687 | 0.684 | 0.766 |
| SD | 0.002 | 0.002 | 0.003 | 0.002 | 0.002 | 0.002 | 0.002 | 0.002 |
|  | NA | | | | NP | | | |
| Strain name | *Homo sapiens* | *Canis familiaris* | *Felis catus* | *Gallus gallus* | *Homo sapiens* | *Canis familiaris* | *Felis catus* | *Gallus gallus* |
| A/canine/Zhejiang/1/2010 | 0.721 | 0.650 | 0.643 | 0.739 | 0.750 | 0.688 | 0.684 | 0.784 |
| A/canine/Wisconsin/19137/2016 | 0.723 | 0.651 | 0.644 | 0.741 | 0.741 | 0.679 | 0.675 | 0.774 |
| A/canine/Texas/343907/2015 | 0.722 | 0.650 | 0.644 | 0.741 | 0.740 | 0.679 | 0.674 | 0.773 |
| A/canine/South_Korea/0173915/2015 | 0.722 | 0.651 | 0.643 | 0.742 | 0.743 | 0.681 | 0.676 | 0.776 |
| A/canine/North_Carolina/109904/2015 | 0.721 | 0.649 | 0.642 | 0.741 | 0.741 | 0.678 | 0.674 | 0.774 |
| A/canine/Liaoning/H6/2012 | 0.721 | 0.650 | 0.644 | 0.740 | 0.750 | 0.689 | 0.685 | 0.784 |
| A/canine/Liaoning/27/2012 | 0.721 | 0.650 | 0.644 | 0.740 | 0.749 | 0.687 | 0.683 | 0.782 |
| A/canine/Korea/S1/2012 | 0.723 | 0.652 | 0.645 | 0.743 | 0.748 | 0.687 | 0.682 | 0.781 |
| A/canine/Korea/KRIBB01/2011 | 0.724 | 0.652 | 0.645 | 0.744 | 0.746 | 0.684 | 0.681 | 0.780 |
| A/canine/Korea/GCVP01/2007 | 0.730 | 0.660 | 0.652 | 0.750 | 0.752 | 0.691 | 0.686 | 0.786 |
| A/canine/Korea/DG1/2014 | 0.726 | 0.655 | 0.649 | 0.745 | 0.745 | 0.684 | 0.680 | 0.779 |
| A/canine/Korea/CY053/2014 | 0.727 | 0.655 | 0.648 | 0.746 | 0.745 | 0.684 | 0.680 | 0.779 |
| A/canine/Korea/CY009/2010 | 0.728 | 0.656 | 0.649 | 0.746 | 0.743 | 0.682 | 0.678 | 0.777 |
| A/canine/Korea/BD1/2013 | 0.724 | 0.652 | 0.646 | 0.744 | 0.744 | 0.683 | 0.679 | 0.778 |
| A/canine/Korea/0589318/2015 | 0.722 | 0.651 | 0.643 | 0.742 | 0.743 | 0.681 | 0.676 | 0.776 |
| A/canine/Korea/01/2007 | 0.730 | 0.660 | 0.652 | 0.750 | 0.751 | 0.690 | 0.685 | 0.786 |
| A/canine/Jiangsu/06/2010 | 0.724 | 0.652 | 0.646 | 0.744 | 0.750 | 0.689 | 0.684 | 0.784 |
| A/canine/Jiangsu/05/2010 | 0.724 | 0.653 | 0.647 | 0.743 | 0.749 | 0.688 | 0.684 | 0.783 |
| A/canine/Jiangsu/04/2010 | 0.726 | 0.655 | 0.649 | 0.746 | 0.749 | 0.688 | 0.684 | 0.783 |
| A/canine/Jiangsu/03/2010 | 0.724 | 0.653 | 0.647 | 0.743 | 0.749 | 0.688 | 0.684 | 0.783 |
| A/canine/Jiangsu/02/2010 | 0.724 | 0.653 | 0.647 | 0.743 | 0.749 | 0.688 | 0.684 | 0.783 |
| A/canine/Jiangsu/01/2009 | 0.724 | 0.653 | 0.647 | 0.743 | 0.749 | 0.688 | 0.684 | 0.783 |
| A/canine/Indiana/96198/2015 | 0.723 | 0.651 | 0.644 | 0.742 | 0.741 | 0.680 | 0.675 | 0.774 |
| A/canine/Indiana/003018/2016 | 0.722 | 0.650 | 0.644 | 0.741 | 0.739 | 0.677 | 0.673 | 0.772 |
| A/canine/Illinois/1619144/2015 | 0.723 | 0.651 | 0.644 | 0.741 | 0.740 | 0.678 | 0.674 | 0.773 |
| A/canine/Illinois/328292/2015 | 0.723 | 0.651 | 0.644 | 0.742 | 0.740 | 0.679 | 0.674 | 0.773 |
| A/canine/Illinois/283066/2015 | 0.723 | 0.651 | 0.644 | 0.742 | 0.741 | 0.680 | 0.675 | 0.774 |
| A/canine/Illinois/077753/2016 | 0.722 | 0.650 | 0.643 | 0.741 | 0.740 | 0.678 | 0.674 | 0.773 |
| A/canine/Illinois/12191/2015 | 0.722 | 0.651 | 0.644 | 0.742 | 0.741 | 0.679 | 0.675 | 0.774 |
| A/canine/Heilongjiang/L1/2013 | 0.724 | 0.653 | 0.648 | 0.743 | 0.749 | 0.687 | 0.683 | 0.782 |
| A/canine/Guangdong/23/2012 | 0.727 | 0.654 | 0.649 | 0.746 | 0.751 | 0.689 | 0.685 | 0.784 |
| A/canine/Guangdong/12/2012 | 0.728 | 0.656 | 0.650 | 0.748 | 0.748 | 0.686 | 0.681 | 0.781 |
| A/canine/Guangdong/05/2011 | 0.723 | 0.651 | 0.645 | 0.743 | 0.750 | 0.689 | 0.684 | 0.784 |
| A/canine/Guangdong/3/2011 | 0.725 | 0.655 | 0.649 | 0.745 | 0.748 | 0.687 | 0.683 | 0.782 |
| A/canine/Guangdong/2/2011 | 0.727 | 0.656 | 0.650 | 0.747 | 0.748 | 0.687 | 0.683 | 0.782 |
| A/canine/Guangdong/2/2006 | 0.727 | 0.656 | 0.649 | 0.747 | 0.750 | 0.690 | 0.686 | 0.785 |
| A/canine/Guangdong/1/2007 | 0.727 | 0.656 | 0.649 | 0.747 | 0.754 | 0.693 | 0.689 | 0.788 |
| A/canine/Guangdong/1/2006 | 0.729 | 0.658 | 0.651 | 0.749 | 0.753 | 0.693 | 0.689 | 0.787 |
| A/canine/Georgia/104940/2015 | 0.723 | 0.651 | 0.644 | 0.741 | 0.741 | 0.678 | 0.674 | 0.774 |
| A/canine/Georgia/95391/2015 | 0.723 | 0.651 | 0.644 | 0.741 | 0.741 | 0.678 | 0.674 | 0.774 |
| A/canine/Florida/269770/2015 | 0.721 | 0.649 | 0.642 | 0.740 | 0.740 | 0.677 | 0.673 | 0.773 |
| A/canine/China/JLM2/2015 | 0.721 | 0.648 | 0.643 | 0.741 | 0.747 | 0.685 | 0.681 | 0.781 |
| A/canine/China/JLM1/2015 | 0.722 | 0.649 | 0.644 | 0.741 | 0.749 | 0.686 | 0.683 | 0.783 |
| Avrerage | 0.724 | 0.653 | 0.646 | 0.743 | 0.746 | 0.684 | 0.680 | 0.780 |
| SD | 0.003 | 0.003 | 0.003 | 0.003 | 0.004 | 0.005 | 0.005 | 0.005 |
|  | NS | | | | PA | | | |
| Strain name | *Homo sapiens* | *Canis familiaris* | *Felis catus* | *Gallus gallus* | *Homo sapiens* | *Canis familiaris* | *Felis catus* | *Gallus gallus* |
| A/canine/Zhejiang/1/2010 | 0.717 | 0.653 | 0.653 | 0.746 | 0.748 | 0.682 | 0.684 | 0.773 |
| A/canine/Wisconsin/19137/2016 | 0.715 | 0.648 | 0.648 | 0.743 | 0.747 | 0.680 | 0.683 | 0.770 |
| A/canine/Texas/343907/2015 | 0.715 | 0.648 | 0.649 | 0.743 | 0.748 | 0.682 | 0.684 | 0.772 |
| A/canine/South_Korea/0173915/2015 | 0.719 | 0.652 | 0.653 | 0.749 | 0.748 | 0.681 | 0.684 | 0.772 |
| A/canine/North_Carolina/109904/2015 | 0.715 | 0.648 | 0.649 | 0.743 | 0.750 | 0.684 | 0.686 | 0.774 |
| A/canine/Liaoning/H6/2012 | 0.720 | 0.655 | 0.655 | 0.749 | 0.746 | 0.681 | 0.683 | 0.771 |
| A/canine/Liaoning/27/2012 | 0.720 | 0.655 | 0.655 | 0.749 | 0.746 | 0.680 | 0.683 | 0.771 |
| A/canine/Korea/S1/2012 | 0.719 | 0.654 | 0.655 | 0.748 | 0.748 | 0.682 | 0.684 | 0.772 |
| A/canine/Korea/KRIBB01/2011 | 0.721 | 0.655 | 0.655 | 0.750 | 0.752 | 0.686 | 0.688 | 0.776 |
| A/canine/Korea/GCVP01/2007 | 0.722 | 0.658 | 0.658 | 0.752 | 0.755 | 0.689 | 0.691 | 0.780 |
| A/canine/Korea/DG1/2014 | 0.717 | 0.651 | 0.651 | 0.744 | 0.750 | 0.684 | 0.686 | 0.775 |
| A/canine/Korea/CY053/2014 | 0.718 | 0.653 | 0.653 | 0.747 | 0.752 | 0.686 | 0.689 | 0.777 |
| A/canine/Korea/CY009/2010 | 0.715 | 0.651 | 0.652 | 0.746 | 0.751 | 0.686 | 0.688 | 0.776 |
| A/canine/Korea/BD1/2013 | 0.721 | 0.654 | 0.654 | 0.751 | 0.750 | 0.684 | 0.687 | 0.775 |
| A/canine/Korea/0589318/2015 | 0.719 | 0.652 | 0.653 | 0.749 | 0.748 | 0.681 | 0.684 | 0.772 |
| A/canine/Korea/01/2007 | 0.718 | 0.655 | 0.656 | 0.748 | 0.756 | 0.690 | 0.693 | 0.780 |
| A/canine/Jiangsu/06/2010 | 0.710 | 0.646 | 0.646 | 0.739 | 0.745 | 0.679 | 0.681 | 0.770 |
| A/canine/Jiangsu/05/2010 | 0.715 | 0.651 | 0.651 | 0.745 | 0.745 | 0.679 | 0.681 | 0.770 |
| A/canine/Jiangsu/04/2010 | 0.715 | 0.651 | 0.651 | 0.745 | 0.746 | 0.680 | 0.683 | 0.771 |
| A/canine/Jiangsu/03/2010 | 0.715 | 0.651 | 0.651 | 0.745 | 0.746 | 0.680 | 0.683 | 0.771 |
| A/canine/Jiangsu/02/2010 | 0.713 | 0.650 | 0.650 | 0.743 | 0.746 | 0.680 | 0.683 | 0.771 |
| A/canine/Jiangsu/01/2009 | 0.715 | 0.651 | 0.651 | 0.745 | 0.746 | 0.680 | 0.683 | 0.771 |
| A/canine/Indiana/96198/2015 | 0.715 | 0.648 | 0.649 | 0.743 | 0.747 | 0.681 | 0.683 | 0.771 |
| A/canine/Indiana/003018/2016 | 0.715 | 0.648 | 0.649 | 0.743 | 0.748 | 0.682 | 0.684 | 0.772 |
| A/canine/Illinois/1619144/2015 | 0.715 | 0.648 | 0.649 | 0.743 | 0.749 | 0.682 | 0.685 | 0.773 |
| A/canine/Illinois/328292/2015 | 0.715 | 0.648 | 0.649 | 0.743 | 0.748 | 0.682 | 0.684 | 0.772 |
| A/canine/Illinois/283066/2015 | 0.715 | 0.648 | 0.649 | 0.743 | 0.748 | 0.681 | 0.683 | 0.771 |
| A/canine/Illinois/077753/2016 | 0.715 | 0.648 | 0.649 | 0.743 | 0.746 | 0.680 | 0.682 | 0.769 |
| A/canine/Illinois/12191/2015 | 0.715 | 0.648 | 0.649 | 0.743 | 0.749 | 0.683 | 0.685 | 0.774 |
| A/canine/Heilongjiang/L1/2013 | 0.721 | 0.656 | 0.656 | 0.749 | 0.748 | 0.682 | 0.685 | 0.773 |
| A/canine/Guangdong/23/2012 | 0.711 | 0.646 | 0.647 | 0.741 | 0.743 | 0.677 | 0.680 | 0.767 |
| A/canine/Guangdong/12/2012 | 0.711 | 0.646 | 0.647 | 0.741 | 0.743 | 0.677 | 0.680 | 0.767 |
| A/canine/Guangdong/05/2011 | 0.712 | 0.648 | 0.649 | 0.742 | 0.743 | 0.677 | 0.680 | 0.769 |
| A/canine/Guangdong/3/2011 | 0.712 | 0.648 | 0.648 | 0.744 | 0.751 | 0.685 | 0.688 | 0.777 |
| A/canine/Guangdong/2/2011 | 0.712 | 0.648 | 0.648 | 0.744 | 0.747 | 0.681 | 0.683 | 0.772 |
| A/canine/Guangdong/2/2006 | 0.712 | 0.648 | 0.648 | 0.741 | 0.752 | 0.687 | 0.689 | 0.777 |
| A/canine/Guangdong/1/2007 | 0.719 | 0.654 | 0.653 | 0.749 | 0.752 | 0.687 | 0.689 | 0.777 |
| A/canine/Guangdong/1/2006 | 0.714 | 0.650 | 0.650 | 0.745 | 0.757 | 0.692 | 0.694 | 0.782 |
| A/canine/Georgia/104940/2015 | 0.715 | 0.648 | 0.649 | 0.743 | 0.749 | 0.683 | 0.686 | 0.773 |
| A/canine/Georgia/95391/2015 | 0.715 | 0.648 | 0.649 | 0.743 | 0.750 | 0.684 | 0.686 | 0.774 |
| A/canine/Florida/269770/2015 | 0.715 | 0.648 | 0.649 | 0.743 | 0.750 | 0.684 | 0.686 | 0.774 |
| A/canine/China/JLM2/2015 | 0.707 | 0.642 | 0.643 | 0.737 | 0.744 | 0.677 | 0.679 | 0.768 |
| A/canine/China/JLM1/2015 | 0.708 | 0.643 | 0.644 | 0.737 | 0.745 | 0.678 | 0.680 | 0.769 |
| Avrerage | 0.715 | 0.650 | 0.651 | 0.745 | 0.748 | 0.682 | 0.685 | 0.773 |
| SD | 0.003 | 0.003 | 0.003 | 0.003 | 0.003 | 0.003 | 0.003 | 0.003 |
|  | PB1 | | | | PB2 | | | |
| Strain name | *Homo sapiens* | *Canis familiaris* | *Felis catus* | *Gallus gallus* | *Homo sapiens* | *Canis familiaris* | *Felis catus* | *Gallus gallus* |
| A/canine/Zhejiang/1/2010 | 0.760 | 0.696 | 0.693 | 0.790 | 0.726 | 0.663 | 0.669 | 0.760 |
| A/canine/Wisconsin/19137/2016 | 0.754 | 0.688 | 0.684 | 0.783 | 0.719 | 0.655 | 0.654 | 0.754 |
| A/canine/Texas/343907/2015 | 0.754 | 0.688 | 0.684 | 0.784 | 0.719 | 0.655 | 0.654 | 0.754 |
| A/canine/South_Korea/0173915/2015 | 0.756 | 0.690 | 0.686 | 0.786 | 0.719 | 0.656 | 0.655 | 0.754 |
| A/canine/North_Carolina/109904/2015 | 0.755 | 0.689 | 0.685 | 0.785 | 0.719 | 0.655 | 0.654 | 0.753 |
| A/canine/Liaoning/H6/2012 | 0.758 | 0.693 | 0.689 | 0.788 | 0.718 | 0.654 | 0.653 | 0.753 |
| A/canine/Liaoning/27/2012 | 0.758 | 0.693 | 0.689 | 0.788 | 0.719 | 0.655 | 0.654 | 0.753 |
| A/canine/Korea/S1/2012 | 0.761 | 0.696 | 0.692 | 0.791 | 0.728 | 0.665 | 0.664 | 0.763 |
| A/canine/Korea/KRIBB01/2011 | 0.761 | 0.697 | 0.693 | 0.791 | 0.718 | 0.655 | 0.654 | 0.752 |
| A/canine/Korea/GCVP01/2007 | 0.764 | 0.701 | 0.697 | 0.794 | 0.726 | 0.664 | 0.662 | 0.761 |
| A/canine/Korea/DG1/2014 | 0.764 | 0.700 | 0.695 | 0.794 | 0.722 | 0.659 | 0.658 | 0.757 |
| A/canine/Korea/CY053/2014 | 0.762 | 0.697 | 0.693 | 0.793 | 0.723 | 0.660 | 0.659 | 0.757 |
| A/canine/Korea/CY009/2010 | 0.761 | 0.696 | 0.692 | 0.791 | 0.725 | 0.662 | 0.661 | 0.759 |
| A/canine/Korea/BD1/2013 | 0.757 | 0.692 | 0.688 | 0.787 | 0.725 | 0.661 | 0.660 | 0.759 |
| A/canine/Korea/0589318/2015 | 0.756 | 0.690 | 0.686 | 0.786 | 0.720 | 0.657 | 0.656 | 0.755 |
| A/canine/Korea/01/2007 | 0.765 | 0.702 | 0.698 | 0.796 | 0.726 | 0.664 | 0.662 | 0.761 |
| A/canine/Jiangsu/06/2010 | 0.757 | 0.692 | 0.689 | 0.787 | 0.725 | 0.661 | 0.659 | 0.758 |
| A/canine/Jiangsu/05/2010 | 0.759 | 0.694 | 0.691 | 0.789 | 0.724 | 0.661 | 0.660 | 0.758 |
| A/canine/Jiangsu/04/2010 | 0.759 | 0.695 | 0.692 | 0.789 | 0.723 | 0.660 | 0.658 | 0.757 |
| A/canine/Jiangsu/03/2010 | 0.759 | 0.695 | 0.692 | 0.789 | 0.724 | 0.662 | 0.660 | 0.758 |
| A/canine/Jiangsu/02/2010 | 0.759 | 0.695 | 0.691 | 0.789 | 0.724 | 0.661 | 0.660 | 0.758 |
| A/canine/Jiangsu/01/2009 | 0.759 | 0.694 | 0.691 | 0.788 | 0.724 | 0.661 | 0.660 | 0.758 |
| A/canine/Indiana/96198/2015 | 0.754 | 0.689 | 0.685 | 0.784 | 0.720 | 0.656 | 0.655 | 0.755 |
| A/canine/Indiana/003018/2016 | 0.754 | 0.688 | 0.684 | 0.784 | 0.719 | 0.655 | 0.654 | 0.754 |
| A/canine/Illinois/1619144/2015 | 0.755 | 0.689 | 0.685 | 0.784 | 0.720 | 0.656 | 0.655 | 0.754 |
| A/canine/Illinois/328292/2015 | 0.754 | 0.688 | 0.684 | 0.784 | 0.719 | 0.655 | 0.654 | 0.754 |
| A/canine/Illinois/283066/2015 | 0.755 | 0.689 | 0.685 | 0.784 | 0.718 | 0.654 | 0.653 | 0.753 |
| A/canine/Illinois/077753/2016 | 0.754 | 0.688 | 0.684 | 0.784 | 0.720 | 0.656 | 0.655 | 0.755 |
| A/canine/Illinois/12191/2015 | 0.755 | 0.689 | 0.685 | 0.785 | 0.720 | 0.656 | 0.656 | 0.755 |
| A/canine/Heilongjiang/L1/2013 | 0.760 | 0.695 | 0.691 | 0.790 | 0.723 | 0.659 | 0.658 | 0.757 |
| A/canine/Guangdong/23/2012 | 0.756 | 0.691 | 0.687 | 0.786 | 0.723 | 0.658 | 0.656 | 0.756 |
| A/canine/Guangdong/12/2012 | 0.756 | 0.691 | 0.688 | 0.786 | 0.722 | 0.657 | 0.656 | 0.756 |
| A/canine/Guangdong/05/2011 | 0.761 | 0.697 | 0.694 | 0.791 | 0.723 | 0.660 | 0.659 | 0.757 |
| A/canine/Guangdong/3/2011 | 0.762 | 0.698 | 0.694 | 0.792 | 0.723 | 0.660 | 0.658 | 0.758 |
| A/canine/Guangdong/2/2011 | 0.763 | 0.700 | 0.696 | 0.793 | 0.722 | 0.659 | 0.658 | 0.757 |
| A/canine/Guangdong/2/2006 | 0.764 | 0.700 | 0.697 | 0.794 | 0.727 | 0.665 | 0.663 | 0.762 |
| A/canine/Guangdong/1/2007 | 0.763 | 0.700 | 0.696 | 0.793 | 0.724 | 0.661 | 0.660 | 0.758 |
| A/canine/Guangdong/1/2006 | 0.765 | 0.699 | 0.695 | 0.794 | 0.727 | 0.664 | 0.661 | 0.762 |
| A/canine/Georgia/104940/2015 | 0.755 | 0.689 | 0.685 | 0.785 | 0.719 | 0.655 | 0.654 | 0.754 |
| A/canine/Georgia/95391/2015 | 0.755 | 0.689 | 0.685 | 0.785 | 0.719 | 0.655 | 0.654 | 0.753 |
| A/canine/Florida/269770/2015 | 0.755 | 0.689 | 0.685 | 0.785 | 0.720 | 0.655 | 0.654 | 0.754 |
| A/canine/China/JLM2/2015 | 0.757 | 0.692 | 0.689 | 0.787 | 0.723 | 0.658 | 0.656 | 0.757 |
| A/canine/China/JLM1/2015 | 0.757 | 0.692 | 0.689 | 0.787 | 0.722 | 0.657 | 0.655 | 0.756 |
| Avrerage | 0.758 | 0.693 | 0.690 | 0.788 | 0.722 | 0.659 | 0.657 | 0.756 |
| SD | 0.003 | 0.004 | 0.004 | 0.004 | 0.003 | 0.003 | 0.004 | 0.003 |

Table S5. Codon usage bias of H3N2 segments in relation to potential host species as estimated by RCDI.

|  | HA | | | | MP | | | |
| --- | --- | --- | --- | --- | --- | --- | --- | --- |
| Strain name | *Homo sapiens* | *Canis familiaris* | *Felis catus* | *Gallus gallus* | *Homo sapiens* | *Canis familiaris* | *Felis catus* | *Gallus gallus* |
| A/canine/Zhejiang/1/2010 | 1.261 | 1.332 | 1.323 | 1.245 | 1.196 | 1.235 | 1.241 | 1.200 |
| A/canine/Wisconsin/19137/2016 | 1.275 | 1.350 | 1.340 | 1.261 | 1.205 | 1.241 | 1.249 | 1.211 |
| A/canine/Texas/343907/2015 | 1.271 | 1.345 | 1.335 | 1.257 | 1.203 | 1.239 | 1.247 | 1.209 |
| A/canine/South_Korea/0173915/2015 | 1.269 | 1.343 | 1.334 | 1.255 | 1.205 | 1.246 | 1.255 | 1.209 |
| A/canine/North_Carolina/109904/2015 | 1.269 | 1.343 | 1.334 | 1.255 | 1.204 | 1.241 | 1.248 | 1.210 |
| A/canine/Liaoning/H6/2012 | 1.284 | 1.361 | 1.353 | 1.269 | 1.183 | 1.225 | 1.230 | 1.184 |
| A/canine/Liaoning/27/2012 | 1.284 | 1.361 | 1.353 | 1.269 | 1.183 | 1.225 | 1.230 | 1.184 |
| A/canine/Korea/S1/2012 | 1.266 | 1.336 | 1.322 | 1.251 | 1.193 | 1.234 | 1.240 | 1.193 |
| A/canine/Korea/KRIBB01/2011 | 1.261 | 1.335 | 1.324 | 1.246 | 1.185 | 1.226 | 1.232 | 1.186 |
| A/canine/Korea/GCVP01/2007 | 1.258 | 1.330 | 1.324 | 1.241 | 1.207 | 1.246 | 1.253 | 1.213 |
| A/canine/Korea/DG1/2014 | 1.272 | 1.350 | 1.340 | 1.257 | 1.194 | 1.238 | 1.246 | 1.197 |
| A/canine/Korea/CY053/2014 | 1.259 | 1.332 | 1.322 | 1.245 | 1.182 | 1.222 | 1.227 | 1.181 |
| A/canine/Korea/CY009/2010 | 1.255 | 1.327 | 1.318 | 1.240 | 1.188 | 1.229 | 1.236 | 1.189 |
| A/canine/Korea/BD1/2013 | 1.281 | 1.356 | 1.345 | 1.267 | 1.191 | 1.232 | 1.239 | 1.194 |
| A/canine/Korea/0589318/2015 | 1.272 | 1.348 | 1.338 | 1.259 | 1.205 | 1.246 | 1.255 | 1.209 |
| A/canine/Korea/01/2007 | 1.263 | 1.336 | 1.331 | 1.246 | 1.205 | 1.244 | 1.249 | 1.209 |
| A/canine/Jiangsu/06/2010 | 1.257 | 1.330 | 1.321 | 1.242 | 1.206 | 1.248 | 1.254 | 1.209 |
| A/canine/Jiangsu/05/2010 | 1.262 | 1.337 | 1.331 | 1.247 | 1.203 | 1.243 | 1.249 | 1.208 |
| A/canine/Jiangsu/04/2010 | 1.257 | 1.331 | 1.325 | 1.242 | 1.203 | 1.243 | 1.249 | 1.208 |
| A/canine/Jiangsu/03/2010 | 1.262 | 1.336 | 1.329 | 1.248 | 1.203 | 1.243 | 1.249 | 1.208 |
| A/canine/Jiangsu/02/2010 | 1.260 | 1.335 | 1.327 | 1.244 | 1.203 | 1.243 | 1.249 | 1.208 |
| A/canine/Jiangsu/01/2009 | 1.268 | 1.344 | 1.337 | 1.253 | 1.203 | 1.243 | 1.249 | 1.208 |
| A/canine/Indiana/96198/2015 | 1.274 | 1.349 | 1.338 | 1.260 | 1.203 | 1.239 | 1.247 | 1.209 |
| A/canine/Indiana/003018/2016 | 1.273 | 1.348 | 1.339 | 1.260 | 1.203 | 1.239 | 1.247 | 1.209 |
| A/canine/Illinois/1619144/2015 | 1.266 | 1.341 | 1.331 | 1.253 | 1.203 | 1.239 | 1.247 | 1.209 |
| A/canine/Illinois/328292/2015 | 1.269 | 1.343 | 1.334 | 1.256 | 1.203 | 1.239 | 1.247 | 1.209 |
| A/canine/Illinois/283066/2015 | 1.275 | 1.350 | 1.340 | 1.262 | 1.203 | 1.239 | 1.247 | 1.209 |
| A/canine/Illinois/077753/2016 | 1.271 | 1.346 | 1.336 | 1.257 | 1.204 | 1.239 | 1.246 | 1.210 |
| A/canine/Illinois/12191/2015 | 1.271 | 1.346 | 1.337 | 1.260 | 1.203 | 1.239 | 1.247 | 1.209 |
| A/canine/Heilongjiang/L1/2013 | 1.274 | 1.350 | 1.344 | 1.260 | 1.196 | 1.237 | 1.241 | 1.198 |
| A/canine/Guangdong/23/2012 | 1.275 | 1.349 | 1.342 | 1.259 | 1.195 | 1.237 | 1.244 | 1.199 |
| A/canine/Guangdong/12/2012 | 1.273 | 1.347 | 1.340 | 1.258 | 1.195 | 1.237 | 1.244 | 1.199 |
| A/canine/Guangdong/05/2011 | 1.278 | 1.355 | 1.349 | 1.264 | 1.205 | 1.246 | 1.253 | 1.208 |
| A/canine/Guangdong/3/2011 | 1.268 | 1.343 | 1.336 | 1.251 | 1.199 | 1.239 | 1.243 | 1.202 |
| A/canine/Guangdong/2/2011 | 1.273 | 1.348 | 1.340 | 1.259 | 1.199 | 1.239 | 1.244 | 1.203 |
| A/canine/Guangdong/2/2006 | 1.278 | 1.358 | 1.352 | 1.258 | 1.215 | 1.254 | 1.261 | 1.220 |
| A/canine/Guangdong/1/2007 | 1.271 | 1.346 | 1.340 | 1.251 | 1.210 | 1.249 | 1.256 | 1.215 |
| A/canine/Guangdong/1/2006 | 1.271 | 1.344 | 1.339 | 1.253 | 1.226 | 1.265 | 1.270 | 1.233 |
| A/canine/Georgia/104940/2015 | 1.269 | 1.343 | 1.334 | 1.255 | 1.208 | 1.246 | 1.253 | 1.214 |
| A/canine/Georgia/95391/2015 | 1.267 | 1.342 | 1.332 | 1.254 | 1.208 | 1.246 | 1.253 | 1.214 |
| A/canine/Florida/269770/2015 | 1.266 | 1.341 | 1.332 | 1.253 | 1.203 | 1.239 | 1.247 | 1.209 |
| A/canine/China/JLM2/2015 | 1.287 | 1.368 | 1.363 | 1.272 | 1.198 | 1.245 | 1.251 | 1.197 |
| A/canine/China/JLM1/2015 | 1.289 | 1.371 | 1.365 | 1.273 | 1.203 | 1.250 | 1.255 | 1.201 |
| Average | 1.270 | 1.345 | 1.336 | 1.255 | 1.201 | 1.240 | 1.247 | 1.205 |
| SD | 0.008 | 0.010 | 0.011 | 0.008 | 0.008 | 0.008 | 0.008 | 0.010 |
|  | NA | | | | NP | | | |
| Strain name | *Homo sapiens* | *Canis familiaris* | *Felis catus* | *Gallus gallus* | *Homo sapiens* | *Canis familiaris* | *Felis catus* | *Gallus gallus* |
| A/canine/Zhejiang/1/2010 | 1.344 | 1.445 | 1.445 | 1.334 | 1.287 | 1.354 | 1.345 | 1.265 |
| A/canine/Wisconsin/19137/2016 | 1.325 | 1.428 | 1.428 | 1.317 | 1.329 | 1.400 | 1.391 | 1.309 |
| A/canine/Texas/343907/2015 | 1.320 | 1.421 | 1.423 | 1.312 | 1.328 | 1.398 | 1.390 | 1.307 |
| A/canine/South_Korea/0173915/2015 | 1.320 | 1.421 | 1.424 | 1.310 | 1.330 | 1.400 | 1.392 | 1.309 |
| A/canine/North_Carolina/109904/2015 | 1.331 | 1.434 | 1.438 | 1.322 | 1.327 | 1.399 | 1.390 | 1.307 |
| A/canine/Liaoning/H6/2012 | 1.342 | 1.444 | 1.440 | 1.334 | 1.271 | 1.333 | 1.322 | 1.247 |
| A/canine/Liaoning/27/2012 | 1.342 | 1.444 | 1.440 | 1.334 | 1.270 | 1.334 | 1.324 | 1.247 |
| A/canine/Korea/S1/2012 | 1.326 | 1.426 | 1.427 | 1.315 | 1.286 | 1.349 | 1.340 | 1.264 |
| A/canine/Korea/KRIBB01/2011 | 1.334 | 1.434 | 1.437 | 1.325 | 1.284 | 1.347 | 1.338 | 1.260 |
| A/canine/Korea/GCVP01/2007 | 1.301 | 1.396 | 1.398 | 1.293 | 1.282 | 1.345 | 1.335 | 1.258 |
| A/canine/Korea/DG1/2014 | 1.302 | 1.398 | 1.394 | 1.292 | 1.273 | 1.339 | 1.328 | 1.249 |
| A/canine/Korea/CY053/2014 | 1.318 | 1.418 | 1.421 | 1.311 | 1.286 | 1.351 | 1.341 | 1.262 |
| A/canine/Korea/CY009/2010 | 1.315 | 1.412 | 1.416 | 1.309 | 1.277 | 1.340 | 1.331 | 1.254 |
| A/canine/Korea/BD1/2013 | 1.324 | 1.423 | 1.424 | 1.313 | 1.293 | 1.359 | 1.350 | 1.270 |
| A/canine/Korea/0589318/2015 | 1.320 | 1.421 | 1.424 | 1.310 | 1.330 | 1.400 | 1.392 | 1.309 |
| A/canine/Korea/01/2007 | 1.301 | 1.396 | 1.398 | 1.292 | 1.303 | 1.368 | 1.358 | 1.278 |
| A/canine/Jiangsu/06/2010 | 1.339 | 1.440 | 1.437 | 1.327 | 1.282 | 1.345 | 1.336 | 1.260 |
| A/canine/Jiangsu/05/2010 | 1.334 | 1.434 | 1.432 | 1.325 | 1.289 | 1.352 | 1.343 | 1.267 |
| A/canine/Jiangsu/04/2010 | 1.322 | 1.420 | 1.419 | 1.312 | 1.289 | 1.352 | 1.343 | 1.267 |
| A/canine/Jiangsu/03/2010 | 1.334 | 1.434 | 1.432 | 1.325 | 1.289 | 1.352 | 1.343 | 1.267 |
| A/canine/Jiangsu/02/2010 | 1.334 | 1.434 | 1.432 | 1.325 | 1.289 | 1.352 | 1.343 | 1.267 |
| A/canine/Jiangsu/01/2009 | 1.334 | 1.434 | 1.432 | 1.325 | 1.289 | 1.352 | 1.343 | 1.267 |
| A/canine/Indiana/96198/2015 | 1.317 | 1.417 | 1.419 | 1.309 | 1.328 | 1.398 | 1.389 | 1.307 |
| A/canine/Indiana/003018/2016 | 1.320 | 1.421 | 1.423 | 1.312 | 1.332 | 1.403 | 1.394 | 1.312 |
| A/canine/Illinois/1619144/2015 | 1.319 | 1.420 | 1.423 | 1.311 | 1.325 | 1.395 | 1.387 | 1.305 |
| A/canine/Illinois/328292/2015 | 1.317 | 1.419 | 1.421 | 1.310 | 1.328 | 1.398 | 1.390 | 1.307 |
| A/canine/Illinois/283066/2015 | 1.317 | 1.418 | 1.420 | 1.310 | 1.328 | 1.398 | 1.389 | 1.307 |
| A/canine/Illinois/077753/2016 | 1.334 | 1.437 | 1.440 | 1.326 | 1.330 | 1.401 | 1.391 | 1.309 |
| A/canine/Illinois/12191/2015 | 1.318 | 1.418 | 1.421 | 1.310 | 1.325 | 1.396 | 1.387 | 1.305 |
| A/canine/Heilongjiang/L1/2013 | 1.324 | 1.422 | 1.418 | 1.315 | 1.277 | 1.340 | 1.331 | 1.254 |
| A/canine/Guangdong/23/2012 | 1.339 | 1.440 | 1.436 | 1.328 | 1.266 | 1.329 | 1.320 | 1.244 |
| A/canine/Guangdong/12/2012 | 1.341 | 1.442 | 1.438 | 1.329 | 1.277 | 1.341 | 1.333 | 1.256 |
| A/canine/Guangdong/05/2011 | 1.341 | 1.443 | 1.441 | 1.330 | 1.282 | 1.344 | 1.336 | 1.259 |
| A/canine/Guangdong/3/2011 | 1.320 | 1.419 | 1.414 | 1.309 | 1.290 | 1.354 | 1.343 | 1.267 |
| A/canine/Guangdong/2/2011 | 1.322 | 1.422 | 1.418 | 1.310 | 1.285 | 1.347 | 1.340 | 1.263 |
| A/canine/Guangdong/2/2006 | 1.309 | 1.406 | 1.407 | 1.300 | 1.282 | 1.345 | 1.332 | 1.258 |
| A/canine/Guangdong/1/2007 | 1.309 | 1.406 | 1.407 | 1.300 | 1.273 | 1.334 | 1.323 | 1.250 |
| A/canine/Guangdong/1/2006 | 1.300 | 1.396 | 1.398 | 1.293 | 1.282 | 1.342 | 1.327 | 1.257 |
| A/canine/Georgia/104940/2015 | 1.319 | 1.420 | 1.423 | 1.311 | 1.330 | 1.402 | 1.393 | 1.309 |
| A/canine/Georgia/95391/2015 | 1.319 | 1.420 | 1.423 | 1.311 | 1.327 | 1.399 | 1.390 | 1.307 |
| A/canine/Florida/269770/2015 | 1.328 | 1.430 | 1.431 | 1.320 | 1.330 | 1.402 | 1.394 | 1.309 |
| A/canine/China/JLM2/2015 | 1.383 | 1.493 | 1.487 | 1.369 | 1.277 | 1.341 | 1.332 | 1.253 |
| A/canine/China/JLM1/2015 | 1.380 | 1.489 | 1.481 | 1.368 | 1.271 | 1.335 | 1.326 | 1.248 |
| Average | 1.326 | 1.427 | 1.427 | 1.317 | 1.298 | 1.364 | 1.355 | 1.276 |
| SD | 0.017 | 0.020 | 0.018 | 0.016 | 0.023 | 0.027 | 0.027 | 0.024 |
|  | NS | | | | PA | | | |
| Strain name | *Homo sapiens* | *Canis familiaris* | *Felis catus* | *Gallus gallus* | *Homo sapiens* | *Canis familiaris* | *Felis catus* | *Gallus gallus* |
| A/canine/Zhejiang/1/2010 | 1.350 | 1.433 | 1.426 | 1.324 | 1.244 | 1.312 | 1.299 | 1.223 |
| A/canine/Wisconsin/19137/2016 | 1.396 | 1.491 | 1.485 | 1.370 | 1.260 | 1.330 | 1.317 | 1.242 |
| A/canine/Texas/343907/2015 | 1.399 | 1.494 | 1.487 | 1.373 | 1.259 | 1.329 | 1.317 | 1.240 |
| A/canine/South_Korea/0173915/2015 | 1.391 | 1.485 | 1.478 | 1.361 | 1.248 | 1.319 | 1.307 | 1.229 |
| A/canine/North_Carolina/109904/2015 | 1.399 | 1.494 | 1.487 | 1.373 | 1.252 | 1.320 | 1.307 | 1.232 |
| A/canine/Liaoning/H6/2012 | 1.374 | 1.463 | 1.455 | 1.348 | 1.251 | 1.319 | 1.305 | 1.231 |
| A/canine/Liaoning/27/2012 | 1.374 | 1.463 | 1.455 | 1.348 | 1.247 | 1.315 | 1.302 | 1.226 |
| A/canine/Korea/S1/2012 | 1.361 | 1.448 | 1.440 | 1.335 | 1.239 | 1.306 | 1.294 | 1.220 |
| A/canine/Korea/KRIBB01/2011 | 1.372 | 1.464 | 1.457 | 1.345 | 1.241 | 1.308 | 1.296 | 1.221 |
| A/canine/Korea/GCVP01/2007 | 1.314 | 1.391 | 1.387 | 1.290 | 1.232 | 1.298 | 1.285 | 1.210 |
| A/canine/Korea/DG1/2014 | 1.378 | 1.468 | 1.461 | 1.352 | 1.242 | 1.311 | 1.298 | 1.221 |
| A/canine/Korea/CY053/2014 | 1.382 | 1.469 | 1.463 | 1.357 | 1.237 | 1.304 | 1.291 | 1.217 |
| A/canine/Korea/CY009/2010 | 1.360 | 1.443 | 1.432 | 1.332 | 1.237 | 1.303 | 1.290 | 1.216 |
| A/canine/Korea/BD1/2013 | 1.378 | 1.471 | 1.465 | 1.349 | 1.236 | 1.301 | 1.288 | 1.215 |
| A/canine/Korea/0589318/2015 | 1.389 | 1.483 | 1.475 | 1.360 | 1.247 | 1.317 | 1.304 | 1.228 |
| A/canine/Korea/01/2007 | 1.327 | 1.406 | 1.396 | 1.299 | 1.227 | 1.292 | 1.279 | 1.206 |
| A/canine/Jiangsu/06/2010 | 1.353 | 1.440 | 1.433 | 1.328 | 1.252 | 1.322 | 1.309 | 1.231 |
| A/canine/Jiangsu/05/2010 | 1.366 | 1.451 | 1.444 | 1.337 | 1.252 | 1.322 | 1.309 | 1.231 |
| A/canine/Jiangsu/04/2010 | 1.366 | 1.451 | 1.444 | 1.337 | 1.253 | 1.321 | 1.308 | 1.231 |
| A/canine/Jiangsu/03/2010 | 1.366 | 1.451 | 1.444 | 1.337 | 1.252 | 1.321 | 1.307 | 1.231 |
| A/canine/Jiangsu/02/2010 | 1.345 | 1.430 | 1.424 | 1.319 | 1.253 | 1.321 | 1.308 | 1.231 |
| A/canine/Jiangsu/01/2009 | 1.366 | 1.451 | 1.444 | 1.337 | 1.253 | 1.321 | 1.308 | 1.231 |
| A/canine/Indiana/96198/2015 | 1.399 | 1.494 | 1.487 | 1.373 | 1.261 | 1.331 | 1.319 | 1.242 |
| A/canine/Indiana/003018/2016 | 1.399 | 1.494 | 1.487 | 1.373 | 1.257 | 1.326 | 1.315 | 1.239 |
| A/canine/Illinois/1619144/2015 | 1.399 | 1.494 | 1.487 | 1.373 | 1.257 | 1.327 | 1.313 | 1.237 |
| A/canine/Illinois/328292/2015 | 1.399 | 1.494 | 1.487 | 1.373 | 1.259 | 1.329 | 1.317 | 1.240 |
| A/canine/Illinois/283066/2015 | 1.399 | 1.494 | 1.487 | 1.373 | 1.263 | 1.333 | 1.321 | 1.244 |
| A/canine/Illinois/077753/2016 | 1.399 | 1.494 | 1.487 | 1.373 | 1.255 | 1.325 | 1.313 | 1.238 |
| A/canine/Illinois/12191/2015 | 1.399 | 1.494 | 1.487 | 1.373 | 1.251 | 1.320 | 1.308 | 1.231 |
| A/canine/Heilongjiang/L1/2013 | 1.367 | 1.455 | 1.448 | 1.342 | 1.251 | 1.320 | 1.307 | 1.231 |
| A/canine/Guangdong/23/2012 | 1.368 | 1.454 | 1.446 | 1.340 | 1.261 | 1.332 | 1.318 | 1.241 |
| A/canine/Guangdong/12/2012 | 1.368 | 1.454 | 1.446 | 1.340 | 1.260 | 1.331 | 1.317 | 1.240 |
| A/canine/Guangdong/05/2011 | 1.378 | 1.460 | 1.453 | 1.348 | 1.251 | 1.322 | 1.308 | 1.230 |
| A/canine/Guangdong/3/2011 | 1.368 | 1.453 | 1.442 | 1.337 | 1.234 | 1.301 | 1.288 | 1.209 |
| A/canine/Guangdong/2/2011 | 1.363 | 1.447 | 1.440 | 1.333 | 1.264 | 1.333 | 1.320 | 1.239 |
| A/canine/Guangdong/2/2006 | 1.361 | 1.447 | 1.440 | 1.335 | 1.226 | 1.290 | 1.280 | 1.206 |
| A/canine/Guangdong/1/2007 | 1.348 | 1.434 | 1.427 | 1.322 | 1.228 | 1.292 | 1.281 | 1.206 |
| A/canine/Guangdong/1/2006 | 1.342 | 1.427 | 1.422 | 1.314 | 1.223 | 1.285 | 1.273 | 1.201 |
| A/canine/Georgia/104940/2015 | 1.399 | 1.494 | 1.487 | 1.373 | 1.247 | 1.314 | 1.300 | 1.228 |
| A/canine/Georgia/95391/2015 | 1.399 | 1.494 | 1.487 | 1.373 | 1.251 | 1.319 | 1.306 | 1.231 |
| A/canine/Florida/269770/2015 | 1.399 | 1.494 | 1.487 | 1.373 | 1.250 | 1.318 | 1.305 | 1.230 |
| A/canine/China/JLM2/2015 | 1.392 | 1.483 | 1.468 | 1.360 | 1.249 | 1.320 | 1.311 | 1.228 |
| A/canine/China/JLM1/2015 | 1.396 | 1.490 | 1.476 | 1.365 | 1.253 | 1.325 | 1.315 | 1.232 |
| Average | 1.376 | 1.465 | 1.457 | 1.348 | 1.248 | 1.316 | 1.304 | 1.228 |
| SD | 0.022 | 0.026 | 0.026 | 0.022 | 0.011 | 0.013 | 0.012 | 0.011 |
|  | PB1 | | | | PB2 | | | |
| Strain name | *Homo sapiens* | *Canis familiaris* | *Felis catus* | *Gallus gallus* | *Homo sapiens* | *Canis familiaris* | *Felis catus* | *Gallus gallus* |
| A/canine/Zhejiang/1/2010 | 1.253 | 1.319 | 1.316 | 1.227 | 1.281 | 1.368 | 1.360 | 1.248 |
| A/canine/Wisconsin/19137/2016 | 1.289 | 1.366 | 1.361 | 1.262 | 1.327 | 1.426 | 1.417 | 1.290 |
| A/canine/Texas/343907/2015 | 1.286 | 1.362 | 1.358 | 1.259 | 1.326 | 1.425 | 1.415 | 1.289 |
| A/canine/South_Korea/0173915/2015 | 1.275 | 1.349 | 1.346 | 1.249 | 1.314 | 1.410 | 1.398 | 1.279 |
| A/canine/North_Carolina/109904/2015 | 1.280 | 1.355 | 1.351 | 1.255 | 1.331 | 1.429 | 1.419 | 1.296 |
| A/canine/Liaoning/H6/2012 | 1.282 | 1.355 | 1.350 | 1.255 | 1.317 | 1.415 | 1.407 | 1.282 |
| A/canine/Liaoning/27/2012 | 1.283 | 1.356 | 1.351 | 1.255 | 1.314 | 1.411 | 1.404 | 1.280 |
| A/canine/Korea/S1/2012 | 1.252 | 1.320 | 1.315 | 1.225 | 1.285 | 1.372 | 1.360 | 1.251 |
| A/canine/Korea/KRIBB01/2011 | 1.246 | 1.314 | 1.312 | 1.220 | 1.298 | 1.388 | 1.379 | 1.265 |
| A/canine/Korea/GCVP01/2007 | 1.220 | 1.281 | 1.280 | 1.193 | 1.284 | 1.372 | 1.363 | 1.251 |
| A/canine/Korea/DG1/2014 | 1.261 | 1.329 | 1.324 | 1.234 | 1.301 | 1.392 | 1.379 | 1.265 |
| A/canine/Korea/CY053/2014 | 1.249 | 1.318 | 1.314 | 1.221 | 1.291 | 1.379 | 1.369 | 1.258 |
| A/canine/Korea/CY009/2010 | 1.246 | 1.313 | 1.312 | 1.219 | 1.286 | 1.374 | 1.364 | 1.254 |
| A/canine/Korea/BD1/2013 | 1.269 | 1.342 | 1.339 | 1.244 | 1.293 | 1.385 | 1.375 | 1.258 |
| A/canine/Korea/0589318/2015 | 1.274 | 1.347 | 1.344 | 1.248 | 1.314 | 1.410 | 1.398 | 1.279 |
| A/canine/Korea/01/2007 | 1.217 | 1.277 | 1.276 | 1.191 | 1.284 | 1.372 | 1.363 | 1.251 |
| A/canine/Jiangsu/06/2010 | 1.268 | 1.338 | 1.334 | 1.241 | 1.281 | 1.370 | 1.365 | 1.249 |
| A/canine/Jiangsu/05/2010 | 1.257 | 1.324 | 1.320 | 1.230 | 1.280 | 1.367 | 1.360 | 1.246 |
| A/canine/Jiangsu/04/2010 | 1.259 | 1.326 | 1.321 | 1.232 | 1.281 | 1.370 | 1.363 | 1.248 |
| A/canine/Jiangsu/03/2010 | 1.257 | 1.324 | 1.319 | 1.230 | 1.270 | 1.355 | 1.347 | 1.237 |
| A/canine/Jiangsu/02/2010 | 1.258 | 1.325 | 1.320 | 1.231 | 1.280 | 1.367 | 1.360 | 1.246 |
| A/canine/Jiangsu/01/2009 | 1.257 | 1.324 | 1.318 | 1.231 | 1.280 | 1.367 | 1.360 | 1.246 |
| A/canine/Indiana/96198/2015 | 1.278 | 1.352 | 1.348 | 1.252 | 1.320 | 1.417 | 1.407 | 1.283 |
| A/canine/Indiana/003018/2016 | 1.285 | 1.362 | 1.357 | 1.259 | 1.326 | 1.425 | 1.415 | 1.289 |
| A/canine/Illinois/1619144/2015 | 1.280 | 1.355 | 1.351 | 1.254 | 1.314 | 1.410 | 1.400 | 1.279 |
| A/canine/Illinois/328292/2015 | 1.286 | 1.362 | 1.358 | 1.259 | 1.326 | 1.425 | 1.415 | 1.289 |
| A/canine/Illinois/283066/2015 | 1.282 | 1.357 | 1.353 | 1.257 | 1.321 | 1.418 | 1.409 | 1.285 |
| A/canine/Illinois/077753/2016 | 1.288 | 1.365 | 1.361 | 1.261 | 1.325 | 1.423 | 1.414 | 1.288 |
| A/canine/Illinois/12191/2015 | 1.280 | 1.355 | 1.351 | 1.255 | 1.317 | 1.414 | 1.403 | 1.282 |
| A/canine/Heilongjiang/L1/2013 | 1.266 | 1.337 | 1.334 | 1.239 | 1.301 | 1.395 | 1.387 | 1.267 |
| A/canine/Guangdong/23/2012 | 1.281 | 1.355 | 1.349 | 1.253 | 1.312 | 1.407 | 1.400 | 1.279 |
| A/canine/Guangdong/12/2012 | 1.274 | 1.347 | 1.342 | 1.247 | 1.307 | 1.401 | 1.395 | 1.274 |
| A/canine/Guangdong/05/2011 | 1.245 | 1.309 | 1.305 | 1.217 | 1.285 | 1.374 | 1.366 | 1.251 |
| A/canine/Guangdong/3/2011 | 1.237 | 1.304 | 1.301 | 1.212 | 1.283 | 1.371 | 1.363 | 1.248 |
| A/canine/Guangdong/2/2011 | 1.228 | 1.293 | 1.289 | 1.201 | 1.284 | 1.372 | 1.365 | 1.250 |
| A/canine/Guangdong/2/2006 | 1.228 | 1.295 | 1.291 | 1.201 | 1.277 | 1.363 | 1.354 | 1.244 |
| A/canine/Guangdong/1/2007 | 1.228 | 1.293 | 1.289 | 1.201 | 1.284 | 1.370 | 1.362 | 1.252 |
| A/canine/Guangdong/1/2006 | 1.253 | 1.323 | 1.322 | 1.228 | 1.270 | 1.355 | 1.349 | 1.237 |
| A/canine/Georgia/104940/2015 | 1.280 | 1.355 | 1.351 | 1.255 | 1.326 | 1.423 | 1.414 | 1.291 |
| A/canine/Georgia/95391/2015 | 1.280 | 1.355 | 1.351 | 1.255 | 1.328 | 1.426 | 1.416 | 1.293 |
| A/canine/Florida/269770/2015 | 1.280 | 1.355 | 1.351 | 1.255 | 1.329 | 1.426 | 1.416 | 1.293 |
| A/canine/China/JLM2/2015 | 1.277 | 1.352 | 1.344 | 1.251 | 1.302 | 1.397 | 1.394 | 1.269 |
| A/canine/China/JLM1/2015 | 1.277 | 1.352 | 1.344 | 1.251 | 1.303 | 1.398 | 1.395 | 1.270 |
| Average | 1.264 | 1.335 | 1.331 | 1.238 | 1.301 | 1.394 | 1.385 | 1.267 |
| SD | 0.020 | 0.024 | 0.024 | 0.020 | 0.019 | 0.024 | 0.023 | 0.018 |
