# Supplementary figures and images for: Genetic and evolutionary analysis of emerging H3N2 canine influenza virus
Source: Emerg Microbes Infect. 2018 Apr 25;7:73. doi: 10.1038/s41426-018-0079-0 (PMC5915587; doi:10.1038/s41426-018-0079-0)

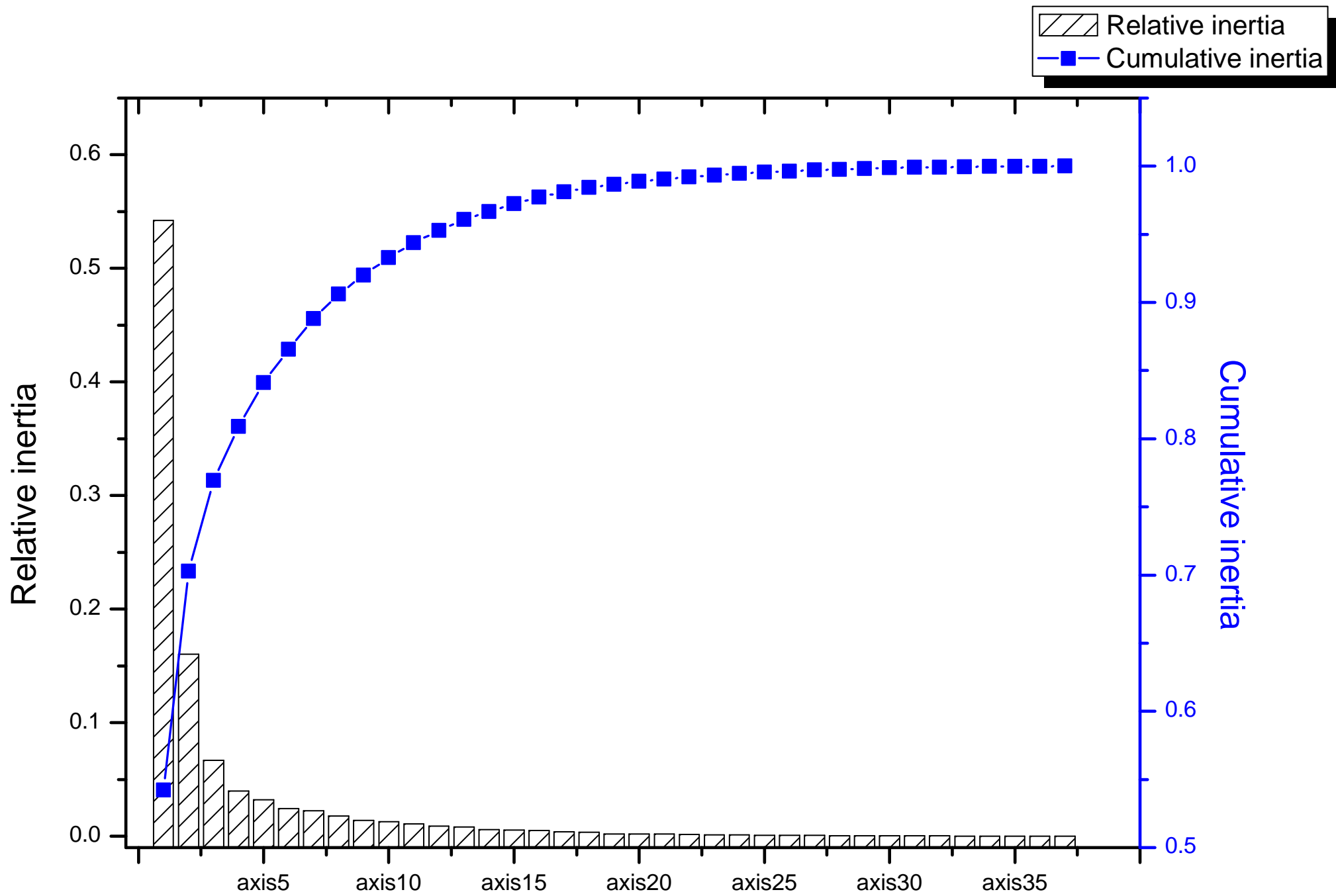

Supplement: Supplementary file 2 — FIGURE S1 [file 41426_2018_79_MOESM2_ESM.pdf]

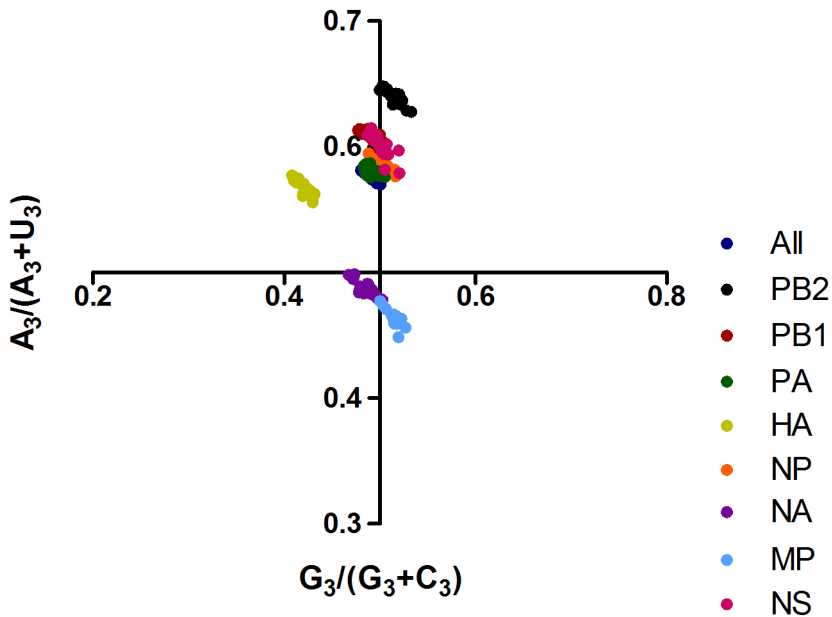

Supplement: Supplementary file 3 — FIGURE S2 [file 41426_2018_79_MOESM3_ESM.pdf]

# PB2

—●— All 
 —●— Origin 
 —●— China 
 —●— Korea/USA

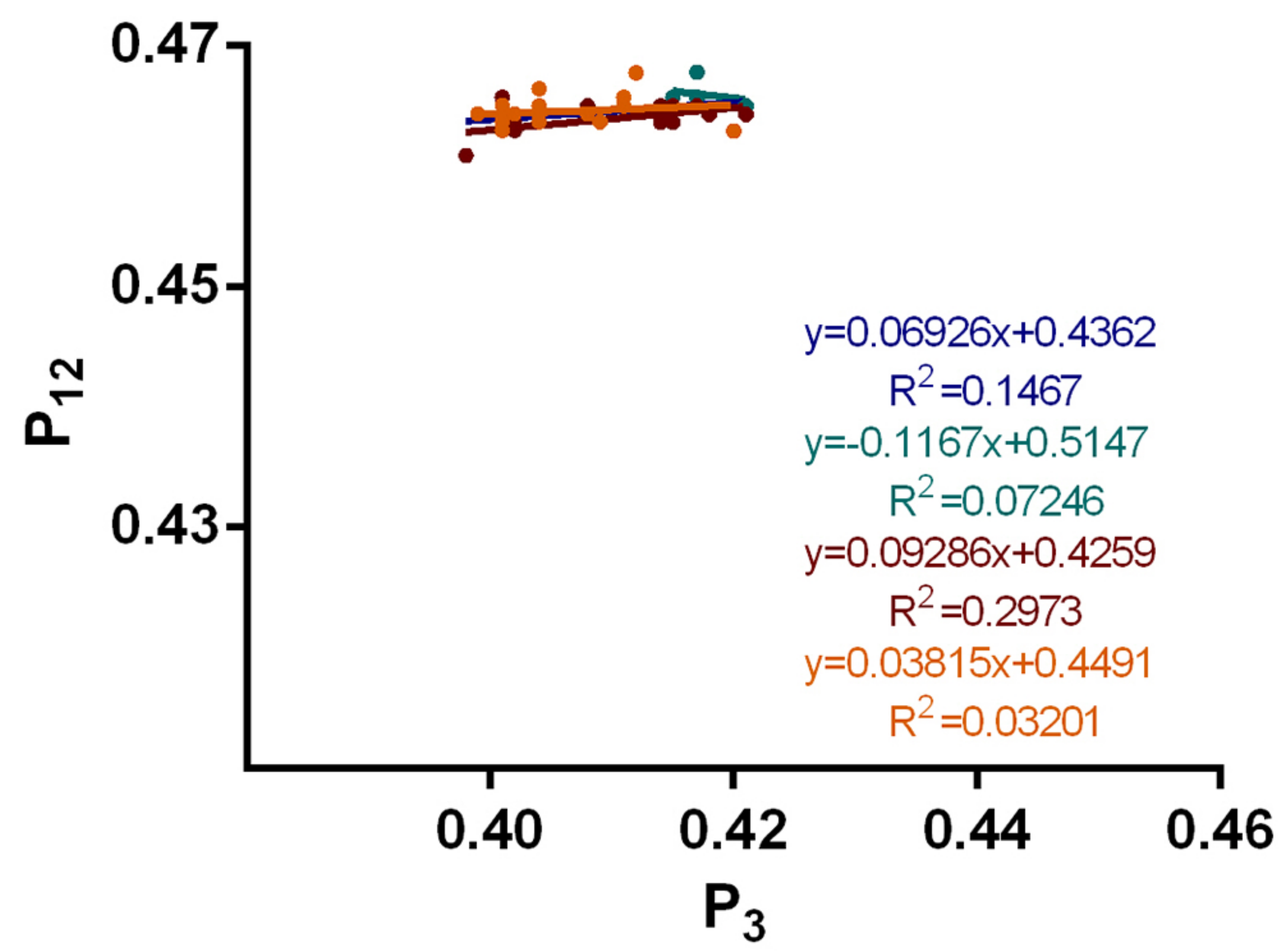

# PB1

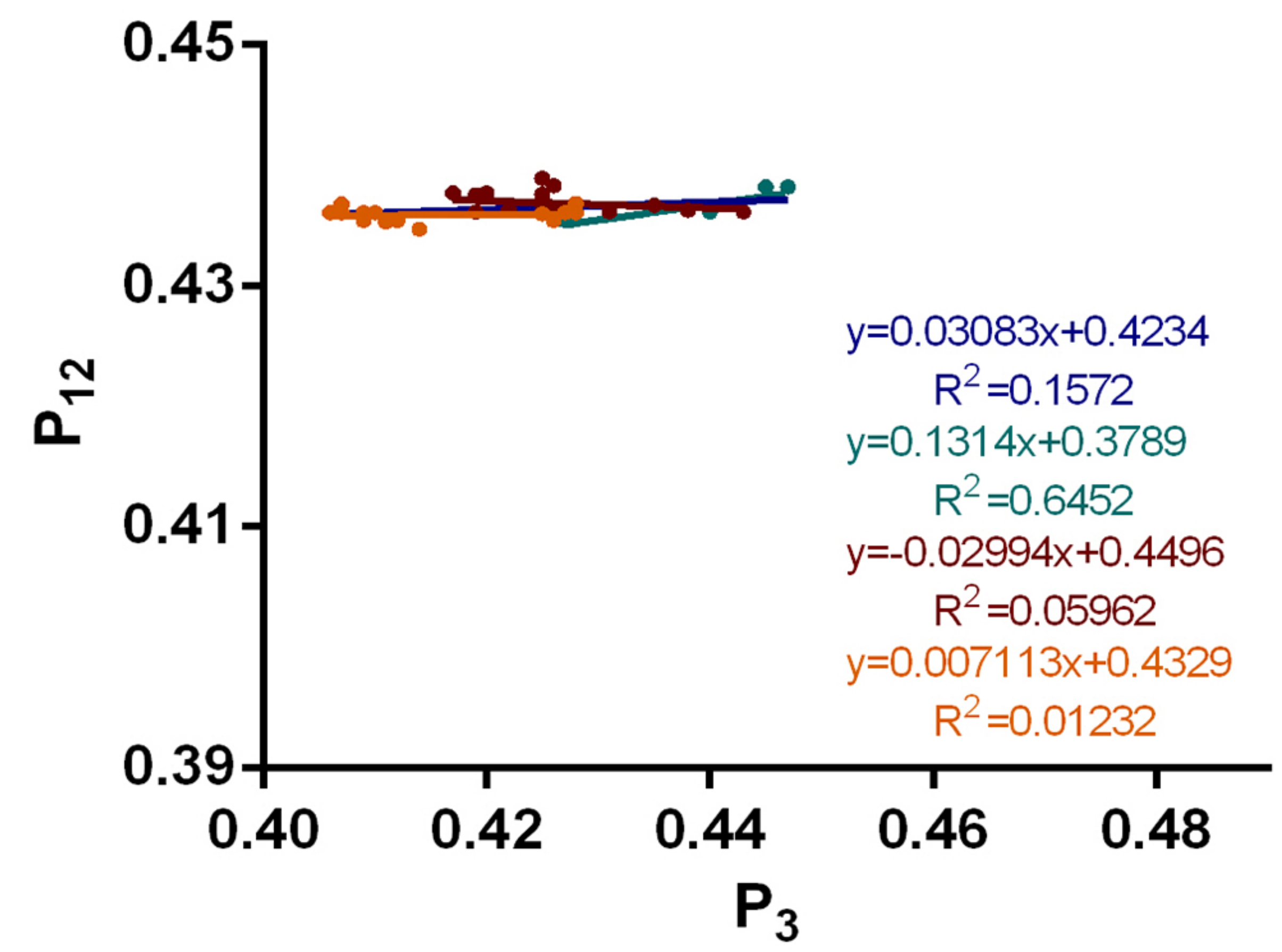

# PA

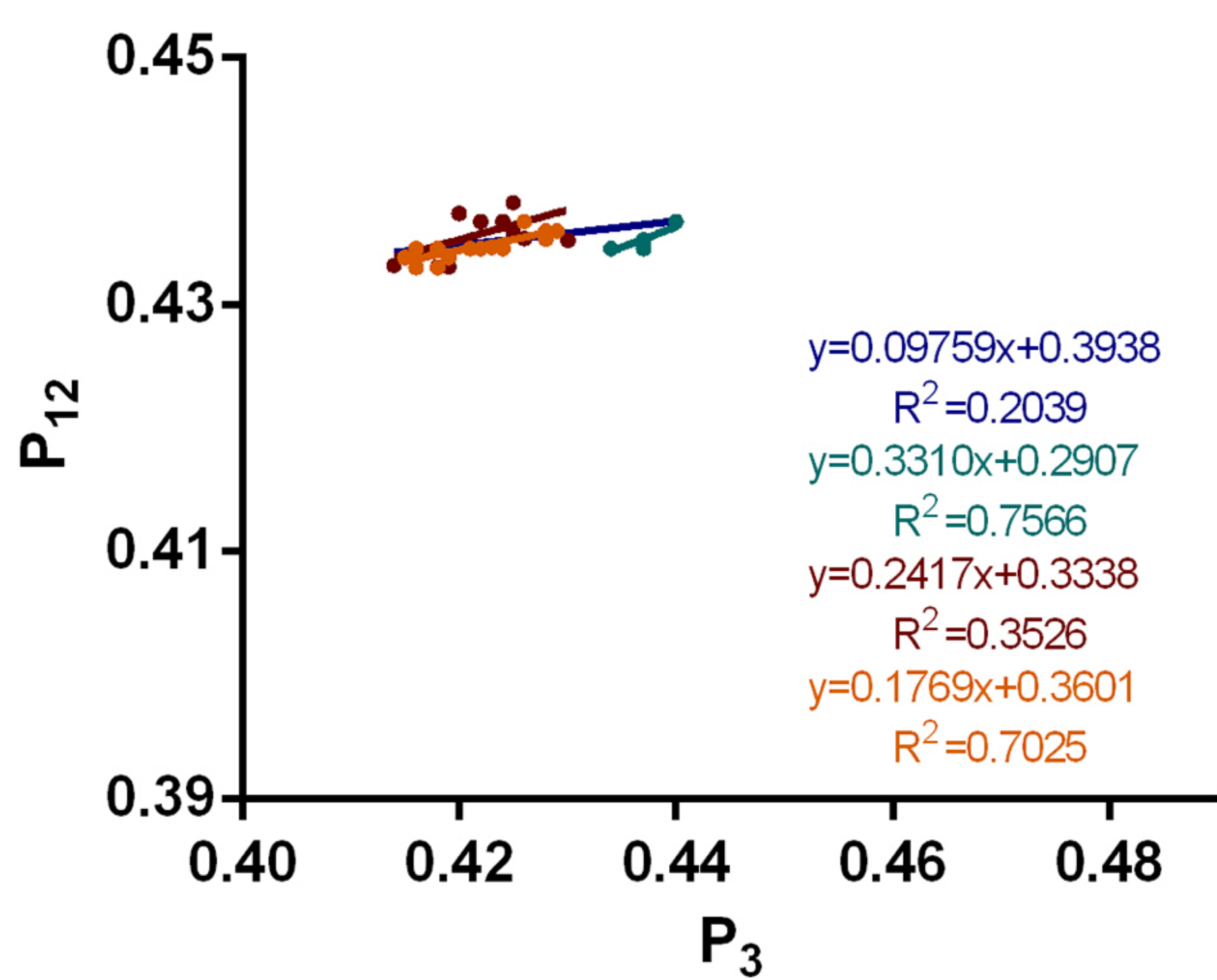

# HA

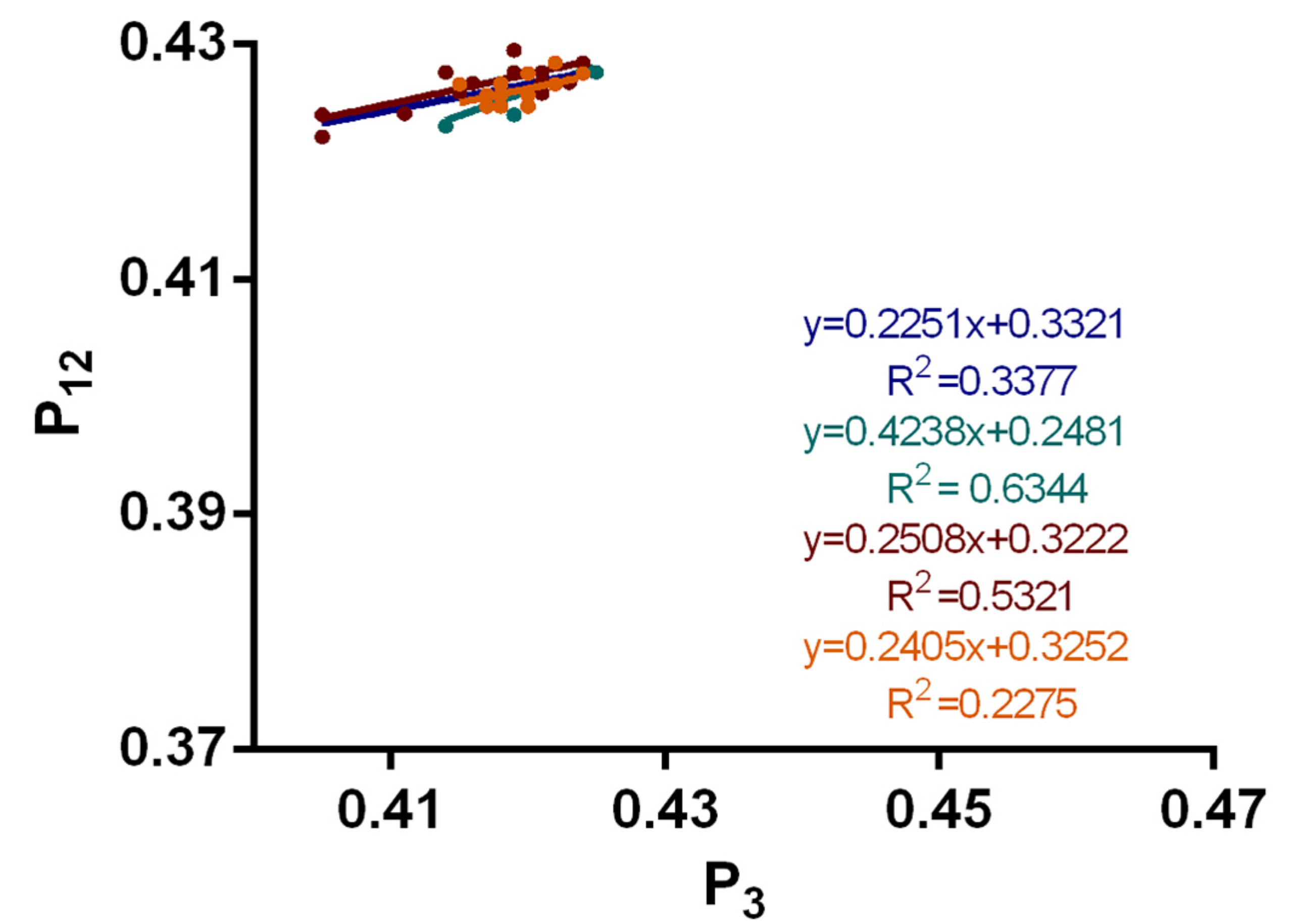

# NP

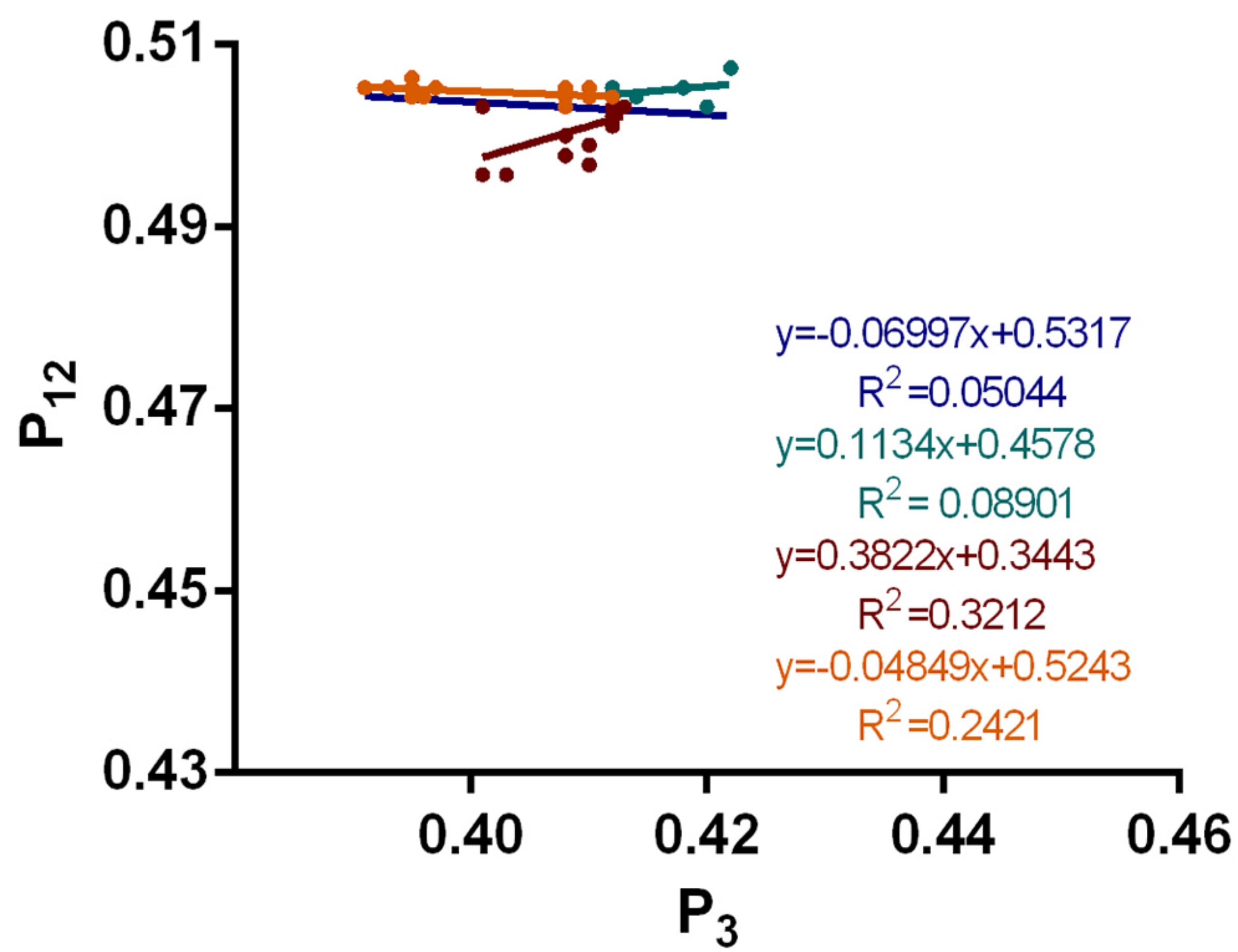

# NA

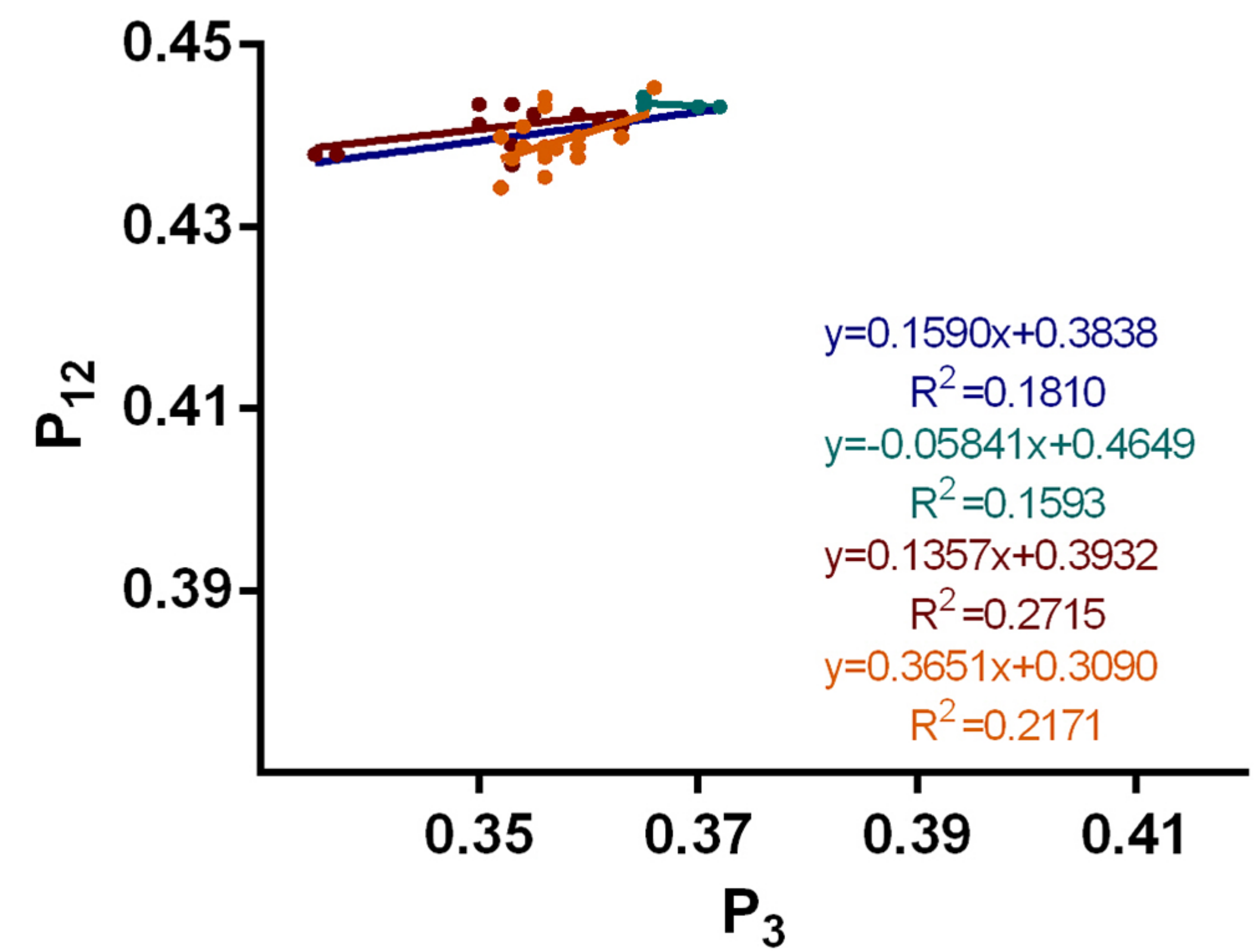

# MP

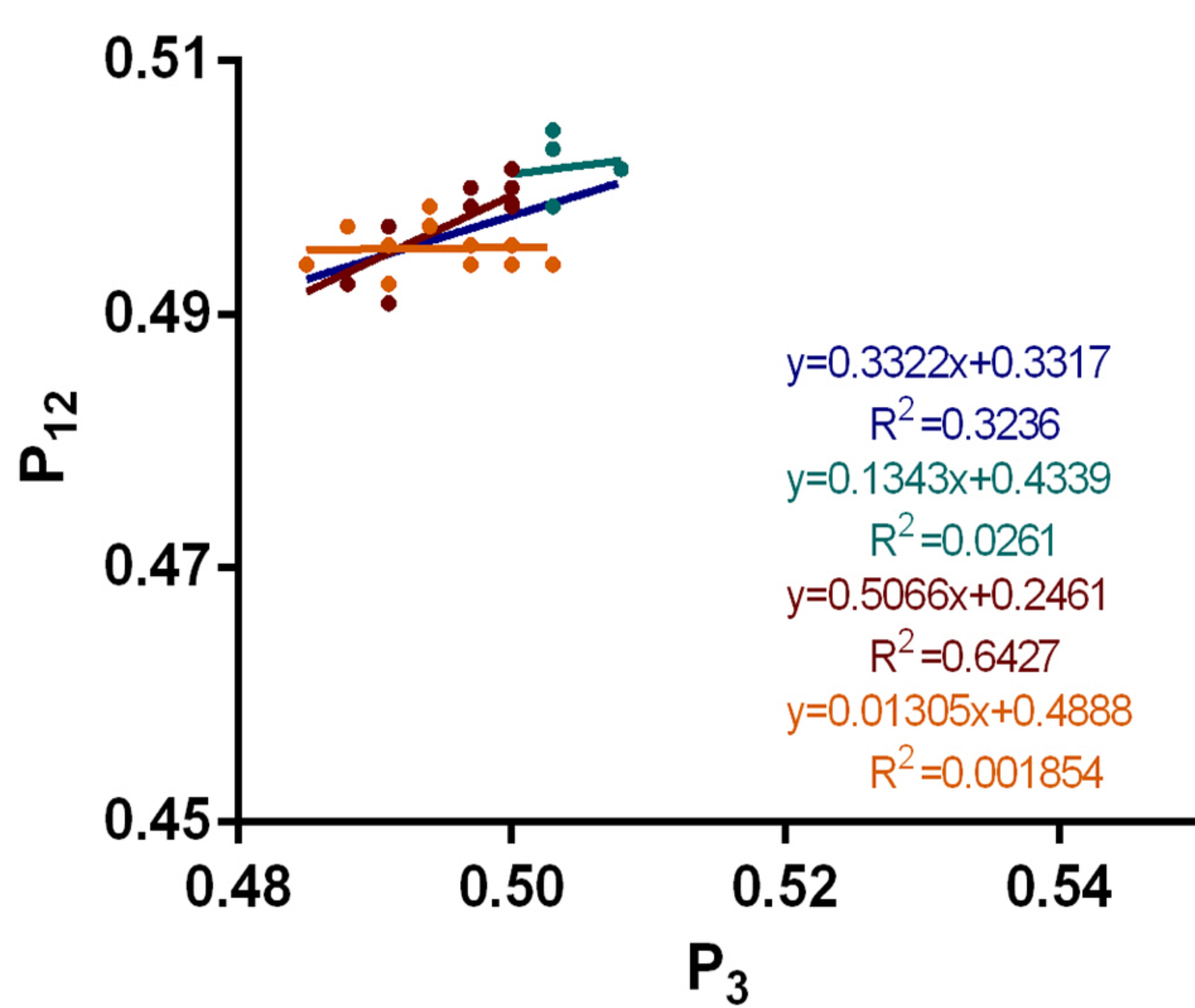

# NS

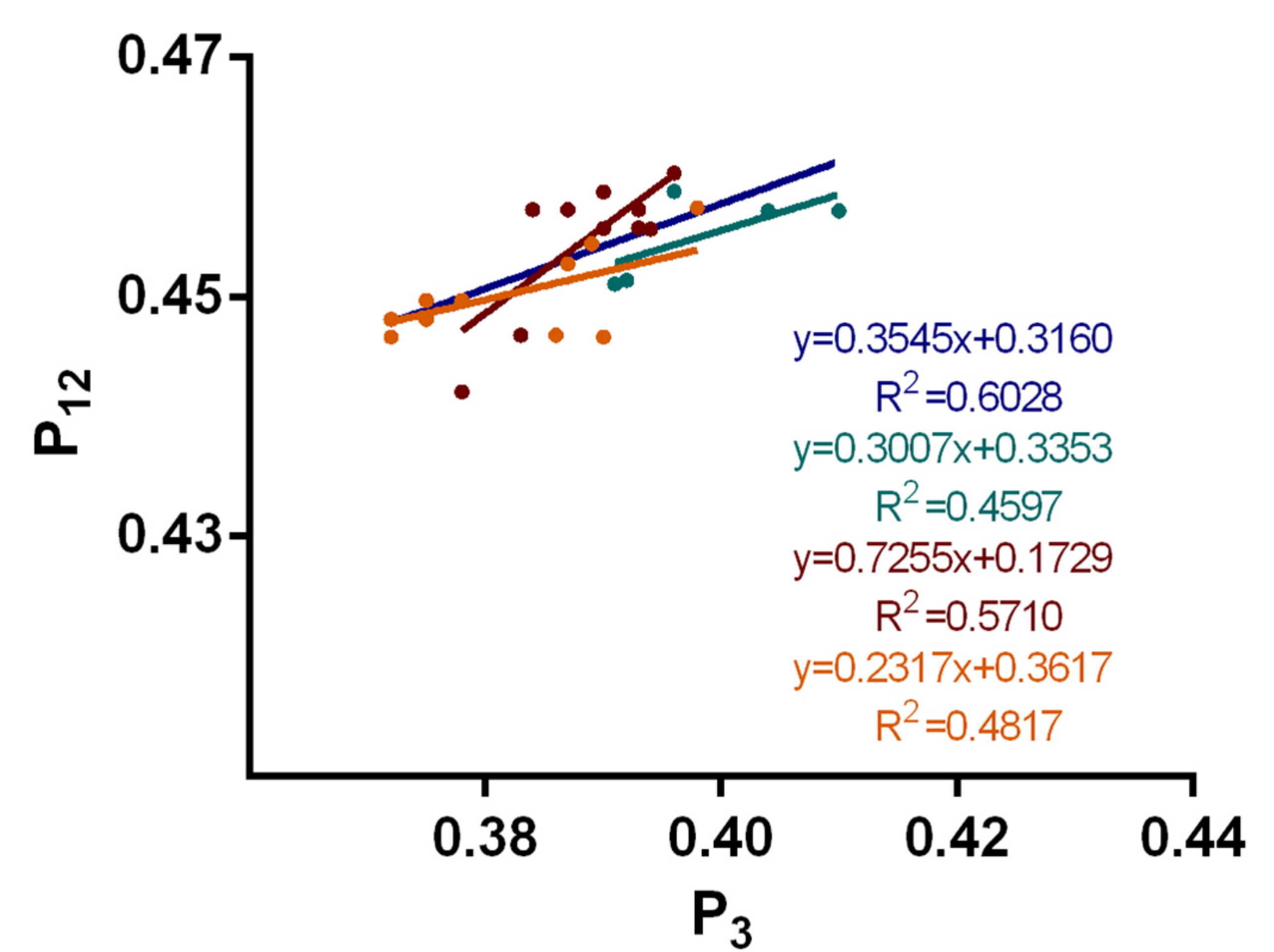

Supplement: Supplementary file 4 — FIGURE S3 [file 41426_2018_79_MOESM4_ESM.pdf]

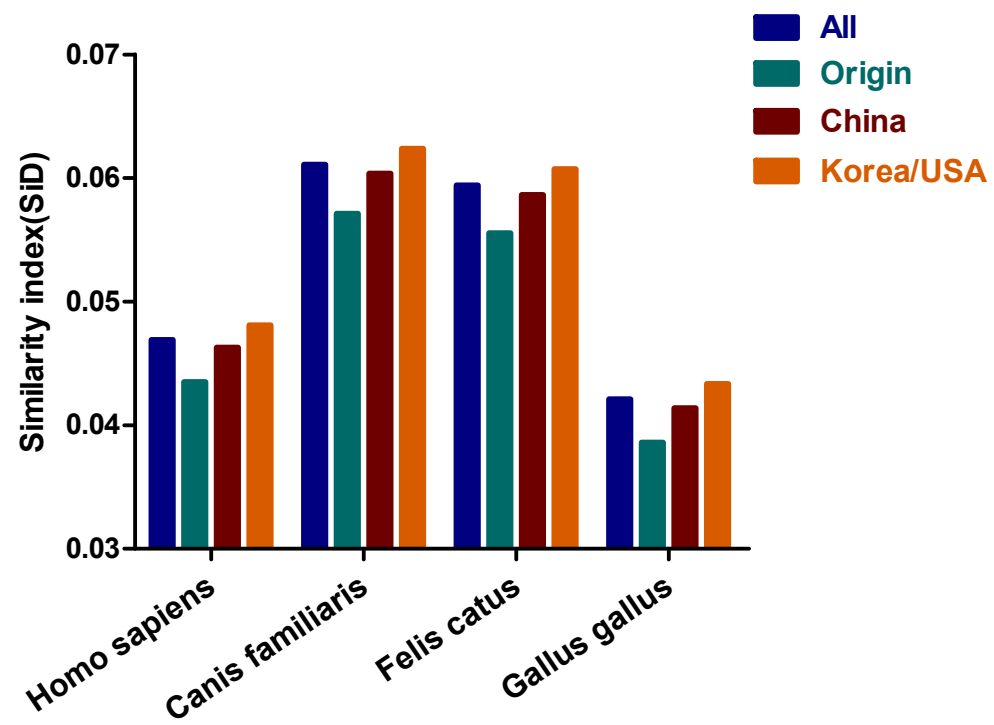

Supplement: Supplementary file 5 — FIGURE S4 [file 41426_2018_79_MOESM5_ESM.pdf]

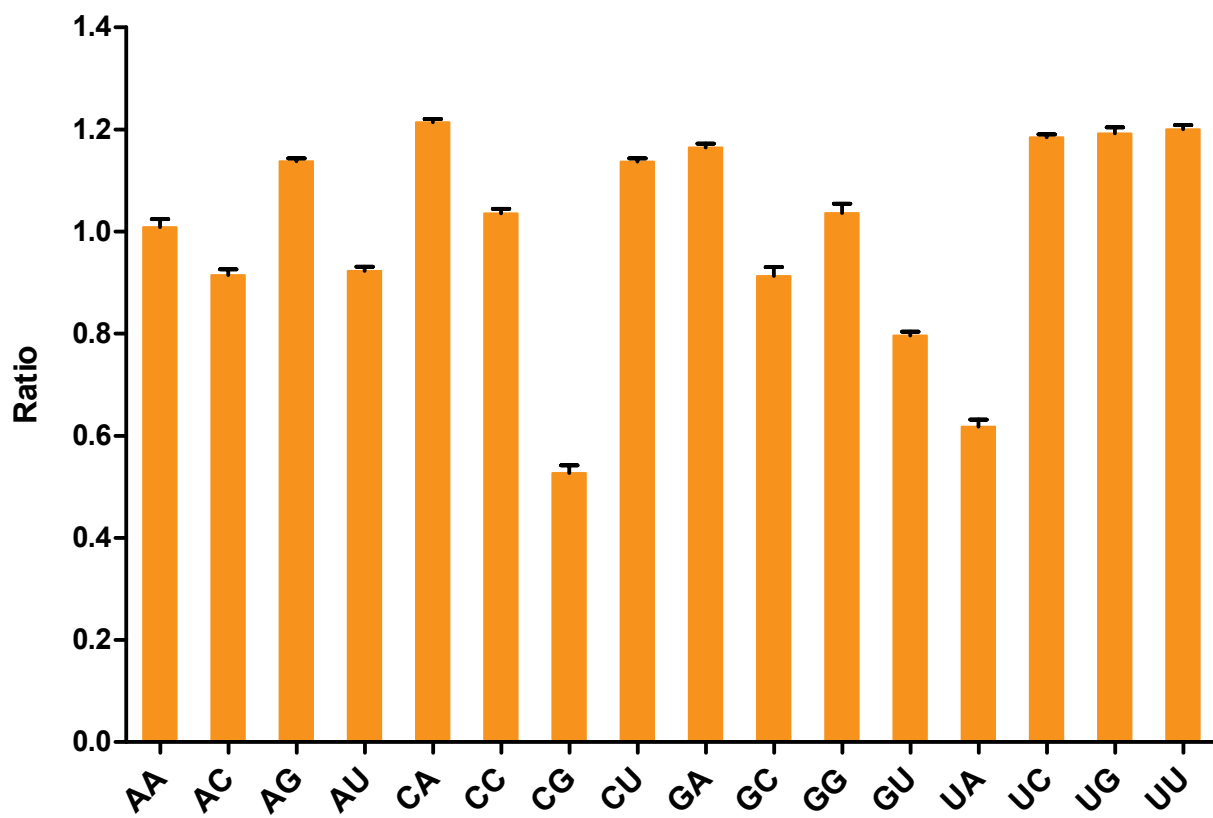

Supplement: Supplementary file 6 — FIGURE S5 [file 41426_2018_79_MOESM6_ESM.pdf]
